# Metasurface-based multifunctional composites with ultra-robust broadband microwave absorption up to 1000 °C

**DOI:** 10.1038/s41467-025-66317-3

**Published:** 2025-11-25

**Authors:** Xinyuan Lv, Qiujin Gu, Shengchi Zhu, Xun Sun, Minglong Yang, Tao Liu, Yunpeng Ma, Zhenxin Cao, Haitao Liu

**Affiliations:** 1 Science and Technology on Advanced Ceramic Fibers and Composites Laboratory, College of Aerospace Science and Engineering, National University of Defense Technology, Changsha, P.R. China; 2 State Key Laboratory of Millimeter Waves, Southeast University, Nanjing, P.R. China; 3 School of Aeronautical Science and Engineering, BeiHang University, Beijing, P.R. China

**Keywords:** Metamaterials, Composites, Electronic properties and materials, Design, synthesis and processing

## Abstract

The metamaterials offer methodology and infinite possibilities for ultra-broadband microwave absorption (MA). However, maintaining stable broadband MA under extreme high-temperature environments remains one of the most cutting-edge challenges. Herein, we report a RuO_2_/glass resistive material for the fabrication of microwave-absorbing metasurfaces. Based on tunneling effect, an ultra-low temperature coefficient of resistance was achieved in RuO_2_/glass, resulting in temperature-insensitivity of its electrical properties, thereby ensuring stability of MA properties of metasurfaces against temperature. Furthermore, using low-dielectric alumina aerogel composites and Al_2_O_3f_/Al_2_O_3_ ceramic composites as dielectric spacer layer, we propose the multifunctional composites integrated with MA, thermal insulation and load-bearing (MTL). The MTL integrated composites show an impressive broadband (2~12 GHz) MA performance that is ultra-robust against temperature variations (25~1000 °C), thermal shock (50 cycles at 25~1000 °C), incidence angle (±45°) and polarization. Additionally, the integrated composites also demonstrate a long-term thermal insulation ability and a high compressive modulus (6.58 MPa). This advancement provides insights for developing MA materials that can work in extreme multi-field environments.

## Abstract (web summary)

A RuO_2_/glass metasurface with low temperature coefficient of resistance is proposed based on tunneling effect. The metasurfaces show ultra-robust broadband microwave absorption at extreme environments up to 1000 °C.

## Introduction

The quest for on-demand manipulation of electromagnetic (EM) waves has driven the emergence of EM metamaterials (EMMs)^1,2^. Through the replacement of characteristic nanoscale atomic or molecular units with subwavelength artificial meta-atoms, EMMs demonstrate extraordinary EM performance beyond the intrinsic limits of natural materials^3,4^. Microwave-absorbing metasurfaces (MAMSs), as a transformative subclass of EMMs, can broad the absorption bandwidth through a carefully designed EM multi-resonance response. Owing to the tunability of the equivalent impedance, a MAMS can fundamentally break the inherent conflict between impedance matching and microwave loss^5^. Furthermore, due to its negligible thickness, a MAMS can provide much better integrability and mechanical flexibility, which has sparked a technological revolution in microwave-absorbing materials. To achieve broadband absorption, the resonant units of MAMSs are typically constructed from either metallic-based^6–8^ or carbon-based^9–11^ materials through topological design and encoding arrangement^12,13^. However, existing MAMSs maintain stable microwave-absorbing performance exclusively in low-temperature environments (typically ≤500 °C)^14,15^. The development of robust MAMSs capable of withstanding extreme environments, including sustained high-temperature exposure (≥1000 °C), large thermal gradients, and rapid thermal shocks, remains one of the most cutting-edge challenges.

The degradation of the microwave-absorbing properties of a MAMS at elevated temperatures stems from the instability of the magnetic/electrical properties of the resonant units. The most widely used magnetic absorber gradually lose their magnetic loss ability and become completely demagnetized at the Curie temperature (generally <800 °C), accompanied by oxidation problems. For resistive materials that absorb microwave through electrical losses, the temperature greatly influences both their carrier transport rate and dipole relaxation time, thereby inducing markedly variations of their electrical properties along with temperatures^16^. However, the design window of electrical parameters required for optimal microwave absorption is typically narrow. Thus, the detrimental impact of temperature on microwave-absorbing properties essentially arises from the inherent conflict between the large variation in electrical parameters and the narrow design window of electrical parameters. Although the design window of the electrical parameters of metasurfaces can be broadened via topological optimization of resonant units, the present resistive materials still show excessive electrical property drift (ranging from one-fold to tenfold variations) at temperatures as high as 1000 °C. The most common lanthanum strontium manganite (LaSrMnO_3_) resistive materials exhibit resistivity variations of up to one order of magnitude at 1000 °C^17^. Therefore, there is still a gap in the design and preparation of MAMSs that can maintain stable broadband absorption at high temperatures.

To reduce the variations of electrical properties of MAMSs at elevated temperatures, a natural and general strategy is to lower the temperature of metasurfaces using heat-insulating materials as their dielectric spacer layer^18,19^. For example, under some one-sided heating cases, such as those in aerodynamic heating, ceramic fiber felts or aerogels are usually selected as the dielectric spacer layer, and the MAMS is placed in low-temperature zone. However, if excellent microwave absorption and cooling performance are simultaneously pursued, then the overall thickness of the absorbing structure must be dramatically increased (generally >30 mm). In the case of a limited thickness, either the metasurface cannot be cooled to the desired temperature or the resonant frequency is shifted toward higher frequencies, deviating from the target absorption range. In a recent study, the heat-insulated SiO_2_ fiber paper was used as dielectric layer of a metasurface-based microwave-absorbing structure^18^. Nevertheless, the microwave-absorbing properties of the composites was not desirable, achieving effective absorption bandwidth (EAB) (EAB, bandwidth of reflectivity < −10 dB) of only 3.1 GHz at 1000 °C. Then, they optimized the sheet resistivity of metasurface and the interlayer layout, thereby expanding the EAB^19^. Unfortunately, due to the use of carbon-based metasurface, the composites are still unsuitable for high-temperature oxidative environment.

A metasurface that can maintain stable electrical properties and physicochemical characteristics at least at 1000 °C is still needed. For this purpose, resistive materials with low temperature coefficients of resistance (TCR, defined as *α* = (Δ*R*/*R*_*0*_)/Δ*T*, where Δ*R*, *R*_*0*_, and Δ*T* represent resistance change, initial resistance and temperature change, respectively), as well as high-temperature resistance, oxidation resistance, and tunable resistivity, must be developed to design temperature-insensitive MAMSs. At elevated temperatures, the carrier mobility in resistive materials drastically changes owing to intense lattice vibrations. Hence, single-component resistive materials generally exhibit a large TCR at high temperatures owing to the lack of mechanisms to control the carrier mobility. A low-TCR characteristic can be obtained by using materials with opposite TCR^20^. However, this strategy usually involves metals or carbon materials, which cannot work in high-temperature environments. The tunneling effect provides an opportunity and a methodology for controlling the carrier mobility. The carrier mobility during tunneling is dominated by barrier height, effectively circumventing mechanisms, such as temperature-dependent lattice vibrations. Ruthenium oxide (RuO_2_) is a widely used resistive material in electronics, featuring metallic-like conduction band electrons while exhibiting superior chemical stability and temperature resistance compared to metals. Meanwhile, RuO_2_ also exhibits a broadly tunable resistivity. Developing RuO_2_ as resistive materials for MAMSs based on the tunneling effect is an unprecedented idea, which has never been reported before. Furthermore, a multifunctional integrated composite designed using such metasurfaces and functionalized dielectric spacer layer would be a very compelling concept.

**Fig. 1:**
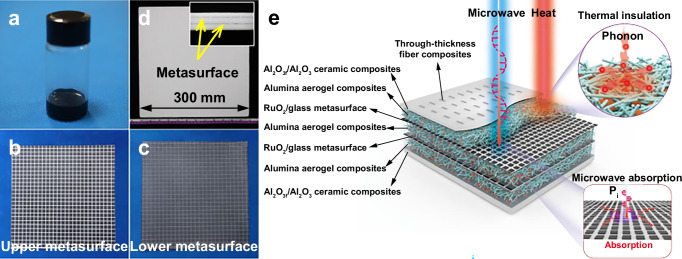
Design concept and photographs of metasurfaces and MTL integrated composites. Photographs of the **a** RuO_2_/glass resistive paste, **b** upper metasurface, **c** lower metasurface, and **d** MTL integrated composites. Inset of (**d**): side view of the MTL integrated composites. **e** Structural and functional schematic of the MTL integrated composites. The composites integrate microwave absorption, thermal insulation, and load-bearing functions through seven functional layers.

Herein, a RuO_2_/glass resistive material (Fig. 1a) with ultra-low TCR is developed based on tunneling effect, thereby designing a MAMS with temperature-insensitive electrical properties (Fig. 1b, c). On the basis of these materials, multifunctional (microwave-absorbing, thermally insulating, and load-bearing (MTL)) integrated composites with fiber-reinforced alumina aerogel and Al_2_O_3f_/Al_2_O_3_ ceramic composites as the dielectric spacer layer are proposed (Fig. 1d). According to the equivalent circuit model and EM simulation optimization, the architecture of the MTL integrated composites is designed (Fig. 1e). The outer Al_2_O_3f_/Al_2_O_3_ ceramic composites are responsible for heat protection and uniform load transfer. The alumina aerogel composites provide a thermal insulation function, and their low dielectric characteristics are crucial for broadening the absorption bandwidth and enhancing the absorption efficiency. To enhance the delamination resistance of the MTL integrated composites, the metasurfaces, aerogel composites and ceramic composites are reinforced by through-thickness stitched fibers. The MTL integrated composites show ultra-robust microwave-absorbing properties that the reflectivity can maintain <−10 dB at 2~12 GHz covering 25 °C~1000 °C and show no degradation after 50 thermal shock cycles from 25 °C to 1000 °C. The reflectivity also exhibits insensitivity to polarization and excellent stability to the incidence angle (±45°). Moreover, the MTL integrated composites combine remarkable thermal insulation properties, high load-bearing capacity, and low density (0.54 g/cm^3^). The RuO_2_/glass metasurface developed in this paper fills a gap in the field of high-temperature MAMSs. The structural idea of multifunctional integrated composites also provides guidance for the design of materials for use in extreme environments with coupled temperature, stress, and EM fields.

## Results

### Design of MTL integrated composites

**Fig. 2:**
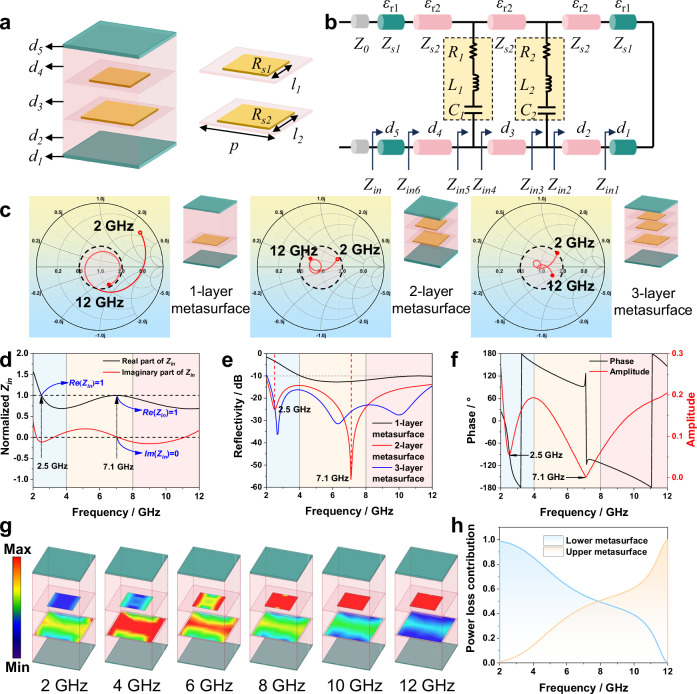
Design of MTL integrated composites. **a** Schematic diagram of the unit-cell structure. **b** Equivalent circuit diagram for MTL integrated composites. **c** Smith charts (the hollow circle end represents 2 GHz, whereas the solid end represents 12 GHz) and **e** simulated reflectivity curves of MTL integrated composites containing one-layer, two-layer, and three-layer metasurfaces. **d** Real part and imaginary part of normalized Z_in_ of the MTL integrated composites. **f** Phase and amplitude of microwave in the MTL integrated composites. **g** Distributions of the current loss densities of MTL integrated composites at 2, 4, 6, 8, 10, and 12 GHz. **h** Frequency-dependent power loss contribution of the dual-metasurface.

To develop microwave-absorbing materials with a broadband absorption ability up to 1000 °C, we first aimed to determine the optimal structural design by simulating the impedance and reflectivity of MTL integrated composites with one-, two-, and three-layer metasurfaces under the same composite thickness. As an example, the unit-cell structure and equivalent circuit of the composite with two-layer metasurface are presented (Fig. 2a, b). The periodic cell was designed as a stacked structure of three layers of alumina aerogel composites, two layers of Al_2_O_3f_/Al_2_O_3_ ceramic composites, and two layers of metasurfaces (Fig. 2a). The whole periodic cell was defined by five thickness parameters (thicknesses of the alumina aerogel composites and Al_2_O_3f_/Al_2_O_3_ ceramic composites), three cell size parameters (upper cell edge length, lower cell edge length, and periodic cell edge length), and two sheet resistivity parameters (upper cell sheet resistivity and lower cell sheet resistivity). On the basis of the equivalent circuit method and microwave network theory, the square periodic resistive patch was equated to a series circuit of resistance, capacitance, and inductance, whereas the alumina aerogel composites and Al_2_O_3f_/Al_2_O_3_ ceramic composites were equated to two transmission line models with different characteristic impedances (Fig. 2b). As input for simulation, the dielectric constants and loss tangents of the alumina aerogel composites and the Al_2_O_3f_/Al_2_O_3_ ceramic composites were measured using the waveguide method (Supplementary Fig. 1). According to the circuit model, the metasurface impedance can be expressed as:

1 $${Z}_{n}={R}_{n}+j \left(\omega {L}_{n}-\frac{1}{\omega {C}_{n}}\right)$$

where Z_n_, R_n_, L_n_, and C_n_ (*n* = 1, 2. 1 and 2 represent the top and lower metasurfaces, respectively) represent the equivalent impedance, real part of the impedance, equivalent inductance, and equivalent capacitance of the metasurface, *j* represents the imaginary unit, and *ω* represents the angular frequency.

The expression for the input impedance of the terminated short-circuited alumina dielectric spacer layer is given by:

2 $$Z {\scriptstyle{in}1}=j{Z}_{0}\sqrt{\frac{1}{{\varepsilon }_{r1}}}\,\tan \left(\frac{\omega }{c}\sqrt{{\varepsilon }_{r1}}{d}_{1}\right)$$

where Z_in1_, *ε*_*r1*_, and d_1_ represent the input impedance, dielectric constant, and thickness of the lower-layer Al_2_O_3f_/Al_2_O_3_ ceramic composites, Z_0_ is the wave impedance of free space (Z_0_ ≈ 377 Ω), and *c* is the speed of light in vacuum.

Furthermore, the expression for the input impedance of the composites with two-layer metasurface can be obtained via iterative step-by-step derivation as follows:

3 $$Z{\scriptstyle{in}}=Z{\scriptstyle{s1}}\frac{Z{\scriptstyle{in6}}+jZ{\scriptstyle{s1}} \, \tan (\frac{\omega }{c}\sqrt{{\varepsilon }_{r1}}{d}_{5})}{Z{\scriptstyle{s1}}+jZ{\scriptstyle{in6}} \, \tan (\frac{\omega }{c}\sqrt{{\varepsilon }_{r1}}{d}_{5})}$$

where Z_in_ is the input impedance of the MTL integrated composites, Z_in6_ is the input impedance of the MTL integrated composites without top layer Al_2_O_3f_/Al_2_O_3_ ceramic composites, Z_s1_ is the wave impedance of the Al_2_O_3f_/Al_2_O_3_ ceramic composites, and d_5_ is the thickness of the top-layer Al_2_O_3f_/Al_2_O_3_ ceramic composites.

The detailed expression and derivation of Eq. (3) are shown in supplementary file. Next, the thicknesses, cell size, and sheet resistivity were globally optimized for the most commonly used S-, C-, and X-bands using numerical calculations, with the EAB as the objective function. Finally, the structural parameters (Supplementary Fig. 2) of the MTL integrated composites were determined. It is worth noting that the EAB of this microwave-absorbing structure exhibits high robustness to these structural parameters (Supplementary Fig. 3), implying that fabrication tolerances are allowed.

The Smith charts for the three composites were calculated to visually assess the impedance matching (Fig. 2c). In the Smith chart, the impedance curve preferably enters the ideal matching region (pink dashed circle in Fig. 2c) and approaches the ideal matching point (central point in Fig. 2c). Most of the impedance traces of the composites with one-layer metasurfaces do not enter the ideal matching region, indicating poor impedance matching at low frequencies. The impedance traces of the other two composites all enter the ideal matching region, demonstrating their excellent impedance matching and absorption ability over the entire target frequency band. Specifically, the impedance trace of the dual-layer microwave-absorbing structure passes through the ideal matching point, which is further confirmed by its normalized input impedance with a real part of 1 and an imaginary part of 0 at 7.1 GHz (Fig. 2d). According to the simulated reflectivity curves (Fig. 2e), the composites with two-layer metasurfaces achieve a microwave absorption performance of < −10 dB at 2~12 GHz, whereas the composites with three-layer metasurfaces do not exhibit a significant reduction in the reflectivity. Additionally, the arrangement of three-layer metasurfaces increases the complexity of the integrated structure and preparation process. Therefore, setting two-layer metasurfaces within the integrated composites represents the optimal strategy. In this strategy, the resonance peaks in S-band (2.5 GHz) and C-band (7.1 GHz) ensure the broadband absorption ability of the integrated composites (Fig. 2e). The formation of the dual-resonance is primarily due to destructive interference. The phase reversal and zero amplitude at 7.1 GHz (Fig. 2f) provide clear evidence of destructive interference of EM waves. Then, the EM energy is absorbed by the metasurfaces, which significantly reduces the EM wave reflectivity. The amplitude minimum at 2.5 GHz (Fig. 2f) also indicates strong interference, corresponding to the resonance peak in S-band.

According to the simulated current loss densities of the unit structure at different frequencies (Fig. 2g), interestingly, the microwave absorption in S-band mainly originates from the EM resonance of the lower metasurface, whereas the microwave absorption in X-band is attributed to the EM resonance of the upper metasurface. Naturally, the microwave loss in C-band is contributed by both the upper and lower metasurfaces. The frequency-dependent loss contribution curves (Fig. 2h) of the dual-metasurface further confirm this EM response mechanism. The as-observed EM response can be attributed to the different impedance matching characteristics of the two metasurfaces in different frequency bands. To qualitatively clarify this EM response mechanism, the frequency-dependent curves of Z_in1_ to Z_in6_ (Supplementary Fig. 4) are provided, where Z_in3_ and Z_in5_ correspond to the impedance characteristics of the lower and upper metasurfaces, respectively. In S-band, the upper metasurface shows wave-transparent characteristics without microwave loss (Fig. 2g), whereas the lower metasurface achieves better impedance matching, resulting in perfect EM wave absorption. In X-band, the real part of Z_in3_ approaches zero, while the real part of Z_in5_ approaches 1 (Supplementary Fig. 4). This results in the lower metasurface exhibiting EM reflective behavior, while the upper metasurface achieves excellent impedance matching, thus enabling effective microwave absorption. For the C-band, the transmission, reflection and absorption characteristics for EM wave of the two metasurfaces lie between those in the above two cases. In brief, a near-ideal EM response was realized through the rational design of structural parameters.

### Electrical properties of the periodic resistive patch and preparation of MTL integrated composites

**Fig. 3:**
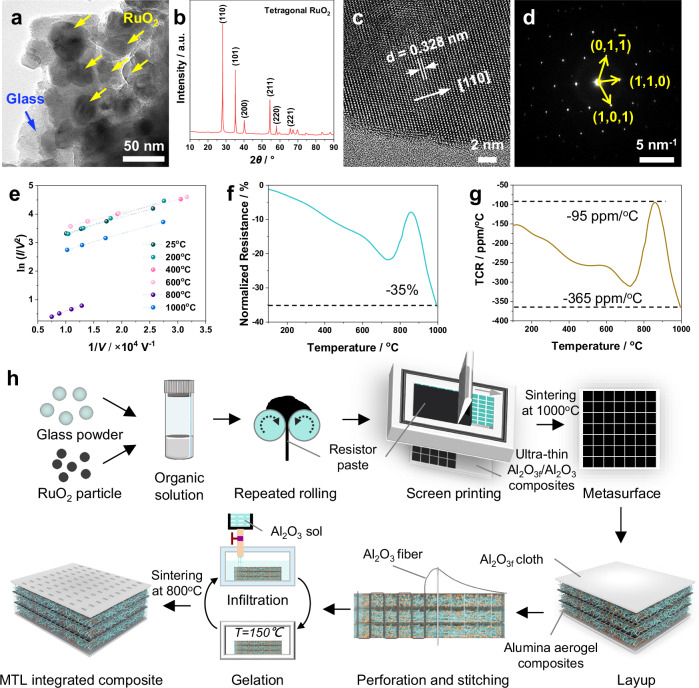
Electrical properties, microstructure, and conductive mechanism of the RuO_2_/glass resistive patch and preparation flowchart of MTL integrated composites. **a** Low-magnification TEM image and **b** XRD pattern of the RuO_2_/glass resistive patch. **c** High-resolution TEM and **d** selected area electron diffraction images of RuO_2_ nanoparticles in the resistive patch. **e** Fowler-Nordheim plots of RuO_2_/glass resistive patch at different temperatures. **f** Normalized resistance and **g** TCR of the RuO_2_/glass resistive patch. **h** Schematic illustration of the preparation of MTL integrated composites.

The key to achieving the as-designed microwave-absorbing structure and performance lies in preparing a resistive material with low TCR. Therefore, we first investigated the high-temperature electrical properties of RuO_2_/glass resistive material. The sheet resistivity of RuO_2_/glass resistive patches can be easily controlled by adjusting the RuO_2_ content, and their electrical behavior follows percolation theory (Supplementary Fig. 5). Through steps, such as mixing, rolling, and sintering, the RuO_2_/glass resistive materials with suitable resistivity were fabricated. RuO_2_ nanoparticles were uniformly distributed in glass matrix (Fig. 3a and Supplementary Fig. 6). In the X-ray diffraction (XRD) pattern (Fig. 3b), only highly crystalline RuO_2_ was detected, whereas the glass matrix was not found due to its low crystallinity, although it begins to crystallize from ~850 °C (Supplementary Fig. 7). The streaks with a distance of ~0.328 nm in the high-resolution transmission electron microscopy (TEM) image (Fig. 3c) correspond to the (110) crystal plane of tetragonal RuO_2_, and the XRD pattern (Fig. 3b) also proves that RuO_2_ belongs to tetragonal crystal system. The well-defined array of diffraction spots in Fig. 3d reveals the single-crystal structure of RuO_2_. According to the microstructure of the resistive patch, RuO_2_ nanoparticles can form direct or indirect contact modes that can be equated to several tiny conductive chains in series or parallel. For the direct contact mode, electrons can be directly transported along the conductive chain. For the indirect contact mode, electrons cannot freely travel between the conductive particles, and this hindering effect leads to the so-called barrier resistance. According to the tunneling barrier model (Supplementary Fig. 8a), when the barrier is narrow, electrons can be transported through the barrier via their wave nature. Therefore, the resistance of the RuO_2_/glass resistive patch is mainly composed of the barrier resistance (R_b_(T)) and the intrinsic resistance of RuO_2_ (R_m_(T)). The corresponding expressions are as follows:

4 $${R}_{b}(T)=\frac{1}{2}{R}_{b0}\cdot \left(\frac{\sin (aT)}{aT}\right) \cdot \left[1+\exp \left(\frac{E}{kT}\right)\right]$$

5 $${R}_{m}(T)={R}_{m0}\cdot (1+bT)$$

where *a* is related to the barrier height, *E* is the activation energy, *k* is Boltzmann’s constant, *T* is the absolute temperature, *b* is the intrinsic TCR of the conductive particles (for RuO_2_, *b* > 0), R_b0_ is the barrier transmission coefficient, and R_m0_ is the resistance of the conductive particles at absolute zero. The temperature-dependent current–voltage (I-V) characteristics (Supplementary Fig. 8b) and the linear Fowler-Nordheim (F-N) plot (ln(I/V^2^) versus 1/V) (Fig. 3e) confirm that the F-N tunneling dominates the electron transport mechanisms of the resistive patch from room temperature to 1000 °C. According to Eqs. (4, 5), R_b_(T) has negative resistance‒temperature characteristics, whereas R_m_(T) has positive resistance‒temperature characteristics. Thus, on one hand, the tunneling mechanism is insensitive to temperature; on the other hand, R_b_(T) and R_m_(T) exhibit opposite TCR. Both factors suggest that the RuO_2_/glass may show a low TCR.

To verify the high-temperature electrical properties of the RuO_2_/glass resistive patch, the normalized resistance and TCR at 25~1000 °C were tested using the high-temperature four-probe method (Supplementary Fig. 9). Although the normalized resistance of the RuO_2_/glass resistive patch increases at 800 °C–900 °C (may be due to the phase transition of the glass matrix (Supplementary Fig. 10)), it still has negative resistance‒temperature characteristics from 25 °C–1000 °C, with a TCR of −95 to −365 ppm/°C (Fig. 3f, g). Compared with other common resistive materials, such as noble metals (Au, Ag, Cu, and Pt), base-metal silicides (MoSi_2_), base-metal oxides (MoO_2_), and perovskites (LaSrCoO_3_ and LaSrMnO_3_)^17^ (Supplementary Fig. 11), the RuO_2_/glass resistive material exhibits an exceptionally ultra-low TCR, even by an order of magnitude. Furthermore, the resistive patch was maintained at 1000 °C for 10 h, with its sheet resistivity increasing by no more than 16% compared to the initial value at 1000 °C (Supplementary Fig. 12). This further confirms the stability of its high-temperature electrical performance. The ultra-low TCR ensures minimal variation in the electrical properties of the periodic resistive patch even at 1000 °C, which is the most important condition for enhancing the robustness of the microwave-absorbing performance of the MAMS under extreme-temperature environments.

To achieve the proposed microwave-absorbing structure and parameters, we developed a combination process that includes four main steps (Fig. 3h): 1) preparing the RuO_2_/glass resistor paste with viscosity of 100–300 Pa·s (Supplementary Fig. 13) to facilitate screen printing. 2) Screen printing the resistor paste on an ultra-thin Al_2_O_3f_/Al_2_O_3_ composite to obtain the metasurfaces. Notably, the RuO_2_/glass metasurface exhibits excellent mechanical flexibility with its sheet resistivity remaining nearly unchanged before and after bending (Supplementary Fig. 14). 3) Stitching the surface fabric, metasurfaces and alumina aerogel composites. The aerogel composites were hydrophobized (Supplementary Fig. 15) to protect the pore structure (Supplementary Fig. 16) from destroying during the subsequent infiltration of alumina sol that is harmful to their low dielectric characteristic and heat insulation performance. 4) Densifying the surface fabric and stitch hole, then the MTL integrated composites with a low density of 0.54 g/cm^3^ can be obtained. The above process skillfully integrates materials with different functions into a unified architectural system, markedly enhancing both the structural integrity and delamination resistance while ensuring that their respective properties remain uncompromised during the fabrication processes.

### Microwave absorption properties of MTL integrated composites

**Fig. 4:**
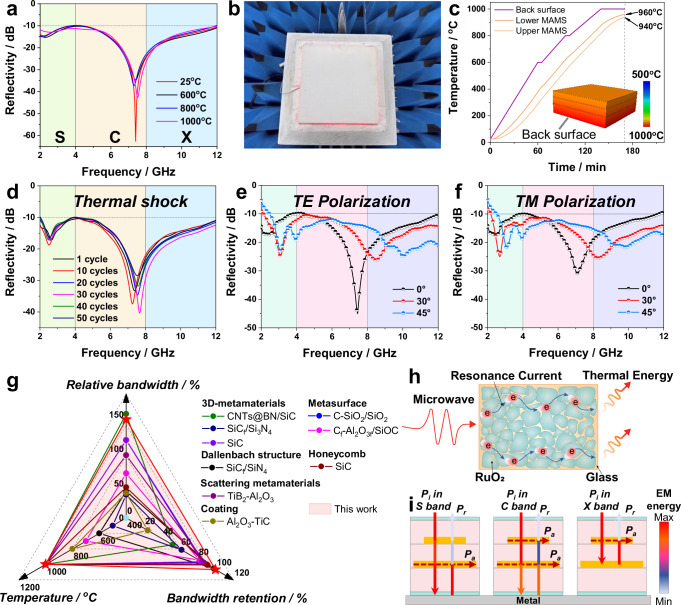
Microwave absorption performance and mechanism of MTL integrated composites. **a** Experimental reflectivity of MTL integrated composites from 25 °C to 1000 °C. **b** Photograph of the MTL integrated composite test sample. **c** Temperature variation curve and distribution in the thickness direction during high-temperature reflectivity testing obtained from finite element simulations. **d** Experimental reflectivity of MTL integrated composites after the first to 50th thermal shock cycles. **e**,**f** Experimental reflectivity of MTL integrated composites at incidence angles of 0°, 30°, and 45° measured for both TE polarization and TM polarization. **g** Comparison of the relative bandwidths, test temperatures, and bandwidth retentions between MTL integrated composites and other types of microwave-absorbing materials^18,19,22–24,35–43^. **h**,**i** Schematic illustration of the microwave absorption mechanisms for MTL integrated composites.

To evaluate the temperature robustness of the microwave-absorbing performance of the MTL integrated composites, a sample with a standard size of 300 × 300 mm was prepared according to the optimal structural parameters to test the reflectivity using the Naval Research Laboratory (NRL) arch method (Fig. 4b and Supplementary Fig. 17). From room temperature to 1000 °C, the reflectivity of the MTL integrated composites at 2–12 GHz is always below −10 dB, and the reflectivity curves highly overlap (Fig. 4a), indicating that their microwave absorption properties are very robust to temperature. All the reflectivity curves (Fig. 4a) have two resonance peaks, which agrees with the simulation results. Also, the EAB of MTL integrated composites was successfully expanded toward the S-band, which has not been achieved in previous metamaterials based on non-magnetic materials (e.g., C^21^, SiC^22^, SiOC^23^, TiB_2_^24^). Additionally, although the aerogel composite can block heat transfer, the two metasurfaces still reach high temperatures at slow heating rates (~5.8 °C/min), at prolonged heating times (~3 h) and in a closed near-adiabatic test environment. Finite element simulations revealed that the upper and lower metasurfaces can reach 940 °C and 960 °C, respectively (Fig. 4c).

The MTL integrated composites show a relative bandwidth of 143% at 1000 °C, reaching 100% bandwidth retention relative to the room-temperature bandwidth (Fig. 4a). The relative bandwidth was calculated according to the following equation:

6 $$Relative\,bandwidth=\frac{( \, {f}_{2}-{f}_{1})}{{f}_{0}}\times 100\%$$

where f_0_, f_1_, and f_2_ are the center-, low-, and high-frequency points of the band below −10 dB, respectively. The relative bandwidths, test temperatures, and bandwidth retentions of the MTL integrated composites were comprehensively compared with those of other types of high-temperature microwave-absorbing materials reported in the literature (Fig. 4g). The detailed data are summarized in Supplementary Table 3. Compared with other types of microwave-absorbing materials, such as 3D metamaterials, honeycombs, coatings, Dallenbach structures, and scattering metamaterials, the MTL integrated composites distinguish themselves prominently due to their excellent temperature robustness, high-temperature resistance, and broadband characteristics (Fig. 4g, and Supplementary Table 3). Although the target frequency bands of these materials differ, the MTL integrated composites show the best temperature robustness in terms of their microwave-absorbing properties. Moreover, compared with the most common silicon-, carbon- or oxide-based high-temperature microwave-absorbing materials, the RuO_2_-based MTL integrated composites are currently the only materials to our knowledge that simultaneously show perfect absorption in the S-band (Supplementary Table 3).

In complex coupled multi-physical field environments, the temperature is often rapidly changing, and EM waves are often randomly polarized and obliquely incident, which places higher demands on the extreme environmental adaptability of materials. Specifically, the microwave-absorbing performance of materials must also be robust to thermal shocks, polarization, and the angle of incidence. Thermal shock tests were performed on the MTL integrated composites from 25 °C to 1000 °C using a muffle furnace (Supplementary Fig. 18). During 50 thermal shock cycles, their EABs show extraordinary stability, and the positions of their resonance peaks are almost invariable (Fig. 4d). Their appearance also never shows any visible deformation or cracks even after 50 thermal shock cycles (Supplementary Fig. 19). In transverse electric (TE) polarization mode, the EAB of the MTL integrated composites at 30° and 45° only slightly decays to 9.85 GHz (2.15–12 GHz) and 9.5 GHz (2.5–12 GHz), respectively (Fig. 4e). As a simple plate-type structure, i.e., in the absence of slopes, the MTL integrated composites show superior angle adaptability. Owing to the isotropy of the metasurface and substrate, the trend of the reflectivity curves under transverse magnetic (TM) polarization is in better agreement with that under TE polarization (Fig. 4f), indicating the polarization-insensitive performance of the MTL integrated composites. In all the cases, the EAB attenuation of the MTL integrated composites does not exceed 5%. In brief, we realized impressive broadband absorption composites that are ultra-robust to temperature (25 °C–1000 °C), thermal shock (50 cycles at 25 °C–1000 °C), incidence angle (±45°), and polarization. Moreover, high-humidity conditions often present additional challenges in extreme environments, placing further demands on the long-term stability of MTL integrated composites. The humidity resistance of MTL integrated composites was evaluated (Supplementary Fig. 20) and its EAB remained unchanged after prolonged moisture exposure (95% humidity for 12 h), indicating excellent stability against humidity.

The microwave-absorbing mechanism of the MTL integrated composites is mainly resonant current loss (Fig. 4i). According to the transmission path of EM waves (marked by solid arrows) and the primary loss locations (marked by red dashed lines) (Fig. 4h), incident waves are transmitted through the alumina dielectric layer and metasurface, and then, the return wave eventually enters the periodic resistive patch and is coupled with the resonant current. EM energy in different frequency bands is absorbed by different metasurfaces, e.g., in the S-band, microwaves will form strong resonant currents on the lower metasurface.

### Thermal insulation of MTL integrated composites

**Fig. 5:**
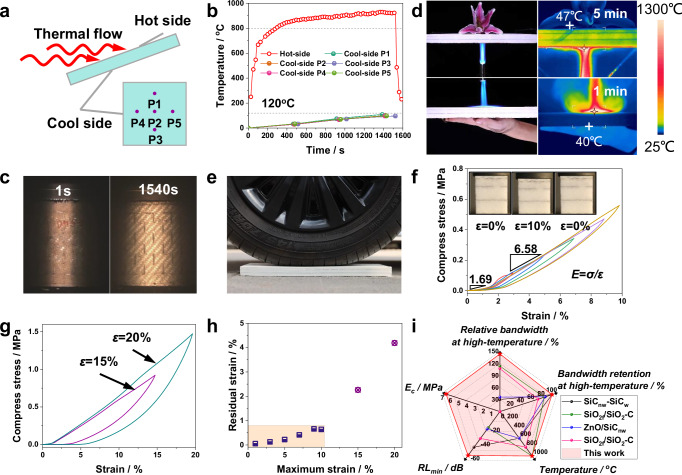
Thermal insulation and compression properties of MTL integrated composites. **a** Schematic diagram of heat flow blowing and the temperature measurement points of a sample. **b** Hot-side and back-side temperature versus time curves of MTL integrated composites in an arc wind tunnel. **c** Typical photographs of a sample in the wind tunnel. **d** Photographs and infrared images of MTL integrated composites for thermal protection of a flower and the human body under a butane blowtorch. **e** Load-bearing test for MTL integrated composites. **f** Uniaxial compression curves of MTL integrated composites with strains of 1–10%. Inset: experimental snapshots of one compression cycle. **g** Uniaxial compression curves of MTL integrated composites with strains of 15 and 20%. **h** Maximum strain‒residual strain plot for all compression loading‒unloading tests. **i** Comprehensive properties comparison of MTL integrated composites and materials with similar functions in the literature^19,31–33^.

Microwave-absorbing components operating in harsh environments are often subjected to extreme conditions, such as large temperature gradients, long-term high-temperature exposure, and rapid temperature changes. The thermal insulation function of these microwave-absorbing components is particularly crucial to protect the normal operation of internal components. According to steady-state measurements, the MTL integrated composite exhibits ultra-low thermal conductivity at room temperature (38 mW/(m·K)) and 1000 °C (143 mW/(m·K)) (Supplementary Table 4), which is comparable to state-of-the-art thermal insulation materials, such as ZAGs aerogel^25^ or *h*-BN aerogel^26^ (Supplementary Fig. 21). An arc wind tunnel test was used to assess the long-term thermal insulation capability and resistance to gas flushing and thermal shock of the MTL integrated composites. A schematic diagram of heat flow blowing and temperature test points on the back side are shown in Fig. 5a. According to Fig. 5b, even when the temperature of the hot side of the MTL integrated composites is higher than 800 °C for a long time (~1500 s), the temperature of the back side does not exceed 120 °C. Typical wind tunnel photographs of the MTL integrated composites during the test are shown in Fig. 5c and Supplementary Fig. 22. The results of the arc wind tunnel test demonstrate the excellent long-term thermal protection ability of the MTL integrated composites.

We further evaluated the practical thermal insulation performance of the MTL integrated composites. A lily flower was placed on an MTL integrated composite plate, and the bottom surface was heated using a butane flame (~1300 °C) (Fig. 5d). After heating for 5 min, the bottom temperature of the lily flower was ~47 °C, and the lily flower did not show any thermal damage (Supplementary Video 1). Additionally, we placed the MTL integrated composite plate directly on a hand and heated the top surface using a butane flame. One minute later, the temperature of the bottom surface of the composites was only ~40 °C, which is well within the tolerable range of the human body (Fig. 5d). The above results demonstrate the remarkable thermal insulation performance of the MTL integrated composites.

### Compressive mechanical properties of MTL integrated composites

The mechanical properties of the MTL integrated composites are equally important for their practical use. The MTL integrated composites can withstand crushing by a 0.8 t car and recover their shape (Fig. 5e and Supplementary Video 2), which demonstrates their remarkable load-bearing capacity. When a small region of the MTL composites is under high loading, the high-modulus Al_2_O_3f_/Al_2_O_3_ ceramic composite can uniformly transfer loads, thereby relieving the stress concentration in the aerogel composites. Next, we investigated the compressive behavior of the MTL integrated composites under quasi-static compression. In the tests, compression deformation mainly occurs in the alumina aerogel composites. When the compressive strain is ≤10% (Fig. 5f), the aerogel composites exhibit elastic deformation with negligible residual strain (<0.7%, Fig. 5h). The inset of Fig. 5f and Supplementary Video 3 show one loading‒unloading cycle at 10% strain, which demonstrates the fast compression resilience characteristics. When the compressive strain reaches 15% (Fig. 5g), the unloading curve already demonstrates a significant residual strain (~2.3%, Fig. 5h), indicating plastic deformation of the aerogel composites. On the basis of the slope in the elastic regime of the loading curve (strain = 10%) in Fig. 5f, the compressive modulus of the MTL integrated composites was estimated to be ~1.69 MPa for the low-strain regime (strain <1.5%) and ~6.58 MPa for the high-strain regime (1.5% <strain <10%). This compressive modulus is 1~2 orders of magnitude higher than those of aerogels without reinforced fibers^25,27^ or general ceramic fiber felt^28–30^. In addition to the strengthening effect of the fibers within the aerogel composites, stitching of fibers along the thickness direction can further enhance the compressive modulus. Additionally, the change in modulus was attributed to the transformation of the main load-bearing unit from the low-modulus Al_2_O_3_ matrix to the high-modulus Al_2_O_3_ fibers. Moreover, fatigue resistance under cyclic loading and the temperature dependence of mechanical properties are critical for practical applications. The compressive loading-unloading tests demonstrate the durable cyclic performance of the MTL integrated composite at 5% strain (Supplementary Fig. 23a). After 100 compression cycles, the relative height decreased by only 1.3%, while the maximum stress remained nearly constant (Supplementary Fig. 23b, c). The temperature-dependent compressive cycling tests revealed that the MTL integrated composite exhibits an increased compressive modulus, enhanced damping capacity, and reduced maximum elastic strain (Supplementary Fig. 23d, e). In brief, the MTL integrated composites can both absorb energy through small elastic deformations to maintain the robustness of the structure and provide a shape retention capacity through their high stiffness, which in turn ensures the stability of the size-dependent microwave absorption and thermal insulation properties. Figure 5i provides a comprehensive comparison of the properties of MTL integrated composites and other materials with similar functions^19,31–33^. The MTL integrated composites stand out for their unparalleled advantages in terms of high-temperature relative bandwidth (143% at 1000 °C), bandwidth retention (100%), high-temperature resistance (1000 °C), maximum absorption intensity (−63 dB), and compression modulus (6.58 MPa).

## Discussion

In summary, we present a microwave-absorbing metasurface capable of maintaining broadband absorption under extreme temperatures up to 1000 °C, along with a multifunctional composite that integrates microwave absorption, thermal insulation, and load-bearing. In previous studies, the drastic temperature-dependent variations in electrical properties of microwave-absorbing materials have not been effectively addressed, leading to widespread instability problem in microwave absorption performance. Our work resolves this issue through developing a RuO_2_/glass resistive material with ultra-low TCR based on tunneling effects, which fundamentally achieves stable electrical properties and electrical loss ability at elevated temperatures. The resulting MTL integrated composites show a broadband absorption ability of < −10 dB at 2–12 GHz without any degradation under varying temperature (25 °C–1000 °C), polarization (TE and TM), angle of incidence (±45°), or thermal shock (50 cycles at 25 °C–1000 °C). Moreover, the MTL integrated composites also exhibit excellent long-term thermal insulation performance and load-bearing ability (compression modulus of 6.58 MPa), as well as a low density (0.54 g/cm^3^). This work not only opens up a unique perspective for developing broadband microwave-absorbing materials under extreme high-temperature, but also provides important insights into the structural design of multifunctional integrated composites.

## Methods

### Materials

All the materials used in this work are listed in the supplementary file. The preparation process of the MTL integrated composites is described below.

Preparation of the organic solution: first, butylcarbinol and tributyl citrate were poured into a beaker. Then, ethyl cellulose was slowly added and stirred in a water bath at 90 °C for 2 h to completely dissolve the ethyl cellulose. Finally, Span-85 and 1,4-butyrolactone were added, with continuation of stirring at 90 °C for 2 h to obtain a clarified and transparent organic solution. In the above steps, the mass ratios of butylcarbinol, tributyl citrate, ethyl cellulose, Span-85, and 1,4-butyrolactone were 80%, 10%, 2%, 3%, and 5%, respectively.

Preparation of refined glass powder: first, glass fragments were melted at 1400 °C for 3 h in a muffle furnace. Second, the obtained glass melt was poured into deionized water for quenching and cooling to obtain glass slag. Finally, the glass slag was dispersed in acetone and ball milled for at least 4 h using a planetary ball mill to obtain refined glass powder.

Preparation of the resistor paste: first, the refined glass powder and RuO_2_ particles were mixed using a planetary mixer for at least 2 h. The mass ratio of RuO_2_ in the mixed powder was 50%. The mixed powder was subsequently poured into the organic solution at a mass ratio of 8:2. Finally, the resulting mixed paste was repeatedly rolled using a three-roll mixer to obtain the desired resistor paste, whose viscosity should be carefully controlled within the range of 100–300 Pa·s.

Preparation of metasurfaces: First, an Al_2_O_3f_/Al_2_O_3_ composite substrate with a thickness of ~0.3 mm had to be prepared. One layer of alumina fiber fabric (300 × 300 mm) was used as the preform and infiltrated with aluminum sol for ~1 h. The preform was subsequently dried at 150 °C for 2 h and sintered at 1000 °C for 1 h. This process was repeated until the weight gain was less than 0.5%, and the as-obtained ultra-thin Al_2_O_3f_/Al_2_O_3_ substrates were polished smooth. The resistor paste was then printed on the surface of the ultra-thin Al_2_O_3f_/Al_2_O_3_ substrates using a screen of 350 mesh (for the upper metasurface) and 120 mesh (for the lower metasurface). Finally, two metasurfaces could be obtained after drying at 150 °C for 30 min and sintering at 1000 °C for 10 min in air.

Preparation of superhydrophobic alumina aerogel composites: for the preparation process of the alumina aerogel composites, previous literature was referenced^34^. First, Al_2_O_3_ chopped-fiber felt was soaked in alumina sol for ~4 h. Second, the alumina sol was gelled and aged for 5 days at room temperature. Third, the fiber/gel mixtures were dried under supercritical ethanol. Fourth, the as-dried alumina aerogel composites were heated at 800 °C for 24 h to remove impurities. Finally, the alumina aerogel composites were hydrophobized using gas-phase hexamethyl disilazane as a hydrophobic agent.

Preparation of MTL integrated composites: according to the optimal size and sheet resistivity obtained via simulation, alumina aerogel composites (300 × 300 mm) with the target thickness and metasurfaces with the target sheet resistivity were prepared. Then, three layers of aerogel composites and two layers of metasurfaces were stacked in an alternating order. Next, Al_2_O_3_ fiber cloth with thicknesses of 0.5 mm and 0.2 mm was laid on the upper and lower surfaces, respectively. Finally, after all the material layers were aligned, perforations were made in the thickness direction (pore diameter ≈ 0.8 mm) at a distance of ~12 mm. The overall composites were perforated and stitched with Al_2_O_3_ fibers. After 10 cycles of infiltration and gelation (at 150 °C) of the Al_2_O_3_ sol, the composites were sintered at 800 °C for 1 h, and MTL integrated composites were obtained.

### Characterization

EM properties: to meet the EM simulation requirements, the complex permittivity of the alumina aerogel composites and Al_2_O_3f_/Al_2_O_3_ ceramic composites at 25 °C–1000 °C were measured with a vector network analyzer (VNA, N5225B, Keysight Technologies, USA) using the waveguide method. The microwave reflectivity (2–12 GHz) of the MTL integrated composites was measured using the NRL arch method. A pair of double-ridged antennas was employed, with one acting as a transmitter and the other acting as a receiver. The antennas were connected to the VNA via coaxial cables, and a test sample with a size of 300 × 300 × 18.1 mm was placed on a metal loading platform. The polarization of the incident microwaves was changed by rotating the azimuthal angle of the antenna horn. The incidence angle (0~45°) was varied by controlling the position of the antenna, which was fixed in the arch-shaped test fixture. When the reflectivity was tested at high temperatures, a metal cover with an insulated cotton interlayer was placed over the sample to create a closed near-adiabatic environment. The heating rate was 10 °C/min between 25 °C and 600 °C and 5 °C/min from 600 °C to 1000 °C. When the test temperature was reached, the sample was held for 30 min. Then, the metal cover was removed, and the reflectivity was quickly tested. The test temperatures were 600 °C, 800 °C, and 1000 °C. The thermal shock experiments of the MTL integrated composites were conducted in a muffle furnace. When the furnace temperature reached 1000 °C, the sample was directly placed in the furnace, and the sample was removed after 4 min for cooling to room temperature in air. The above thermal shock cycle was repeated 50 times. The room-temperature reflectivities of the samples were measured after the 1st, 10th, 20th, 30th, 40th, and 50th thermal shock experiments.

Thermal insulation properties: The room-temperature thermal conductivity of alumina aerogel composites was tested using a thermal conductivity tester (DRPL-III, Xiangtan Xiangyi Instrument CO., LTD., China) based on heat flow meter method with sample size of 300 × 300 × 18 mm. The thermal conductivity of alumina aerogel composites at 1000 °C was tested using a high-temperature thermal conductivity tester (DRS-III, Xiangtan Xiangyi Instrument CO., LTD., China) based on water flow plate method with sample size of Φ180 × 18 mm. The thermal insulation performance of the MTL integrated composites was examined for a sample with dimensions of 120 × 120 × 18.1 mm using an arc wind tunnel. The total heating time was 1540 s, and the hot-side temperature was greater than 900 °C. The temperature of the hot side was measured with an infrared thermometer, and that of the back side was measured with a K-type thermocouple. Infrared images of the MTL integrated composites were recorded using a Flir T1050sc infrared thermal imager with a working range of 7.5–14 μm.

Compressive properties: Quasi-static compression tests were conducted on bars with dimensions of 15 × 15 × 18.1 mm using a WDW-50 universal testing machine at a load rate of 2 mm/min. For compressive strains of 1–10% and 15–20%, 1000 N load cells and 50 kN load cells were used, respectively.

Other characterization and simulation methods are described in detail in the supplementary file.

## Supplementary information

Supplementary Information

Description of Additional Supplementary Files

Supplementary Video 1

Supplementary Video 2

Supplementary Video 3

Transparent Peer Review file

## Data Availability

The authors declare that all data supporting the findings of this study are available within the article and the Supplementary Information. Source data are provided with this paper.
