# Supplementary material for: Metasurface-based multifunctional composites with ultra-robust broadband microwave absorption up to 1000 °C
Source: Nat Commun. 2025 Nov 25;16:10427. doi: 10.1038/s41467-025-66317-3 (PMC12647858; doi:10.1038/s41467-025-66317-3)
Supplement: Supplementary file 1 — Supplementary Information [file 41467_2025_66317_MOESM1_ESM.pdf]

*Supporting Information for*

# Metasurface-based multifunctional composites with ultra-robust broadband microwave absorption up to 1000 °C

Xinyuan Lv[1,†], Qiujin Gu[1,†], Shengchi Zhu[2], Xun Sun[1], Minglong Yang[1], Tao Liu[1], Yunpeng Ma[3]*, Zhenxin Cao[2]*, Haitao Liu[1]*

[1]Science and Technology on Advanced Ceramic Fibers and Composites Laboratory, College of Aerospace Science and Engineering, National University of Defense Technology, Changsha, P.R. China.

[2]State Key Laboratory of Millimeter Waves, Southeast University, Nanjing, P.R. China.

[3]School of Aeronautical Science and Engineering, BeiHang University, Beijing, P.R. China.

*Corresponding author E-mail address: liuhaitao07@nudt.edu.cn (HT. Liu); caozx@seu.edu.cn (ZX. Cao); myp@buaa.edu.cn (YP. Ma).

[†]These authors contributed equally: Xinyuan Lv, Qiujin Gu.

This PDF file includes:

Supplementary Note 1-5;

Supplementary Figure 1-23;

Supplementary Table 1-4;

**Supplementary Note 1. Materials:**

Butylcarbinol, tributyl citrate, ethyl cellulose, sorbitan trioleate (Span-85, $C_{60}H_{108}O_8$, $M_w$=957), 1 ,4-butyrolactone, hexamethyl disilazane, and acetone were purchased from Shanghai Macklin Biochemical Technology Co., Ltd., China. $RuO_2$ particles with size of $d_{10}/d_{50}/d_{90}$ = 0.1 μm/1.43 μm/19.86 μm were supplied from Sino-Platinum Metals Co., Ltd. China. Glass slags were self-made in lab and their basic properties were provided in Supplementary Table 1. Alumina sol was purchased from Qingdao Hi-Tech Moulds & Plastics Technology Co., Ltd. China. $Al_2O_3$ chopped-fiber felt were purchased from Wei Ye Crystal Fiber Co., Ltd., China. $Al_2O_3$ fiber and $Al_2O_3$ fiber cloth was purchased from Dongheng Guoxian New Materials Co., Ltd.

Supplementary Table 1 The basic properties of the glass

| Density (g/cm$^3$) | CTE (K$^{-1}$) | $d_{10}/d_{50}/d_{90}$ (μm) | Element contents (wt.%) | Phase composition |
|---|---|---|---|---|
| 2.53 | $8 \times 10^{-6}$ | 1.61/5.51/16.11 | Al:10.57<br>K:4.904<br>Zn:4.755<br>Ba:0.12<br>Na:1.169<br>Si:19.31<br>O:59.172 | K-Na feldspar, Aluminosilicate, Quartz |

**Supplementary Note 2. Electromagnetic simulation:**

Electromagnetic simulation of the designed microwave-absorbing structure is performed in High Frequency Structure Simulator (HFSS) software combined with Advanced Design System (ADS) software to evaluate its reflective properties, impedance characteristic, current loss densities and so on. Specifically, the microwave-

absorbing structure is placed horizontally in x-y plane and the perfect electric conductor, the lower $Al_2O_{3f}/Al_2O_3$ ceramic composite layer, the lower aerogel composites layer, the lower metasurface layer, the intermediate aerogel composites layer, the upper metasurface layer, the upper aerogel composites layer, and the upper $Al_2O_{3f}/Al_2O_3$ ceramic composite layer are distributed in a positive direction along z-axis, from bottom to top, respectively. The cells of the two-layer metasurface are characterized by square resistor sheets with different resistances. Electromagnetic parameters of aerogels composites and ceramic composites were introduced into the dielectric layer model. Periodic boundary conditions and Flouqet excitation ports are set to simulate an infinite absorber. The line-polarized incident wave is incident on the surface of the absorber along -z direction and the electric field polarization direction is parallel to x-axis. ADS simulation is performed based on the equivalent circuit model.

**Supplementary Note 3. Heat transfer simulation:**

A 3D model of 7 layers with dimensions of 50 mm × 50 mm × 18.1 mm was created in Abaqus software. The top layer of 0.5 mm and the bottom layer of 0.2 mm correspond to $Al_2O_{3f}/Al_2O_3$ ceramic composites. The thickness of two metasurface layers was both set as 0.3 mm. The remaining positions are alumina aerogel composites. The properties of the composites are defined by the thermal, mechanical, and density parameters in Supplementary Table 2. The parameters of metasurface layer were the same to $Al_2O_{3f}/Al_2O_3$ ceramic composites because its substrate is $Al_2O_{3f}/Al_2O_3$ ceramic composites. The heating procedure was consistent with the experiment and the bottom

of the model was heated. The ambient temperature was set to 25 °C. The mesh size was 1 mm and a C3D8T heat transfer cell was used with nodes having only temperature degrees of freedom.

Supplementary Table 2 The thermal, mechanical, and density parameters of alumina aerogel composites and $Al_2O_{3f}/Al_2O_3$ ceramic composites

| Materials | Density ($g/cm^3$) | Elasticity modulus (MPa) | Poisson's ratio | Coefficient of thermal expansion ($K^{-1}$) | Specific heat (kJ/kg·K) | Thermal conductivity (W/(m·K)) | |
|---|---|---|---|---|---|---|---|
| Alumina aerogel composites | 0.38 | 6.58 | 0.09 | $6\times10^{-6}$ | 0.9 | 25℃ | 0.03 |
| | | | | | | 400℃ | 0.034 |
| | | | | | | 500℃ | 0.037 |
| | | | | | | 600℃ | 0.041 |
| | | | | | | 700℃ | 0.045 |
| | | | | | | 800℃ | 0.048 |
| | | | | | | 900℃ | 0.054 |
| | | | | | | 1000℃ | 0.064 |
| $Al_2O_{3f}/Al_2O_3$ ceramic composites | 2.1 | $3\times10^4$ | 0.09 | $6.45\times10^{-6}$ | 1.96 | 25℃ | 2.17 |
| | | | | | | 400℃ | 1.24 |
| | | | | | | 500℃ | 1.79 |
| | | | | | | 600℃ | 1.67 |
| | | | | | | 800℃ | 1.62 |
| | | | | | | 1000℃ | 1.80 |

**Supplementary Note 4. Materials Characterizations:**

Structural characterization: In order to observe the morphology of the resistive film, the resistor paste was printed on an alumina substrate and sintered. The alumina substrate was cut into rectangle with size of 20 mm × 10 mm using low-speed cutter and cold inlaid with acrylic resin. The sample was polished carefully to observe the cross-section morphology of resistive film using field emission scanning electron microscopy (SEM, Tescan Clara GMH, TESCAN, Czech Republic). The backscattered

electron (BSE) morphology and elemental mapping of the samples were examined using a BSE detector and an energy-dispersive spectroscopy (EDS) detector equipped on the SEM. Double Cs corrector transmission electron microscope (TEM, Themis Z, FEI, America) was used to observe the microstructure of the resistive film. The TEM samples were prepared by directly sintering the resistor paste and then grinding it into fine particles. X-ray diffractometer (XRD, X' Pert Pro, Philips, Netherlands) using Cu $K_\alpha$ ($\lambda$ = 1.54 Å) radiation was used to analyze the phase composition of the resistive film. Simultaneous thermal analysis (STA 449C, Netzsch Group, Germany) was employed to characterize the differential scanning calorimetry (DSC) of the glass powder from room temperature to 1100 °C at a heating rate of 5 °C/min. The contact angle of alumina aerogel composites with water was measured using an optical tensiometer (Theta Flex, Biolin Scientific, Sweden) with sample dimensions of 20 mm × 20 mm × 10 mm.

Electrical property: The resistor paste was printed on $Al_2O_{3f}/Al_2O_3$ composites substrate and sintered. The sheet resistivity of $RuO_2$/glass resistive patch at 25 °C ~ 1000 °C or with different $RuO_2$ content was measured using a high-temperature four probe resistivity tester. The normalized resistivity was calculated according to the following equation:

$$R_N = \frac{\left(R_{st} - R_{s0}\right)}{R_{s0}} \times 100\% \quad (1)$$

where $R_N$, $R_t$, and $R_0$ represent normalized resistivity, sheet resistivity at test temperature, and sheet resistivity at room temperature, respectively. The TCR of the $RuO_2$/glass material was calculated according to the following equation：

$$TCR = \frac{R_N}{T_t - T_0} \tag{2}$$

where $T_t$ and $T_0$ are test temperature and room temperature.

The $RuO_2$/glass resistor paste was printed and sintered on an alumina ceramic substrate with dimensions of 15 mm × 4 mm × 1 mm. The sample was then used to tested the current-voltage characteristic at 25~1000 °C using the high-temperature four probe system.

High-temperature compressive property: High-temperature compression tests were conducted on bars with dimensions of 15 mm × 15 mm × 18.1 mm using a WDW-50 universal testing machine at a load rate of 2 mm/min. A heating chamber encloses the sample and indenter. The protruding section of the indenter outside the chamber is connected to a cooling water supply. The test is initiated after the temperature stabilizes at the target value and is maintained for 10 min.

**Supplementary Note 5. Derived formula of input impedance:**

The impedance of free space is:

$$Z_0 = 377\Omega \tag{3}$$

The impedance of ceramic composites (n=1) and aerogel composites (n=2) substrate is:

$$Z_{sn} = 377\Big/\sqrt{\varepsilon_{rn}}\,(n = 1, 2) \tag{4}$$

The phase expression is:

$$\theta_m = \begin{cases} \dfrac{\omega d_m \sqrt{\varepsilon_{r1}}}{c}, m = 1,5 \\ \dfrac{\omega d_m \sqrt{\varepsilon_{r2}}}{c}, m = 2,3,4 \end{cases} \tag{5}$$

The equivalent impedance of the two metasurfaces are:

$$Z_{L1} = R_1 + j(\omega L_1 - \frac{1}{\omega C_1}) \tag{6}$$

$$Z_{L2} = R_2 + j(\omega L_2 - \frac{1}{\omega C_2}) \tag{7}$$

The expression of $Z_{in}$ can be derived through the following process:

$$Z_{in1} = jZ_{s1}\tan(\theta_1) \tag{8}$$

$$Z_{in2} = Z_{s2}\frac{Z_{in1} + jZ_{s2}\tan(\theta_2)}{Z_{s2} + jZ_{in1}\tan(\theta_2)} \tag{9}$$

$$Z_{in3} = \frac{Z_{L1}Z_{in2}}{Z_{L1} + Z_{in2}} \tag{10}$$

$$Z_{in4} = Z_{s2}\frac{Z_{in3} + jZ_{s2}\tan(\theta_3)}{Z_{s2} + jZ_{in3}\tan(\theta_3)} \tag{11}$$

$$Z_{in5} = \frac{Z_{L2}Z_{in4}}{Z_{L2} + Z_{in4}} \tag{12}$$

$$Z_{in6} = Z_{s2}\frac{Z_{in5} + jZ_{s2}\tan(\theta_4)}{Z_{s2} + jZ_{in5}\tan(\theta_4)} \tag{13}$$

$$Z_{in} = Z_{s1}\frac{Z_{in6} + jZ_{s1}\tan(\theta_5)}{Z_{s1} + jZ_{in6}\tan(\theta_5)} \tag{14}$$

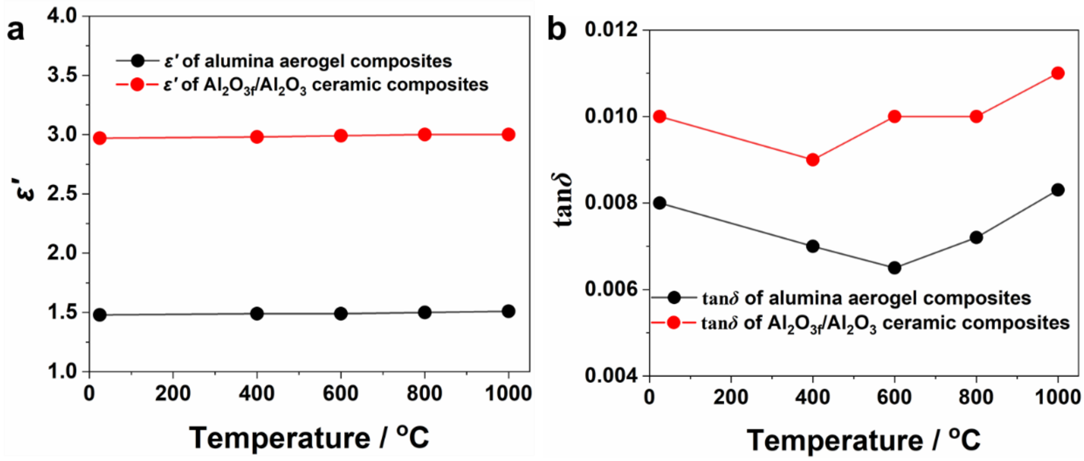


Supplementary Fig. 1 High-temperature dielectric properties of alumina aerogel composites and $Al_2O_{3f}/Al_2O_3$ ceramic composites at 10 GHz. (a) dielectric constant ($\varepsilon'$); (b) loss tangent ($\tan\delta$).

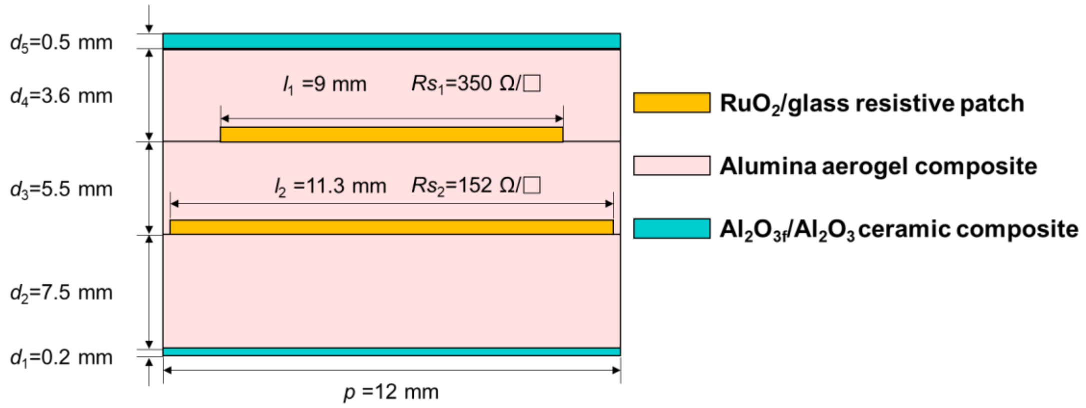


Supplementary Fig. 2 The optimal structural parameters of MTL integrated composite with dual-metasurface.

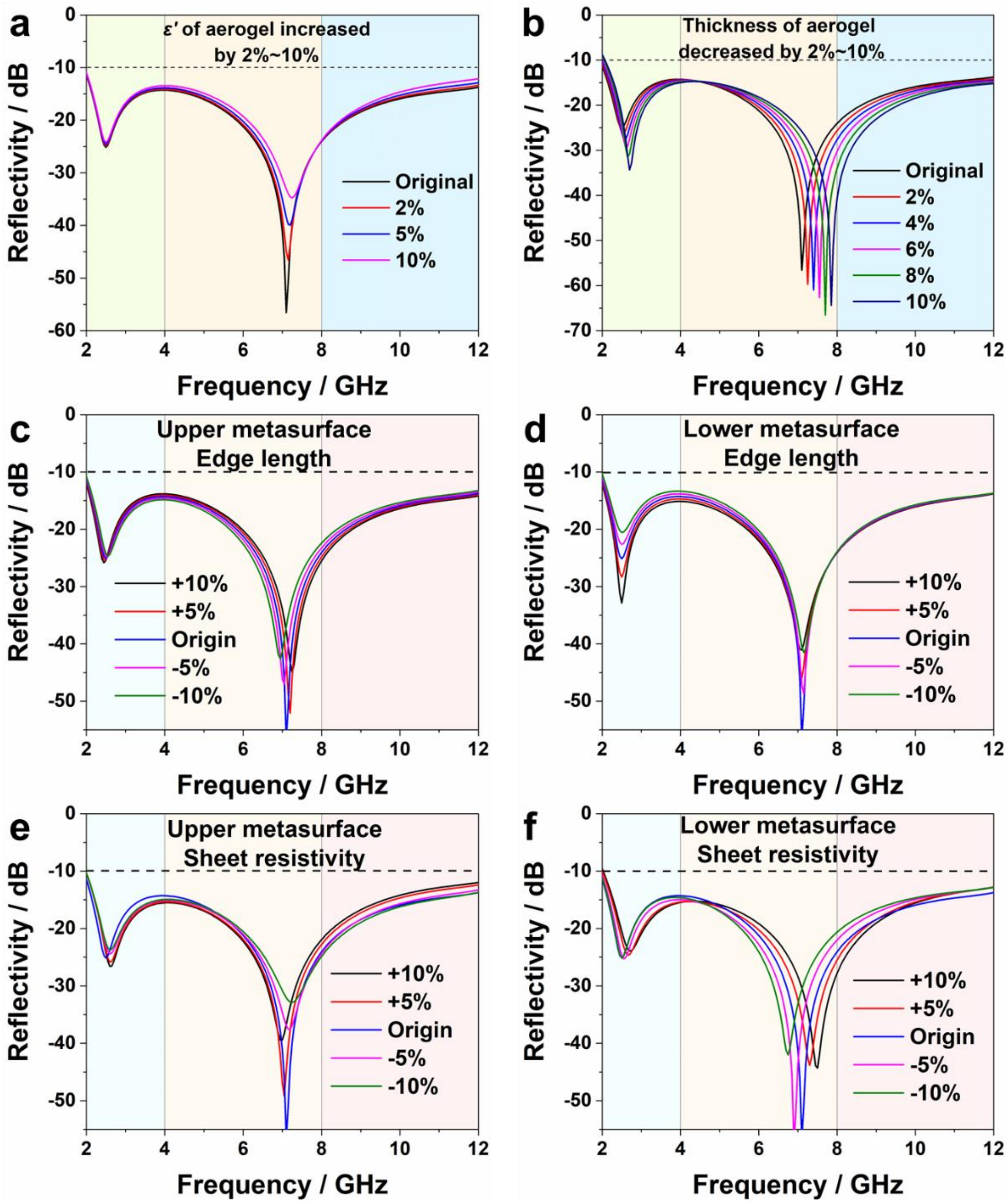


Supplementary Fig. 3 Simulated reflectivity of the MTL integrated composites under different structural parameters. (a) Different $\varepsilon'$ of aerogel composites, (b) different thickness of aerogel composites, different edge length of the (c) upper metasurface and (d) lower metasurface, different sheet resistivity of (e) upper metasurface and (f) lower metasurface.

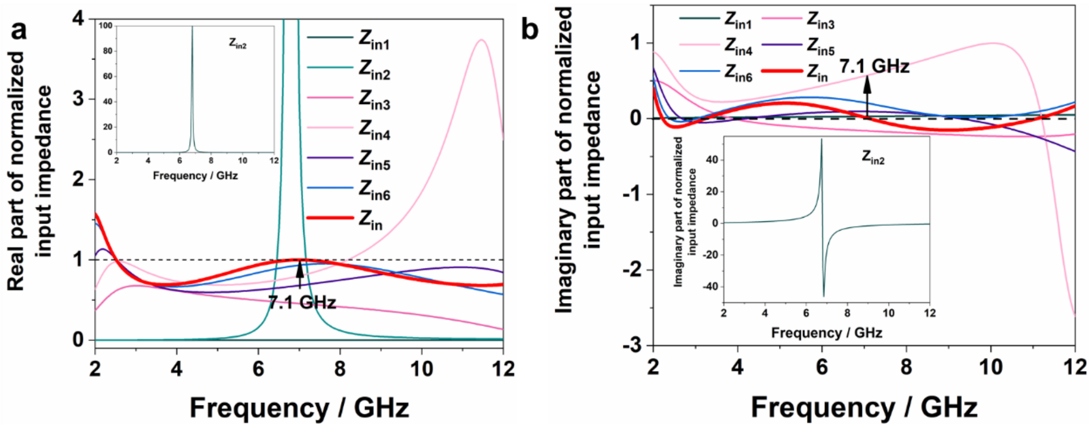


Supplementary Fig. 4 (a) Real part and (b) imaginary part of $Z_{in1}$~$Z_{in6}$ and $Z_{in}$.

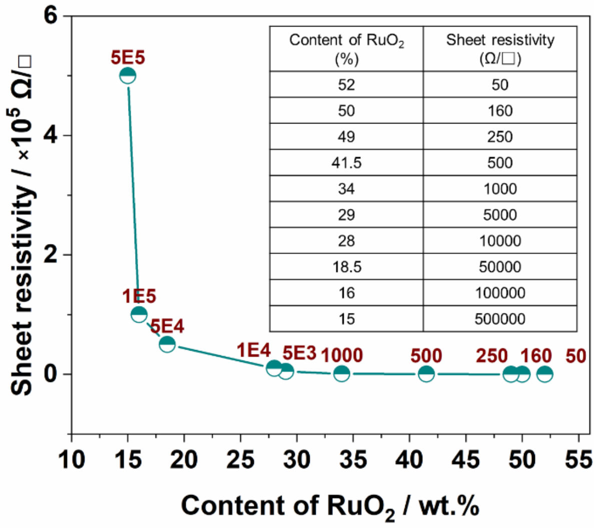


Supplementary Fig. 5 Sheet resistivity of $RuO_2$/glass resistive patches with different contents of $RuO_2$.

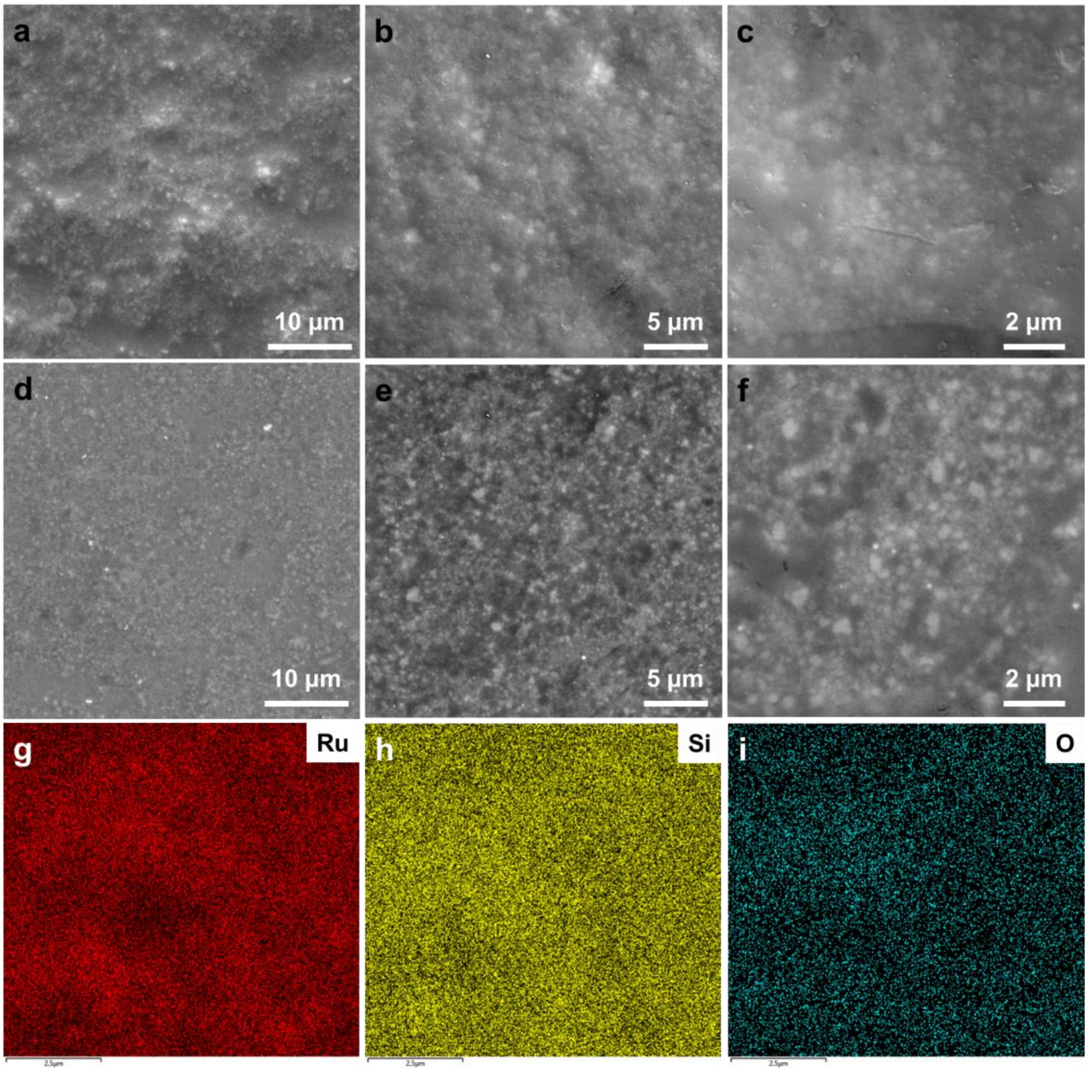


Supplementary Fig. 6 The morphology and elemental distribution of $RuO_2$/glass resistive patch. (a~c) SEM and (d~f) BSE images of the surface of the $RuO_2$/glass resistive patch at different magnifications. (g~i) EDS mappings of Ru, Si, and O elements.

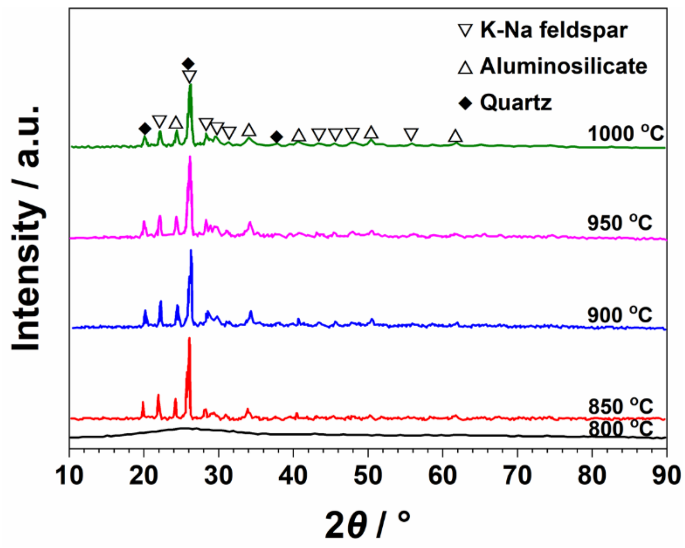


Supplementary Fig. 7 XRD patterns of the glass at different sintering temperatures.

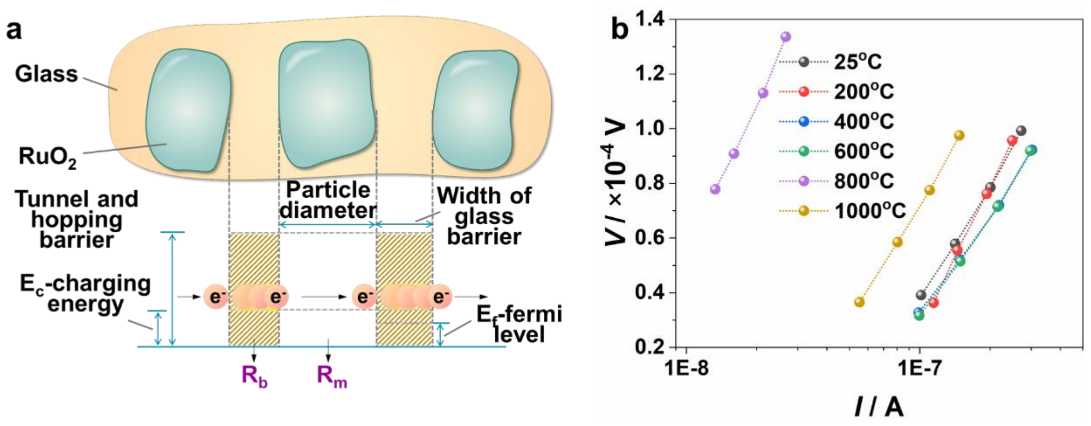


Supplementary Fig. 8 (a) Schematic diagram of the tunnel barrier effect in the $RuO_2$/glass resistive patch. (b) I-V plots of $RuO_2$/glass resistive patch at different temperatures.

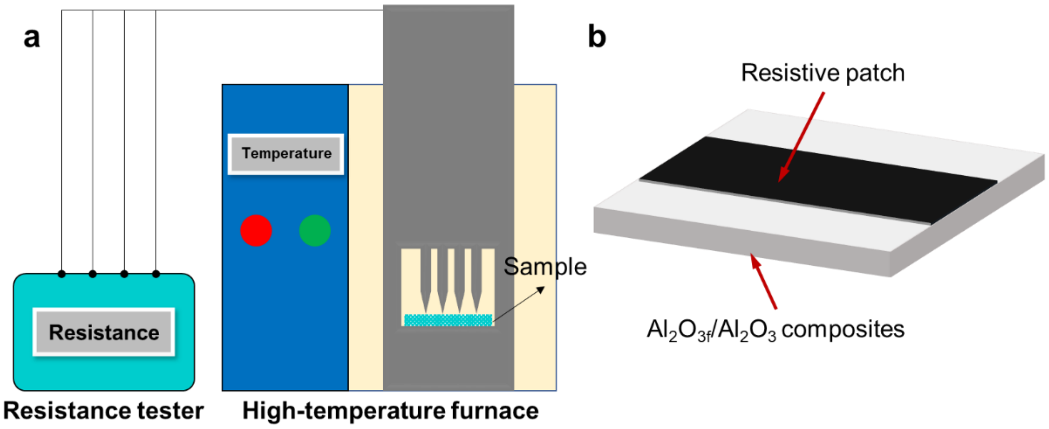


Supplementary Fig. 9 Schematic diagram of (a) high-temperature four probe resistivity tester and (b) preparation of sample.

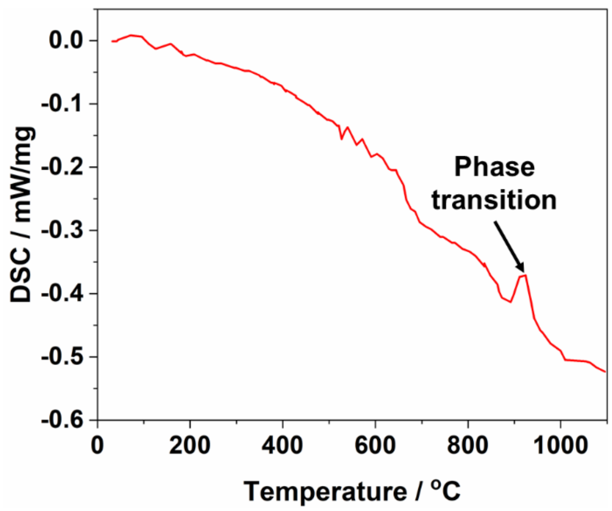


Supplementary Fig. 10 DSC curve of the glass from room temperature to 1100 °C.

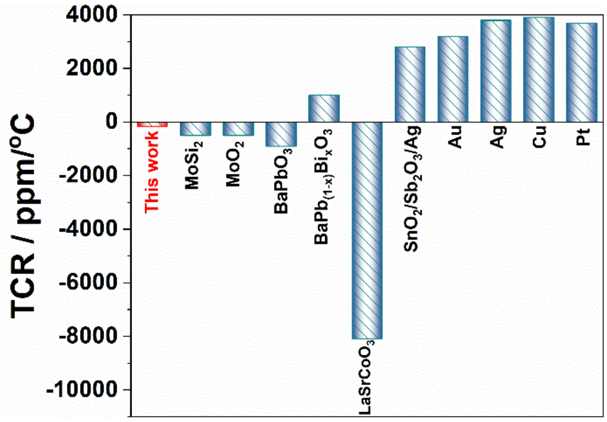


Supplementary Fig. 11 Comparison of room-temperature TCR of $RuO_2$/glass with other common resistive materials.

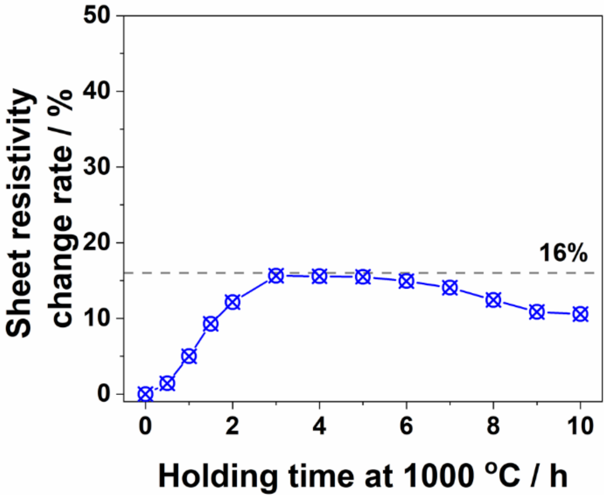


Supplementary Fig. 12 Sheet resistivity change rate of the $RuO_2$/glass resistive patch at different holding times at 1000 °C.

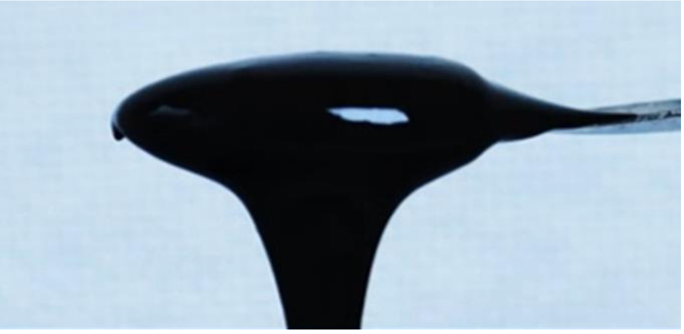

Supplementary Fig. 13 Photograph of the $RuO_2$/glass resistor paste.

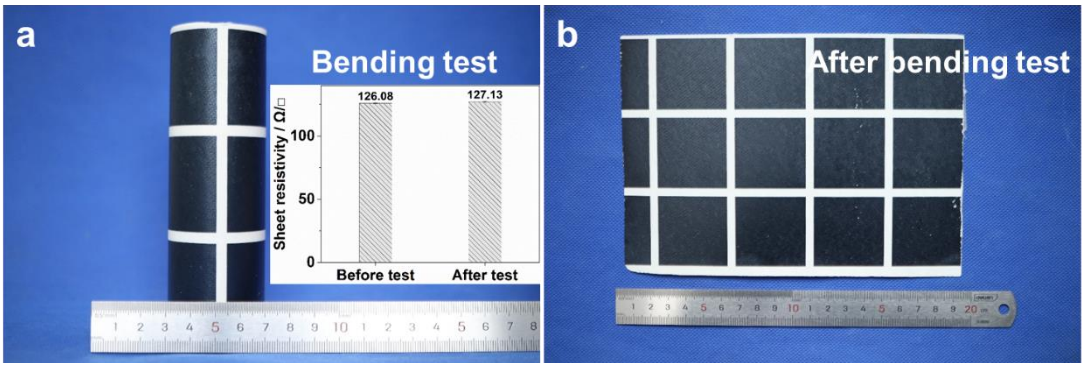


Supplementary Fig. 14 Photographs of the $RuO_2$/glass metasurface (a) in and (b) after the bending test. Inset of (a): sheet resistivity change rate of the patches before and after bending test.

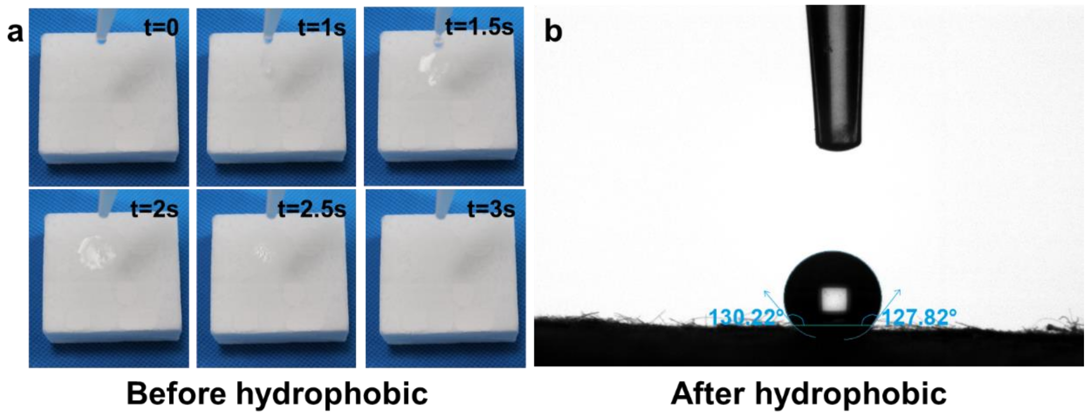


Supplementary Fig. 15 Hydrophobicity test of alumina aerogel composites (a) before and (b) after hydrophobic treatment.

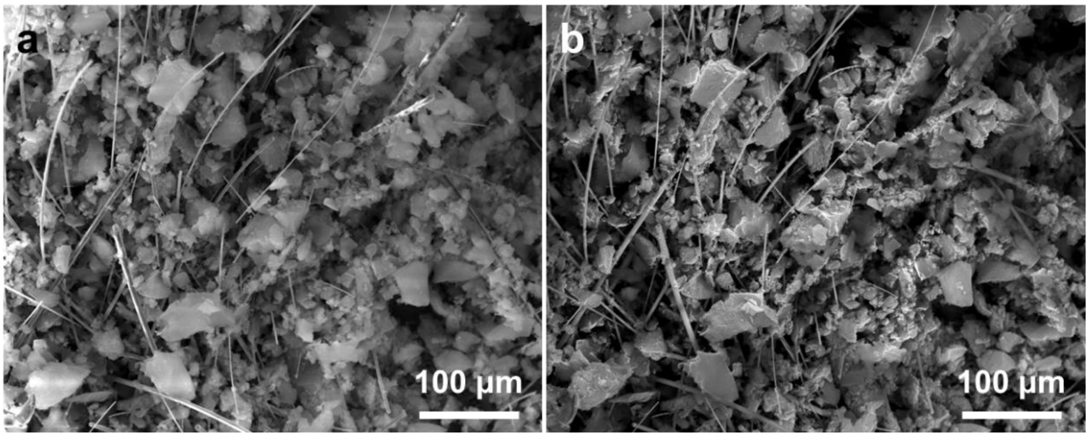


Supplementary Fig. 16 (a) SEM and (b) BSE images of the cross-section of the alumina aerogel composites.

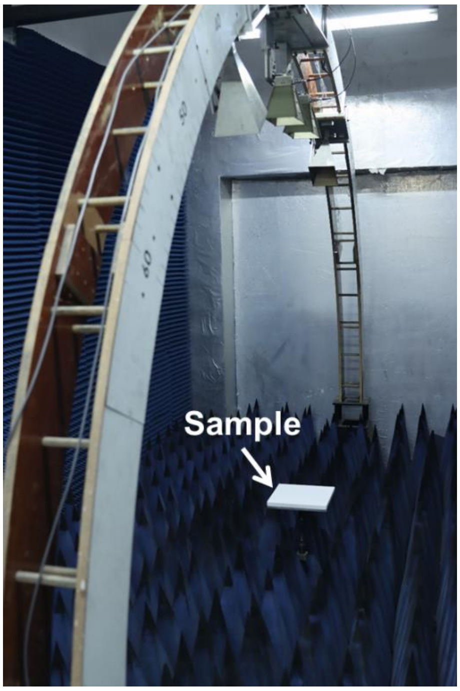


Supplementary Fig. 17 Photograph of NRL arch device.

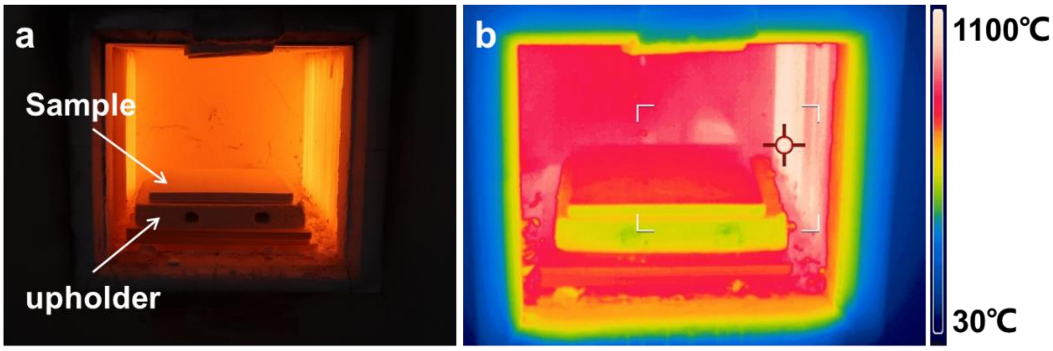


Supplementary Fig. 18 (a) Digital photograph and (b) infrared image of MTL integrated composites located in a muffle furnace of 1000 °C during thermal shock testing.

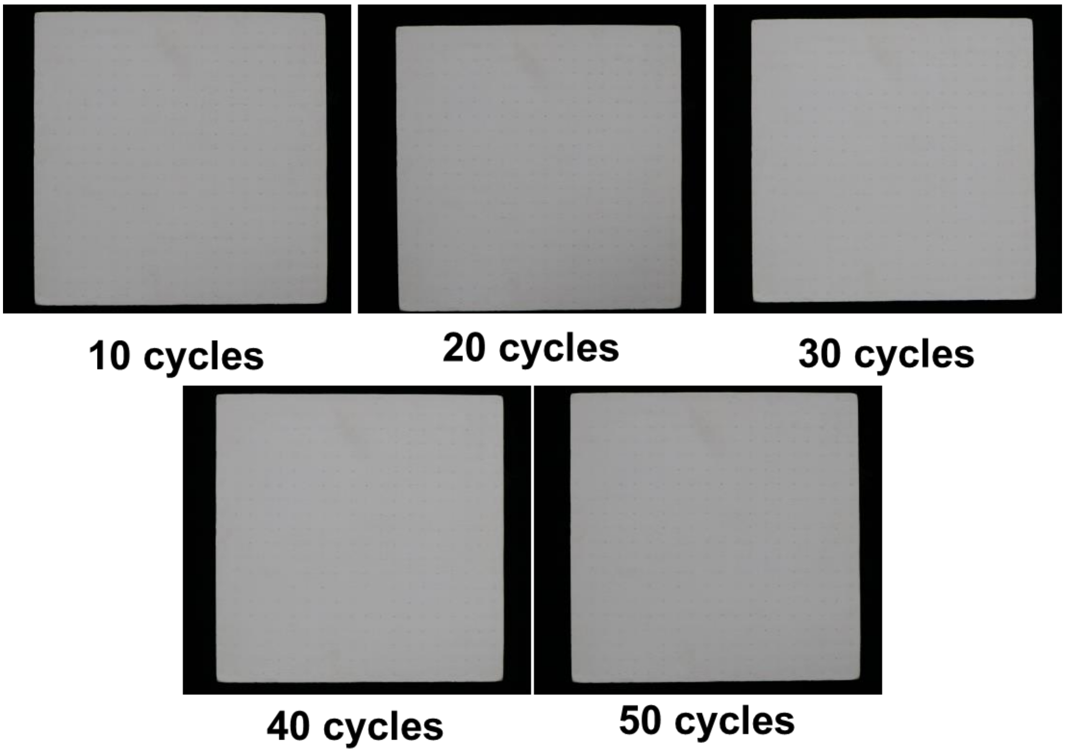


Supplementary Fig. 19 Digital photographs of MTL integrated composite after thermal shock at 25 ℃ ~ 1000 ℃.

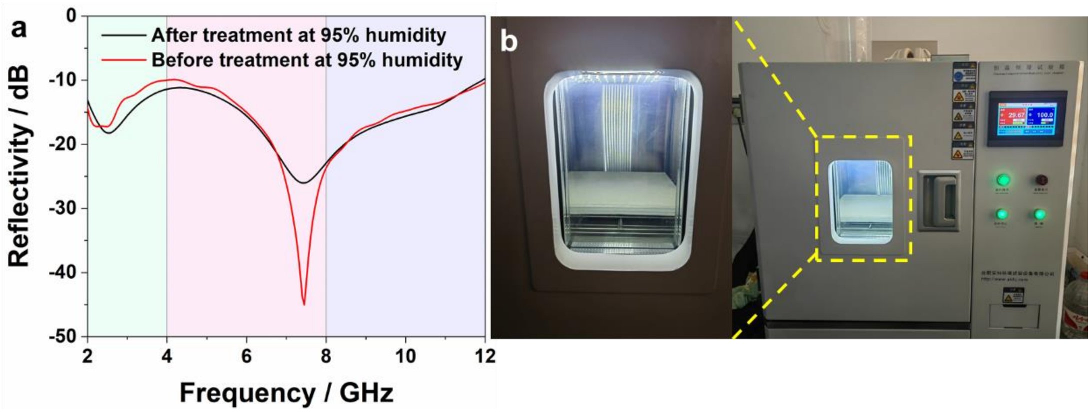


Supplementary Fig. 20 (a) Reflectivity of the MTL integrated composites before and after treatment at 95% humidity for 12 h. (b) Picture of a sample in a constant-temperature and constant-humidity test chamber.

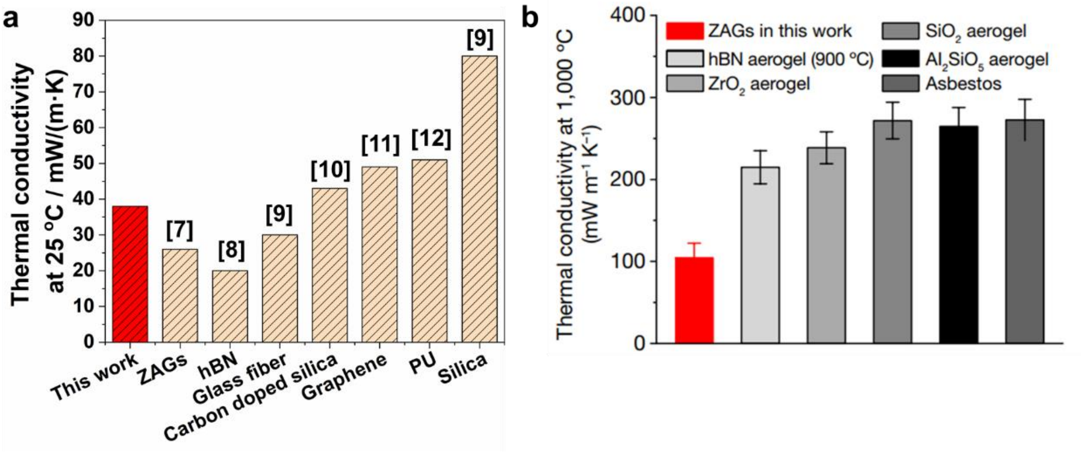


Supplementary Fig. 21 (a) Comparison of the thermal conductivities of the alumina aerogel composites with those of other aerogels at 25 °C[1, 2, 3, 4, 5, 6]. (b) Thermal conductivity of other aerogels at 1000 °C (the picture is from reference[1]).

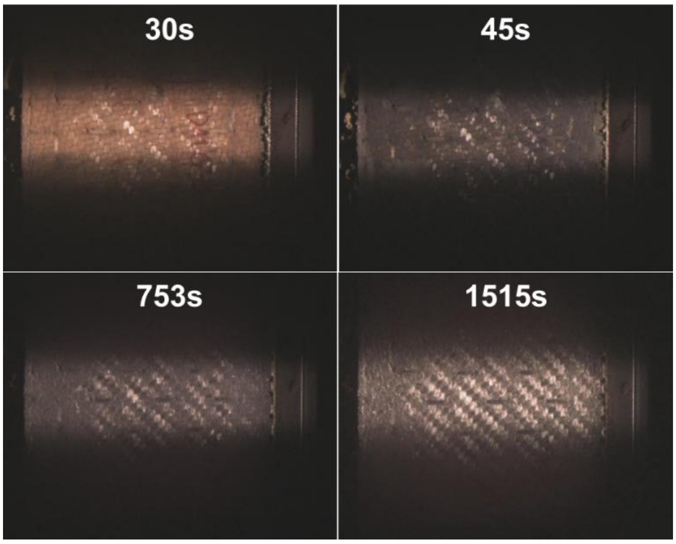

Supplementary Fig. 22 Typical wind tunnel photographs of MTL integrated composite at different time.

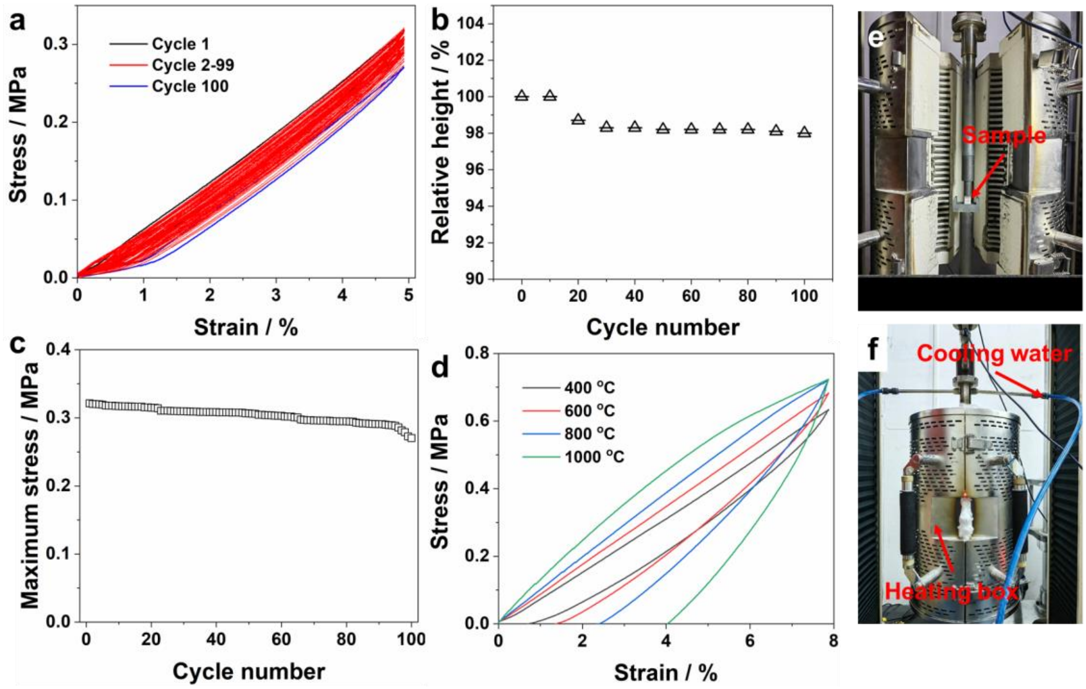


Supplementary Fig. 23 The compressive cyclic performance of MTL integrated composite materials under fatigue and high temperature conditions. (a) Stress–strain curves, (b) relative height, and (c) maximum stress of 100 compressive cycles of the MTL integrated composites at 5% strain. (d) Compressive stress–strain curves of the MTL integrated composites at 8% strain at 400 °C, 600 °C, 800 °C, and 1000 °C. (e, f) Testing equipment for determining high-temperature compression properties.

Supplementary Table 3 Comparison of microwave absorption properties for different types of microwave-absorbing materials

| Materials system | Structure type | Relative bandwidth (%) | Absolute bandwidth (GHz) | |
|---|---|---|---|---|
| | | | Room-temperature | High-temperature |
| This work | Metasurface | 143 (1000°C) | 2~12 | 2~12 (1000°C) |
| $C\text{-}SiO_2/SiO_2$ [7] | Metasurface | 67 (1000°C) | 5.1~8.2 | 3~6 (1000°C) |
| $C\text{-}SiO_2/SiO_2$ [8] | Metasurface | 113 (1000°C) | 3~18 | 5~18 (1000°C) |
| $C_f\text{-}Al_2O_{3f}/SiOC$ [9] | Metasurface | 64.7 (700°C) | 10~17.2 | 9.2~18 (700°C) |
| $Pt\text{-}MoSi_2\text{-}TiB_2/Al_2O_3$ [10] | Metasurface | 12.9 (800°C) | / | 10.9~12.4 (800°C) |
| CNTs@BN/SiC [11] | 3D-metamaterials | 151 (1000°C)<br>60.5 (600°C)<br>57.5 (400°C) | 9.9~18 | 2.5~18 (1000°C)<br>9.64~18 (600°C)<br>9.96~18 (400°C) |
| $SiC_f/Si_3N_4$ [12] | 3D-metamaterials | 33.8 (500°C) | 14.8~18 | 12.8~18 (500°C) |
| SiC [13] | 3D-metamaterials | 155.6 (1000°C)<br>140.4 (800°C) | 7~40 | 5~40 (1000°C) |
| SiOC [14] | 3D-metamaterials | 9.1 (800°C) | 13.9~14.9 | 13.7~15 (800°C) |
| $TiB_2\text{-}Al_2O_3$ [15] | Scattering metamaterials | 91 (1000°C) | 4.9~14 | 4.9~13.1 (1000°C) |
| SiC [16] | Honeycomb | 44.4 (1000°C) | 6.8~11.2 | 7~11 (1000°C) |
| $Al_2O_3\text{-}TiC$ [17] | Coating | 36.1 (800°C) | 11.5~12.4 | 8.4~12.1 (800°C) |
| $SiC_f/Si_3N_4$ [18] | Dallenbach structure | 38.6 (600°C) | 12~16.5 | 9.6~14.2 (600°C) |
| $SiC_f/SiC\text{–}Al_2O_3$ [19] | Dallenbach structure | 23.7 (600°C) | 9.7~12.4 | 8.2~10.4 (600°C) |

| | | | | |
|---|---|---|---|---|
| $Si_3N_4$–$SiC/SiO_2$[20] | Dallenbach structure | 39.2 (600℃) | 8.4~12.4 | 8.2~12.2 (600℃) |

Supplementary Table 4 Thermal conductivity of the MTL integrated composites at room temperature and 1000 °C

| Temperature (°C) | Method | Test criteria | Sample size (mm) | Thermal conductivity (mW/(m·K)) |
|---|---|---|---|---|
| 25 | Heat Flow Meter Method | GB/T 10295-2008 | 300 × 300 × 18 | 38 |
| 1000 | Water Flow Plate Method | YB/T 4130-2005 | Φ180 × 18 | 143 |

## Reference:


1. Guo J, *et al.* Hypocrystalline ceramic aerogels for thermal insulation at extreme conditions. *Nature* 606, 909-916 (2022).

2. Xu X, *et al.* Double-negative-index ceramic aerogels for thermal superinsulation. *Science* 363, 723-727 (2019).

3. Koebel M, Rigacci A, Achard P. Aerogel-based thermal superinsulation: an overview. *Journal of Sol-Gel Science and Technology* 63, 315-339 (2012).

4. Lee D, Stevens PC, Zeng SQ, Hunt AJ. Thermal characterization of carbon-opacified silica aerogels. *Journal of Non-Crystalline Solids* 186, 285-290 (1995).

5. Xie Y, Xu S, Xu Z, Wu H, Deng C, Wang X. Interface-mediated extremely low thermal conductivity of graphene aerogel. *Carbon* 98, 381-390 (2016).

6. Wu J-W, Sung W-F, Chu H-S. Thermal conductivity of polyurethane foams. *International Journal of Heat and Mass Transfer* 42, 2211-2217 (1999).

7. An Z, Li Y, Luo X, Huang Y, Zhang R, Fang D. Multilaminate metastructure for high-temperature radar-infrared bi-stealth: Topological optimization and near-room-temperature synthesis. *Matter* 5, 1937-1952 (2022).

8. An Z, Huang Y, Zhang R. High-temperature multispectral stealth metastructure from the microwave-infrared compatible design. *Composites Part B: Engineering* 259, 110737 (2023).

9. Yang F, *et al.* In-situ construction of carbon fiber gradient periodic structure in Al2O3f/SiOC composites for ultra-broadband and high-temperature electromagnetic wave absorption. *Journal of Materials Science & Technology* 194, 87-97 (2024).

10. Zhao S, *et al.* High temperature metamaterial enhanced electromagnetic absorbing coating prepared with alumina ceramic. *Journal of Alloys and Compounds* 874, 159822 (2021).

11. Ren B, Deng Y, Jia Y, Han L, Wang X, Li H. Achieving broadband electromagnetic absorption at a wide temperature range up to 1273 K by metamaterial design on polymer-derived SiC-BN@CNT ceramic composites. *Chemical Engineering Journal* 478, (2023).

12. Zhou Q, *et al.* Multiscale designed SiCf/Si3N4 composite for low and high frequency cooperative electromagnetic absorption. *Journal of the American Ceramic Society* 101, 5552-5563 (2018).

13. Zhou N, *et al.* Stereolithographically 3D Printed SiC Metastructure for Ultrabroadband and High Temperature Microwave Absorption. *Advanced Materials Technologies* 8, 2201222

(2023).

14. Zhou R, Wang Y, Liu Z, Pang Y, Chen J, Kong J. Digital Light Processing 3D-Printed Ceramic Metamaterials for Electromagnetic Wave Absorption. *Nano-Micro Letters* 14, 122 (2022).

15. Ji C, Peng J, Yuan L, Huang C, Luo X. All-Ceramic Coding Metastructure for High-Temperature RCS Reduction. *Advanced Engineering Materials* 24, 2101503 (2022).

16. Wang W, Li Z, Gao X, Huang Y, He R. Material extrusion 3D printing of large-scale SiC honeycomb metastructure for ultra-broadband and high temperature electromagnetic wave absorption. *Additive Manufacturing* 85, 104158 (2024).

17. Shao T*, et al.* High temperature absorbing coatings with excellent performance combined Al2O3 and TiC material. *Journal of the European Ceramic Society* 40, 2013-2019 (2020).

18. Zhou Q, Yin X, Ye F, Tang Z, Mo R, Cheng L. High temperature electromagnetic wave absorption properties of SiCf/Si3N4 composite induced by different SiC fibers. *Ceramics International* 45, 6514-6522 (2019).

19. Mu Y*, et al.* Temperature-dependent dielectric and microwave absorption properties of SiCf/SiC–Al2O3 composites modified by thermal cross-linking procedure. *Journal of the European Ceramic Society* 35, 2991-3003 (2015).

20. Li M*, et al.* High-temperature dielectric and microwave absorption properties of Si3N4–SiC/SiO2 composite ceramics. *Journal of Materials Science* 50, 1478-1487 (2015).
