# Supplementary material for: Metasurface-based multifunctional composites with ultra-robust broadband microwave absorption up to 1000 °C
Source: Nat Commun. 2025 Nov 25;16:10427. doi: 10.1038/s41467-025-66317-3 (PMC12647858; doi:10.1038/s41467-025-66317-3)
Supplement: Supplementary file 2 — Description of Additional Supplementary Files [file 41467_2025_66317_MOESM2_ESM.pdf]

Supplementary Video 1. The video of a butane flame heating a lily flower through a MTL integrated composite plate.

Supplementary Video 2. The video of MTL integrated composites withstand crushing by a 0.8 t car.

Supplementary Video 3. The video of one loading–unloading compressive cycle of MTL integrated composite at 10% strain.
