# Supplementary material for: Metasurface-based multifunctional composites with ultra-robust broadband microwave absorption up to 1000 °C
Source: Nat Commun. 2025 Nov 25;16:10427. doi: 10.1038/s41467-025-66317-3 (PMC12647858; doi:10.1038/s41467-025-66317-3)
Supplement: Supplementary file 6 — Transparent Peer Review file [file 41467_2025_66317_MOESM6_ESM.pdf]

nature portfolio

Peer Review File

Metasurface-based multifunctional composites with ultra-robust broadband microwave absorption up to 1000 °C

Corresponding Author: Professor Haitao Liu

This file contains all reviewer reports in order by version, followed by all author rebuttals in order by version.

Version 0:

Reviewer comments:

Reviewer #1

(Remarks to the Author)
This study presents RuO_2/glass-based microwave-absorbing metasurfaces featuring a low temperature coefficient of resistance, and fabricates multifunctional composites that integrate microwave absorption, thermal insulation, and load-bearing capabilities. In general, this work reports noteworthy findings and demonstrates innovation in materials design. However, there are several ambiguous statements in the interpretation of results. In particular, with regard to the RuO_2/glass metasurface, the experimental rationale and processing sequence remain unclear, and the performance explanation is insufficient. Before this manuscript can be considered for publication, the following issues should be addressed:

1. The mechanism underlying the workability of RuO_2/glass composites at various temperatures remains unclear. For instance, the role of the interfacial structure and residual stress between the two phases is not well understood. To clarify these aspects, detailed information on the composition of the glass matrix and key physical properties of the bulk glass—such as the glass transition temperature (Tg), thermal expansion coefficient, and density—should be provided. Additionally, basic data on the raw materials, including the particle size distributions of both the RuO_2 and glass powders, are essential for comprehensive analysis.

2. Given that the RuO_2/glass composite can be sintered at 1000 °C, it is unclear how the metasurface retains structural and electrical stability under such high operating temperatures. Please clarify the thermal stability and potential phase evolution under service conditions.

3. To ensure uniform dispersion of RuO_2 nanoparticles within the glass matrix, further evidence beyond the TEM image in Fig. 3a is needed. It is recommended to include SEM images combined with EDS elemental mapping to confirm morphology and compositional homogeneity.

4. The surface characteristics of the RuO_2/glass metasurface and the strategy for controlling its resistivity are not clearly discussed. Please elaborate on the surface microstructure and its correlation with electrical behavior.

5. Based on the tunneling barrier model proposed in Fig. 3e, the interparticle distance between RuO_2 nanoparticles plays a critical role in controlling the composite's resistance. It is suggested to provide experimental data showing the correlation between RuO_2 content and resistance to better understand the temperature-dependent percolation behavior.

6. Please explain the observed increase in resistance in the temperature range of 800–900 °C as shown in Fig. 3f. Possible mechanisms such as microstructural changes, phase transformation, or sintering effects should be considered.

7. The comparison with other commonly used resistive materials is misleading. Although RuO_2/glass exhibits favorable properties, it does not possess the lowest room-temperature TCR. Please include comparisons with low-TCR materials for a more balanced evaluation.

8. As the aerogel composites were treated to become hydrophobic, please provide contact angle measurements before and after surface modification to support this claim.

9. While the load-bearing capability of the MTL-integrated composites is promising, fatigue resistance under cyclic loading

and the temperature dependence of mechanical properties are critical for real applications. Please consider including such measurements.

10. Given the inherent brittleness of glass, what is the fracture toughness of the RuO_2/glass resistive materials? Please discuss how mechanical damage or microcracking might influence the overall performance and reliability of the MTL-integrated composite.

11. Regarding the mechanical properties part: it is unclear how the thermal insulation and electromagnetic wave absorption change under a stressed (load-bearing) condition. Is the bandwidth of electromagnetic wave absorption related to the microstructure of the aerogel?

12. The impact of the supercritical drying process on the aerogel's pore structure is not adequately addressed. Please provide detailed pore size distribution data or SEM/TEM images to clarify this relationship.

13. Since the performance of aerogels can be significantly affected by environmental conditions, particularly humidity, please evaluate how the MTL-integrated composites behave under varying humidity levels or prolonged moisture exposure. Such data would improve understanding of their long-term stability and functionality.

Minor comments:
1. Most equations are presented without definitions of variables or references, which makes them difficult to interpret. Please ensure that all variables are defined and cited appropriately.

2. Please provide the thermal conductivity values of the MTL-integrated composites and compare them with those of state-of-the-art thermal insulation materials.

Reviewer #2

(Remarks to the Author)
Referee report

In the paper entitled " Metasurface-based multifunctional composites with ultra-robust broadband microwave absorption up to 1000 °C”, Xinyuan Lv et al report structure is capable of maintaining absorption in the frequency range 2~12 GHz at temperatures up to 1000 °C. The microwave-absorbing structure comprises a pair of RuO2/glass metasurfaces combined with layers of alumina aerogel and ceramic composites. The performed measurements and developed theory show a broadband absorption ability of < -10 dB at 2~12 GHz without any degradation under varying temperature (25 °C ~ 1000 °C).

I recommend this paper for publication in Nature Communication. Authors report significant advance within a specialist field of the microwave absorbing structures. The demonstrated outstanding temperature stability of the fabricated structures is partially because no carbon materials, which are widely used in microwave and THz absorbers, are involved. The fabricated RuO2/glass resistor paste effectively replaces carbon providing strong absorption performance, while composite layers ensure the temperature stability.

Reviewer #3

(Remarks to the Author)
This manuscript reports on the design and fabrication of a RuO_2/glass-based metasurface integrated within a multilayer composite structure (MTL) for high-temperature microwave absorption. The authors claim ultra-broadband microwave absorption (2–12 GHz) up to 1000 °C, enabled by a temperature-insensitive resistive mechanism attributed to quantum tunneling in the RuO_2/glass material.

While the experimental results are promising and the overall architecture is intriguing for high-temperature applications, I find several critical scientific and methodological issues that, in my view, preclude publication in Nature Communications in its current form.

Please see my comment below:
1. Lack of Direct Evidence for the Tunneling Mechanism:
The central claim of the manuscript is that quantum tunneling across nanoscale RuO_2-glass interfaces is responsible for the ultra-low TCR (~ –95 to –365 ppm/°C). However, no direct electrical measurements (e.g., temperature-dependent I–V characteristics, non-linear conduction analysis, or Fowler–Nordheim plots) are provided. The model presented for the barrier and bulk resistance is theoretical and not supported by quantitative fitting. Given that the tunneling hypothesis is a central novelty claim, its current support is insufficient for a high-impact journal.

2. Inadequate Electromagnetic Modelling:
The use of HFSS and equivalent circuit modelling is a good start, but the EM response lacks deeper analysis. There is no modal decomposition, no mapping of current loss densities across layers, nor is there evidence of destructive or constructive interference effects.
The two-resonance explanation (S- and X-band) is based on electric field snapshots but does not rigorously prove the mechanism of field coupling between layers. A more complete EM loss analysis is necessary to substantiate the absorber's

behavior.
While the top and bottom metasurfaces share the same sheet resistance but differ in mesh geometry, no analysis is provided on the distinct roles of inductive versus capacitive responses. Is there any evidence for trapped-mode resonances arising from the dual-metasurface configuration?

3. Equivalent Circuit Model and Input Impedance Matching
The proposed RLC ladder circuit model is mathematically defined from Z_1 to Z_6, but the physical correlation between each subcircuit and specific absorption features is unclear. Has this model been experimentally validated, and what is the quantitative error between simulation and measurement across the 2–12 GHz range?
The model assumes ideal dielectric behavior for intermediate layers. However, aerogel composites exhibit strong frequency-dependent permittivity (ε') and dielectric loss (tanδ), particularly from 25 to 1000 °C. Has any sensitivity analysis been conducted to quantify how these variations affect impedance matching and absorption?

4. The authors attribute the dominant loss mechanism to conductor (resistive) loss. However, aerogel dielectric loss (tanδ) also increases significantly at high temperatures. Are there experiments or modeling that quantitatively separate the contributions of dielectric vs. resistive losses?

5. The temperature-dependent dielectric properties (ε', tanδ) are measured using cavity perturbation. However, this technique is known to suffer from increasing error at high temperatures. Have independent methods such as waveguide transmission or THz-TDS been used to validate these measurements?

6. The EM absorption after thermal shock is highly recommended.

7. Fig. 3f and g show the peak around 850 oC. An explanation is encouraged.

Version 1:

Reviewer comments:

Reviewer #1

(Remarks to the Author)
The authors have satisfactorily addressed my previous comments, and I recommend the manuscript for publication in Nature Communications in its current form.

Reviewer #3

(Remarks to the Author)
The authors have undertaken a thorough and well-prepared revision of the manuscript. They have adequately addressed the concerns I raised, as well as those posed by the other reviewers. The revisions substantially improve the clarity, rigor, and overall quality of the work. However, I still have other doubts.
1. How did the authors measure permittivity using the VNA method at such high temperatures?
2. How thick is of RuO2/glass fiber layer coating?
3. It is great if the reflectivity properties of the single resistive layer on Al2O3f/Al2O3 are provided.
4. The unit of thermal conductivity in Fig. S21a may be wrong.
5. References are needed in lines 46-48, 56-59, 71-73.
6. In lines 76-81, the authors have an interesting comment on how absorption and cooling performance rely on thickness. Is it solved in this work?

Version 2:

Reviewer comments:

Reviewer #3

(Remarks to the Author)
Thanks to the authors for addressing my comments thoroughly.
I believe the current version of the manuscript is ready for publication.

Open Access This Peer Review File is licensed under a Creative Commons Attribution 4.0 International License, which permits use, sharing, adaptation, distribution and reproduction in any medium or format, as long as you give appropriate credit to the original author(s) and the source, provide a link to the Creative Commons license, and indicate if changes were made.
In cases where reviewers are anonymous, credit should be given to 'Anonymous Referee' and the source.
The images or other third party material in this Peer Review File are included in the article's Creative Commons license, unless indicated otherwise in a credit line to the material. If material is not included in the article's Creative Commons license and your intended use is not permitted by statutory regulation or exceeds the permitted use, you will need to obtain permission directly from the copyright holder.
To view a copy of this license, visit https://creativecommons.org/licenses/by/4.0/

Responses to reviewer #1:

We sincerely thank the reviewer for your valuable suggestions regarding various material characterization. These insightful comments have significantly enhanced our understanding of the RuO_2/glass composite's structure-property relationships. The recommended characterizations provided crucial new perspectives on material's features and improved the quality of this article.

Q1: The mechanism underlying the workability of RuO_2/glass composites at various temperatures remains unclear. For instance, the role of the interfacial structure and residual stress between the two phases is not well understood. To clarify these aspects, detailed information on the composition of the glass matrix and key physical properties of the bulk glass-such as the glass transition temperature (T_g), thermal expansion coefficient, and density-should be provided. Additionally, basic data on the raw materials, including the particle size distributions of both the RuO_2 and glass powders, are essential for comprehensive analysis.

Answer: We thank the reviewer for this comment. We have added the phase composition and elemental content of the glass. According to the XRD patterns (**Fig. 1**), the glass starts crystallizing at ~850 °C. After sintering at 1000 °C (the sintering temperature in this study), the phases of the glass consisted mainly of K-Na feldspar, aluminosilicate, and quartz. The quantitative elemental analysis via inductively coupled plasma (ICP) (**Table 1**) revealed that the glass powder contained Al (10.57 wt.%), K (4.904 wt.%), Na (1.169 wt.%) and Si (19.31 wt.%). Additionally, the Ba and Zn detected via the ICP test may be impurities.

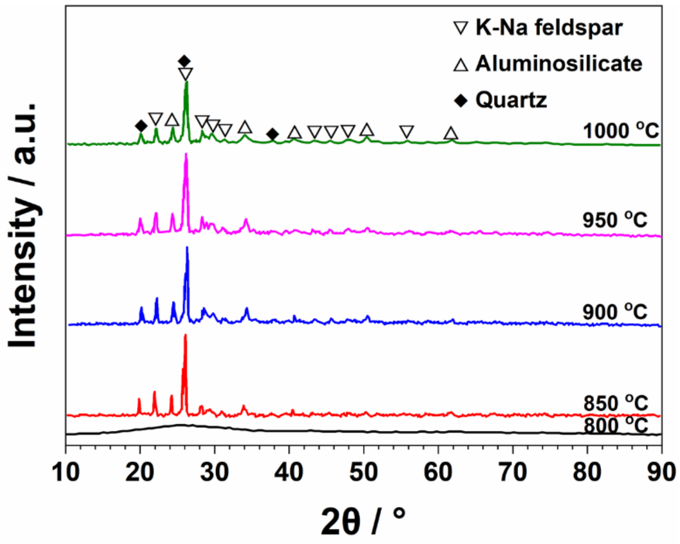

Fig. 1 XRD patterns of the glass at different sintering temperatures.

Table 1 Element contents of the glass samples measured via the ICP method.

Element	Content/wt.%
Al	10.57
K	4.904
Zn	4.755
Ba	0.12
Na	1.169
Si	19.31
O	59.172

For the key physical properties of the bulk glass, we obtained the coefficient of thermal expansion (CTE) and density of the bulk glass were 8 × 10^{-6} K^{-1} and 2.53 g/cm^3, respectively. To identify the glass transition temperature (T_g) of the glass powder, DSC tests were performed (**Fig. 2**). In the purple region of **Fig. 2**, the baseline of the DSC curve shows an obvious shift down. Thus, the T_g of the glass powder was determined to be ~675 °C through differentiation of the DSC curve in the purple region.

Finally, we measured the particle size distributions of the RuO_2 and glass powders (**Fig. 3**) via a laser particle analyzer (Malvern MS3000, Malvern Instruments,

UK). The particle sizes of RuO_2 and glass powder showed bimodal and unimodal distributions, respectively. According to the detailed particle size analysis data (**Table 2**), the d_{50} and specific surface area of RuO_2 were 1.43 μm and 21.9 m^2/g, and the d_{50} and specific surface area of the glass were 5.51 μm and 1.6 m^2/g, respectively.

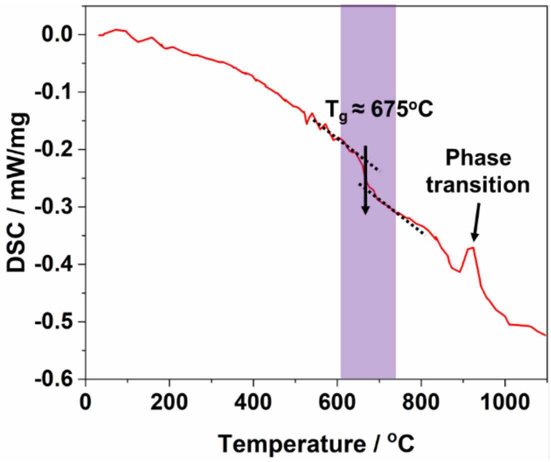

Fig. 2 DSC curve of the glass powder at 25 °C ~ 1100 °C.

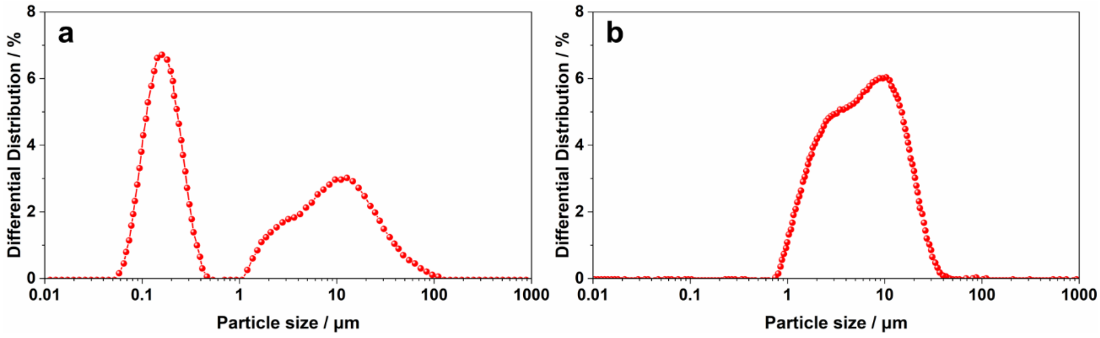

Fig. 3 Particle size distributions of (a) RuO_2 and (b) the glass powder.

Table 2 Analysis results of the particle sizes of RuO_2 and glass powder.

Powder	d_{10} / μm	d_{50} / μm	d_{90} / μm	Specific surface area/m^2/g
RuO_2	0.10	1.43	19.86	21.9
Glass	1.61	5.51	16.11	1.6

Revised version: Fig. 1 has been added to Supplementary file as **Supplementary Fig. 7.** The material information has been included in **Supplementary Note 1** and **Supplementary Table 1**. The related revised text can be

found in P13 of revised manuscript and P2 of revised supplementary file:

P13 of revised manuscript: In the X-ray diffraction (XRD) pattern (**Fig. 3b**), only highly crystalline RuO_2 was detected, whereas the glass matrix was not found due to its low crystallinity, although it begins to crystallize from ~ 850 °C (**Supplementary Fig. 7**).

P2 of revised supplementary file: RuO_2 particles with size of $d_{10}/d_{50}/d_{90}$ = 0.1 μm/1.43 μm/19.86 μm were supplied from Sino-Platinum Metals Co., Ltd. China.

Glass slags were self-made in lab and their basic properties were provided in **Supplementary Table 1**.

Supplementary Table 1 The basic properties of the glass

Density (g/cm^3)	CTE (K^{-1})	$d_{10}/d_{50}/d_{90}$ (μm)	Element contents (wt.%)	Phase composition
2.53	8×10^{-6}	1.61/5.51/16.11	Al:10.57<br>K:4.904<br>Zn:4.755<br>Ba:0.12<br>Na:1.169<br>Si:19.31<br>O:59.172	K-Na feldspar,<br>Aluminosilicate,<br>Quartz

Q2: Given that the RuO_2/glass composite can be sintered at 1000 °C, it is unclear how the metasurface retains structural and electrical stability under such high operating temperatures. Please clarify the thermal stability and potential phase evolution under service conditions.

Answer: We thank the reviewer for this comment. Given that the glass begins to crystallize at 850 °C (**Fig. 1**), its phase and structure can remain stable at high temperatures after sintering of 1000 °C. We subjected the sintered RuO_2/glass

resistive patch to a heat treatment for 10 h at 1000 °C. The SEM and BSE images show that the microstructure and distribution of the glass matrix remain almost unchanged (**Figs. 4b** and **4c**). Then, we measured the sheet resistivity of the RuO_2/glass resistive patch at different holding time at 1000 °C. The sheet resistivity of the RuO_2/glass resistive patch first increased but then decreased, with a maximum variation rate of 16% (at 3~5 h) (**Fig. 4a**). Given that the microwave absorption performance of the MTL integrated composites remains nearly unchanged even with a 35% variation in the sheet resistivity of the RuO_2/glass resistive patch, the 16% variation in electrical properties likewise has a negligible influence on its absorption performance.

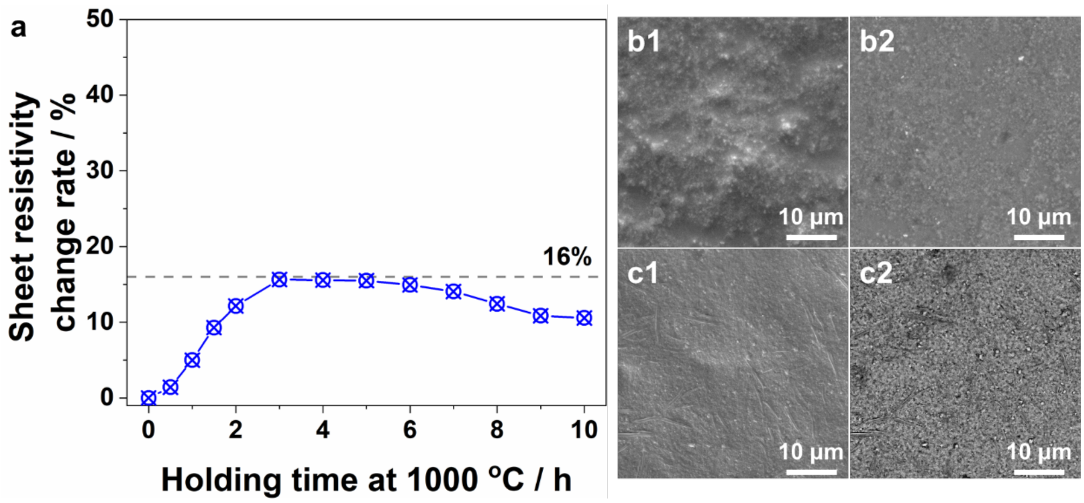

Fig. 4 (a) Sheet resistivity change rate of the RuO_2/glass resistive patch at different holding times at 1000 °C. SEM and BSE images of RuO_2/glass resistive patches (b1, b2) before and (c1, c2) after heat treatment at 1000 °C for 10 h.

Revised version: Fig. 4a has been added to Supplementary file as **Supplementary Fig. 12**. The related revised text can be found in P16 of revised manuscript:

Furthermore, the resistive patch was maintained at 1000 °C for 10 h, with its sheet resistivity increasing by no more than 16% compared to the initial value at 1000 °C (**Supplementary Fig. 12**). This further confirms the stability of its high-temperature electrical performance.

Q3: To ensure uniform dispersion of RuO_2 nanoparticles within the glass matrix, further evidence beyond the TEM image in Fig. 3a is needed. It is recommended to include SEM images combined with EDS elemental mapping to confirm morphology and compositional homogeneity.

Answer: We thank the reviewer for this comment. We observed the morphology of the RuO_2/glass resistive patch via SEM in secondary electron (SE) and backscattered electron (BSE) modes (**Fig. 5**). In SE mode, slight surface roughness of the resistive patch can be observed, and the white spots represent RuO_2 particles (**Figs. 5a1, b1, c1**). The BSE images provide a clearer observation of the distribution of RuO_2 particles, demonstrating their highly uniform dispersion (**Figs. 5a2, b2, c2**). It can also be seen from the EDS mapping (**Figs. 5d, 5e, 5f**) that the distribution of the Ru, Si, and O elements is very uniform.

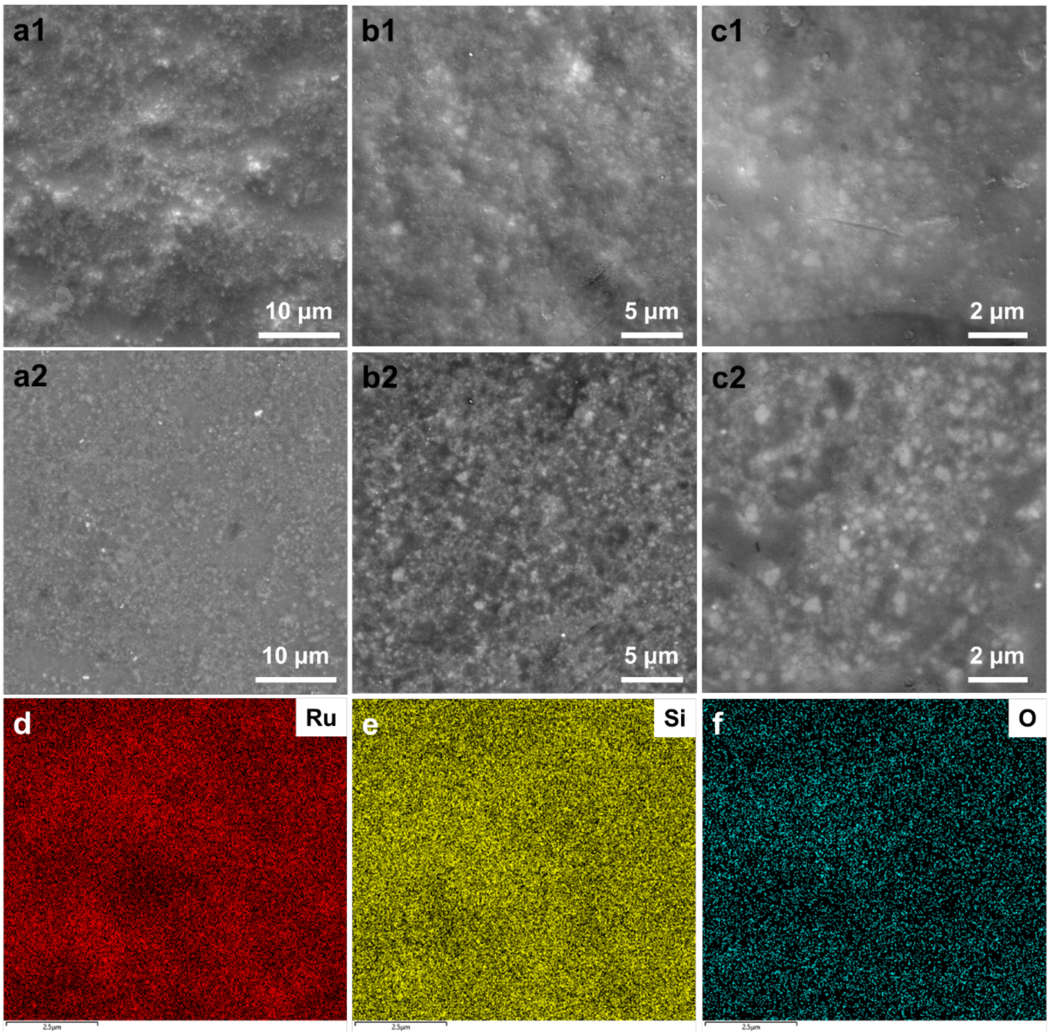

Fig. 5 (a1, b1, c1) SEM and (a2, b2, c2) BSE images of the surface of the RuO_2/glass resistive patch at different magnifications. (d, e, f) EDS mappings of Ru, Si, and O elements.

Revised version: Fig. 5 has been added to Supplementary file as **Supplementary Fig. 6**. The related revised text can be found in P13 of revised manuscript and P4&P5 of revised supplementary file:

P13 of revised manuscript: RuO_2 nanoparticles were uniformly distributed in the amorphous glass matrix (**Fig. 3a** and **Supplementary Fig. 6**).

P4&P5 of revised supplementary file: The backscattered electron (BSE) morphology and elemental mapping of the samples were examined using a BSE

detector and an energy-dispersive spectroscopy (EDS) detector equipped on the SEM.

Q4: The surface characteristics of the RuO_2/glass metasurface and the strategy for controlling its resistivity are not clearly discussed. Please elaborate on the surface microstructure and its correlation with electrical behavior.

Answer: We thank the reviewer for this comment. The strategy for tuning the sheet resistivity of RuO_2/glass resistive patches is to control the content of RuO_2. We tested the sheet resistivity of the resistive patches with different RuO_2 contents (**Fig. 6**). When the RuO_2 content increased from 15% to 52%, the sheet resistivity of the resistive patches first sharply decreased from 5×10^5 Ω/□ to 5×10^4 Ω/□ and then slowly decreased to 50 Ω/□. The electrical behavior of the RuO_2/glass resistive patches conforms to percolation theory. When the content of conductive particles is low, the particles are dispersed evenly, and the material has an extremely high resistivity. When the content of conductive particles reaches a certain threshold, they begin to form a continuous conductive network, and the resistivity decreases sharply, eventually reaching a smooth trend of change. Therefore, the electrical behavior of the RuO_2/glass metasurface is primarily governed by the RuO_2 content and is not significantly correlated with its surface microstructure.

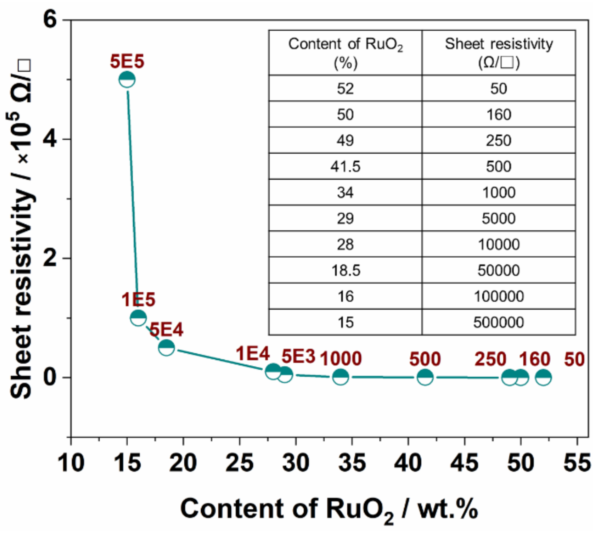

Fig. 6 Sheet resistivities of RuO_2/glass resistive patches with different contents of RuO_2.

Revised version: Fig. 6 has been added to Supplementary file as **Supplementary Fig. 5**. The related revised text can be found in P13 of revised manuscript:

The sheet resistivity of RuO_2/glass resistive patches can be easily controlled by adjusting the RuO_2 content, and their electrical behavior follows percolation theory (**Supplementary Fig. 5**).

Q5: Based on the tunneling barrier model proposed in Fig. 3e, the inter-particle distance between RuO_2 nanoparticles plays a critical role in controlling the composite's resistance. It is suggested to provide experimental data showing the correlation between RuO_2 content and resistance to better understand the temperature-dependent percolation behavior.

Answer: We thank the reviewer for this comment. We provided a plot of RuO_2 content versus sheet resistivity in our response to **Question 4** (**Fig. 6**). When the sheet resistivity of the resistive patches decreases by one order of magnitude from 5×10^5

Ω/□ to 5 × 10^4 Ω/□, the RuO_2 content needs to increase by only 3.5%. For each order of magnitude decrease in the sheet resistivity, the required increase in the RuO_2 content is 9.5%, 12.5%, and 10.5%, respectively. Eventually, the sheet resistivity is reduced to 50 Ω/□ when the RuO_2 content is 52%. Therefore, the sheet resistivity of the RuO_2/glass resistive patches clearly exhibits percolation behavior with respect to the RuO_2 content.

Q6: Please explain the observed increase in resistance in the temperature range of 800~900 °C as shown in Fig. 3f. Possible mechanisms such as microstructural changes, phase transformation, or sintering effects should be considered.

Answer: We thank the reviewer for this comment. We think that the increase in resistance observed in the RuO_2/glass resistive patch at 800~900 °C originates from the phase transition of glass matrix. The endothermic peak in the DSC curve of the glass (**Fig. 7**) within this temperature range indicates a phase transition. The phase transition of the glass matrix may induce a change in its CTE or TCR, thereby leading to the increase of barrier resistance. Additionally, RuO_2 possesses a positive intrinsic TCR, meaning that its intrinsic resistance also increases with increasing temperature. Consequently, at 800~900 °C, both the intrinsic resistance and the barrier resistance increase, ultimately leading to an overall increase in the resistance of the RuO_2/glass resistive patch.

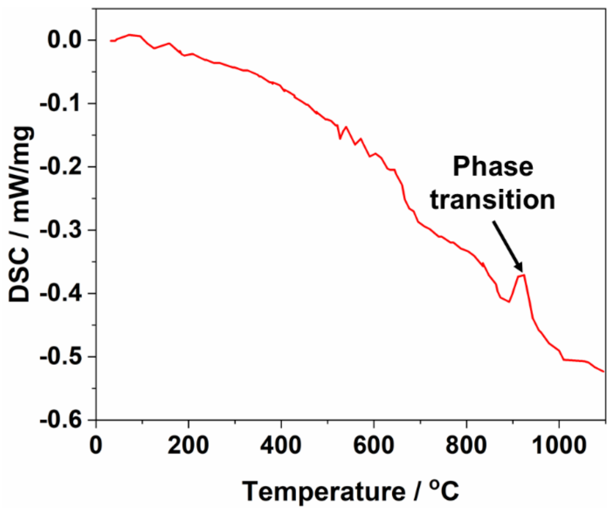

Fig. 7 DSC curve of the glass from room temperature to 1100 °C.

Revised version: Fig. 7 has been added to Supplementary file as **Supplementary Fig. 10**. The related revised text can be found in P16 of revised manuscript and P5 of revised supplementary file:

P16 of revised manuscript: Although the normalized resistance of the RuO_2/glass resistive patch increases at 800~900 °C (may be due to the phase transition of the glass matrix (**Supplementary Fig. 10**)), it still has negative resistance–temperature characteristics from 25~1000 °C, with a TCR of -95~-365 ppm/°C (**Figs. 3f** and **3g**).

P5 of revised supplementary file: Simultaneous thermal analysis (STA 449C, Netzsch Group, Germany) was employed to characterize the differential scanning calorimetry (DSC) of the glass powder from room temperature to 1100 °C at a heating rate of 5 °C/min.

Q7: The comparison with other commonly used resistive materials is misleading. Although RuO_2/glass exhibits favorable properties, it does not possess the lowest room-temperature TCR. Please include comparisons with low-TCR materials for a more balanced evaluation.

Answer: We thank the reviewer for this comment. For our requirements regarding resistive materials, the foremost criterion is that the material must exhibit excellent high-temperature resistance and oxidation resistance; otherwise, it cannot be applied in service environments. Therefore, our objective is to find resistive materials that maintain a low TCR over a broad temperature range while possessing outstanding thermostability. To our knowledge, current research on TCR properties has primarily focused on the low-temperature range (<400 °C), and no reports have yet addressed the high-temperature TCR (>800 °C) of materials. Although several resistive materials with lower TCR, such as Ta-Si-O[1], TiN_x[2], $Mn_3Ag_{(1-x)}Cu_xN$[3], TaN[4], and Mn_3NiN[5], exhibiting near-zero TCR ranging from 1.9 ppm/°C to 33.28 ppm/°C, have been reported, these nitrides or oxides cannot maintain thermal stability up to 1000 °C. Therefore, this manuscript compares the TCR of conventional resistive materials used in metasurfaces with that of RuO_2/glass.

Q8: As the aerogel composites were treated to become hydrophobic, please provide contact angle measurements before and after surface modification to support this claim.

Answer: We thank the reviewer for this comment. The contact angle of the alumina aerogel composites after hydrophobic treatment with water was ~ 129° (**Fig. 8b**), indicating excellent hydrophobicity. For alumina aerogel composites before hydrophobic treatment, water spread rapidly over the surface of the aerogel and then was absorbed into the aerogel due to capillary forces, so the contact angle cannot be tested. We provide a set of snapshots to confirm that the aerogel before hydrophobic

treatment is hydrophilic (**Fig. 8a**).

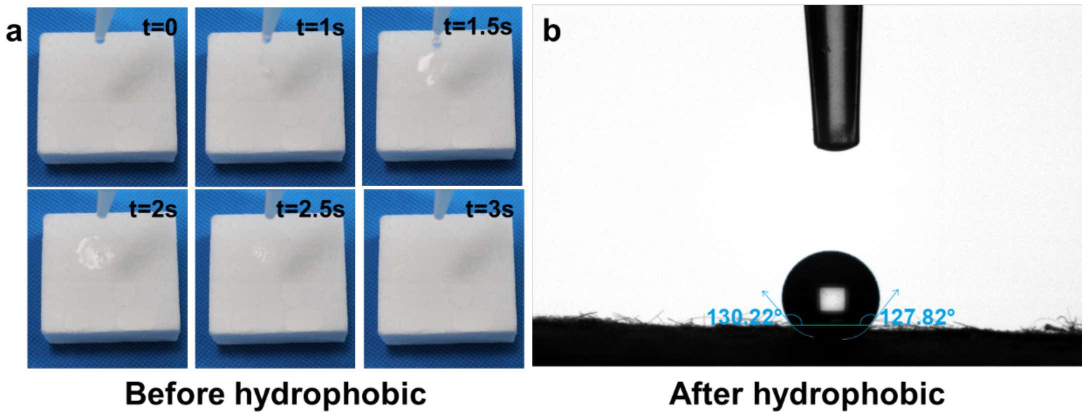

Fig. 8 Hydrophobicity test of alumina aerogels (a) before and (b) after hydrophobic treatment.

Revised version: Fig. 8 has been added to Supplementary file as **Supplementary Fig. 15**. The related revised text can be found in P5 of revised supplementary file:

The contact angle of alumina aerogel composites with water was measured using an optical tensiometer (Theta Flex, Biolin Scientific, Sweden) with sample dimensions of 20 mm × 20 mm × 10 mm.

Q9: While the load-bearing capability of the MTL-integrated composites is promising, fatigue resistance under cyclic loading and the temperature dependence of mechanical properties are critical for real applications. Please consider including such measurements.

Answer: We thank the reviewer for this comment. We evaluated the fatigue resistance of the MTL integrated composites through 100 compression cycles under a strain of ~5% (**Fig. 9a**). After ~ 20 loading–unloading cycles, the height of the MTL integrated composite decreased by only ~ 1.3% and remained nearly unchanged

during the subsequent 80 compression cycles (**Fig. 9b**). Moreover, the maximum compressive stress of each cycle remained nearly constant in the 100 cycles (**Fig. 9c**), demonstrating the excellent fatigue resistance of the MTL integrated composite.

We then investigated the high-temperature compressive properties of the MTL integrated composites (**Fig. 9e**). Under 8% strain, as the temperature increased from 400 °C to 1000 °C, the residual strain of the composites increased from 0.7% to 4% (**Fig. 9d**), indicating the occurrence of plastic deformation in the alumina aerogel composites. Compared with its elastic deformation of 10% at room temperature, the maximum elastic deformation of the aerogel decreased at elevated temperatures. The peak stress increased from 0.64 MPa to 0.73 MPa and the curve slope also increased, demonstrating the increased compressive resistance and compressive modulus of the aerogel (**Fig. 9d**). The increase in plastic deformation and compressive modulus collectively led to an obvious expansion in the hysteresis loop area of the loading–unloading curves, suggesting the enhanced energy dissipation efficiency and damping capabilities of the aerogel (**Fig. 9d**). In summary, higher temperatures led to reduced elastic deformation, increased modulus, and improved damping performance of the MTL integrated composites.

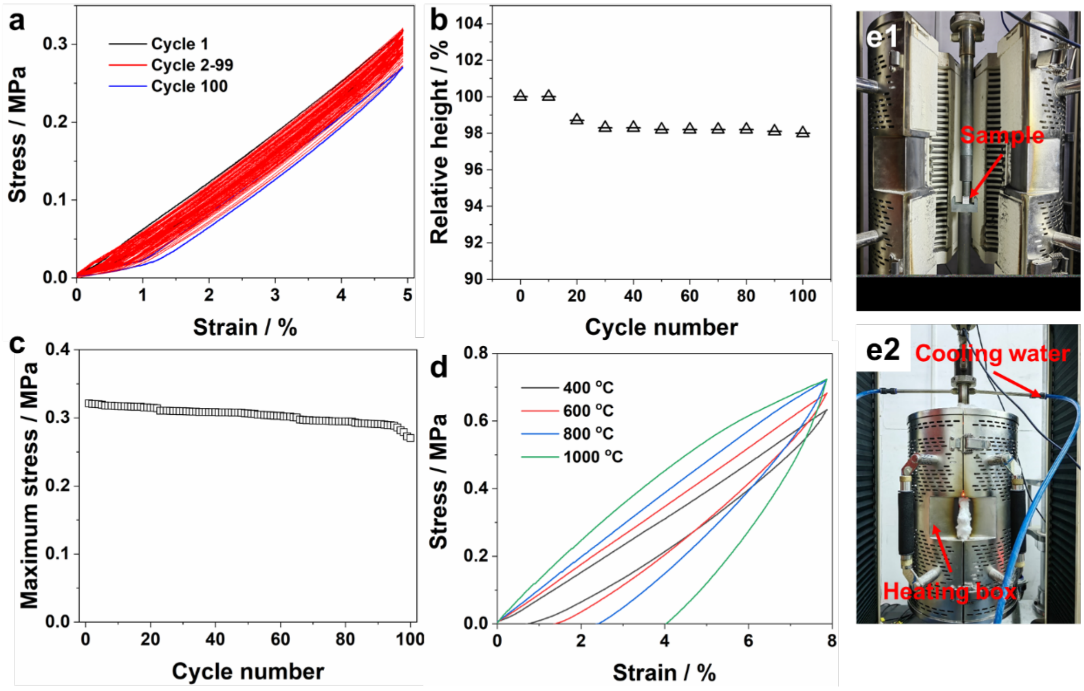

Fig. 9 (a) Stress–strain curves, (b) relative height, and (c) maximum stress of 100 compression cycles of the MTL integrated composites at 5% strain. (d) Compressive stress–strain curves of the MTL integrated composites at 8% strain at 400 °C, 600 °C, 800 °C, and 1000 °C. (e) Testing equipment for determining high-temperature compression properties.

Revised version: Fig. 9 has been added to Supplementary file as **Supplementary Fig. 23**. The related revised text can be found in P25 of revised manuscript and P6 of revised supplementary file:

P25 of revised manuscript: Moreover, fatigue resistance under cyclic loading and the temperature dependence of mechanical properties are critical for practical applications. The compressive loading-unloading tests demonstrate the durable cyclic performance of the MTL integrated composite at 5% strain (**Supplementary Fig. 23a**). After 100 compression cycles, the relative height decreased by only 1.3%, while the maximum stress remained nearly constant (**Supplementary Fig. 23b** and **23c**).

The temperature-dependent compressive cycling tests revealed that the MTL integrated composite exhibits an increased compressive modulus, enhanced damping capacity, and reduced maximum elastic strain (**Supplementary Fig. 23d** and **23e**).

P6 of revised supplementary file: ***High-temperature compressive property***: High-temperature compression tests were conducted on bars with dimensions of 15 mm × 15 mm × 18.1 mm using a WDW-50 universal testing machine at a load rate of 2 mm/min. A heating chamber encloses the sample and indenter. The protruding section of the indenter outside the chamber is connected to a cooling water supply. The test is initiated after the temperature stabilizes at the target value and is maintained for 10 min.

Q10: Given the inherent brittleness of glass, what is the fracture toughness of the RuO_2/glass resistive materials? Please discuss how mechanical damage or microcracking might influence the overall performance and reliability of the MTL-integrated composite.

Answer: We thank the reviewer for this comment. The sintering process of the RuO_2/glass resistor pastes is accompanied by volatilization and burnout of organic solution, which leads to many bubbles and pores when preparing bulk composites. Therefore, the RuO_2/glass composites are difficult to prepare as bulk materials to test their fracture toughness. We designed a bending test to examine the damage resistance of the RuO_2/glass patches. The resistor paste was printed onto an ultra-thin Al_2O_{3f}/Al_2O_3 substrate, which was then wrapped around a cylindrical surface with diameter of 40 mm (**Fig. 10a**). Subsequently, the patches and substrate were unwound

(**Fig. 10b**). Throughout this process, no cracks appeared on the surface of the patches, and their sheet resistivity only changed 0.83% (inset of **Fig. 10a**). Although glass is inherently brittle, the RuO_2/glass resistive patch exhibits remarkable flexibility due to its extremely thin thickness (10~20 μm), which results in a plane stress state. This means that the resistive patch can deform freely along the *z*-direction while maintaining $\sigma_z \approx 0$.

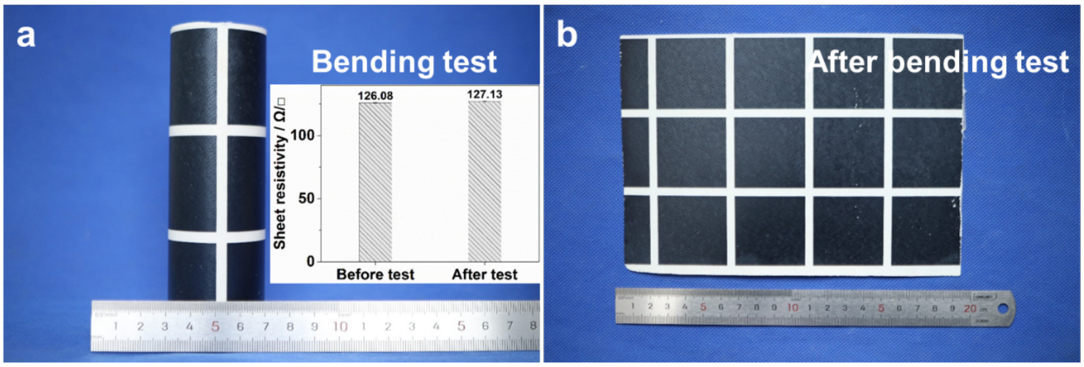

Fig. 10 The picture of the RuO_2/glass metasurface (a) in and (b) after the bending test. Inset of (a): sheet resistivity change rate of the patches before and after bending test.

The Al_2O_{3f}/Al_2O_3 ceramic composites and alumina aerogel composites are mainly responsible for the load-bearing ability of the MTL integrated composites. The metasurface layers are not used as the load-bearing unit because of their very small thickness. The natural advantages of continuous fiber-reinforced Al_2O_{3f}/Al_2O_3 ceramic composites or long fiber-reinforced alumina aerogel composites include high damage tolerance and low crack sensitivity. In particular, the fracture toughness of the Al_2O_{3f}/Al_2O_3 ceramic composites measured by the single edge notched beam (SENB) method was >8 MPa·m$^{1/2}$. For alumina aerogel composites, cracks are more difficult to propagate because of the lack of a matrix. Therefore, defects such as microcracks or micropores have little effect on the overall performance and reliability of

MTL-integrated composites.

Revised version: Fig. 10 has been added to Supplementary file as **Supplementary Fig. 14**. The related revised text can be found in P17 of revised manuscript:

Notably, the RuO_2/glass metasurface exhibits excellent mechanical flexibility with its sheet resistivity remaining nearly unchanged before and after bending (**Supplementary Fig. 14**).

Q11: Regarding the mechanical properties part: it is unclear how the thermal insulation and electromagnetic wave absorption change under a stressed (load-bearing) condition. Is the bandwidth of electromagnetic wave absorption related to the microstructure of the aerogel?

Answer: We thank the reviewer for this comment. Owing to limitations in experimental conditions, the thermal insulation and microwave-absorbing properties of the MTL integrated composite in the load-bearing state are difficult to measure. Therefore, we used a simulation method to address this issue. Assuming that the MTL integrated composite is under compressive stress, the thickness of the alumina aerogel composite decreases, and the thermal conductivity and dielectric constant slightly change. For thermal simulations, only the effect of thickness variation is considered. Finite element simulations were used to analyze the backside temperature of the MTL integrated composite under one-sided heating (**Fig. 11a**). When the aerogel thickness was reduced by 0%, 5% and 10% (the maximum elastic deformation of the aerogel), the backside temperature of the MTL integrated composite increased slightly from

123 °C to 134 °C and 146 °C, respectively. The simulation results demonstrate that the thermal insulation performance of the MTL integrated composite exhibits very little change within the elastic deformation range of the alumina aerogel composite.

For electromagnetic simulations, the simultaneous variations in both thickness and ε' were considered. Based on the measurements, the reduction in aerogel thickness is nearly proportional to the increase in its ε'. Therefore, the reflectivity of the MTL integrated composite was simulated when the aerogel thickness and ε' changed by 2%, 5%, and 10%. As the decreases of aerogel thickness and increase of aerogel ε', the dual resonance peaks gradually shift toward high frequencies, resulting in a gradual deterioration in the low-frequency absorption performance (**Fig. 11b**). Under the maximum elastic deformation (10%) of the alumina aerogel composite, the effective absorption bandwidth (EAB) of the MTL integrated composite only decreased by 1% (from 10 GHz to 9.9 GHz). Therefore, the microwave absorption performance of the MTL integrated composite exhibits very slight variation under load-bearing conditions.

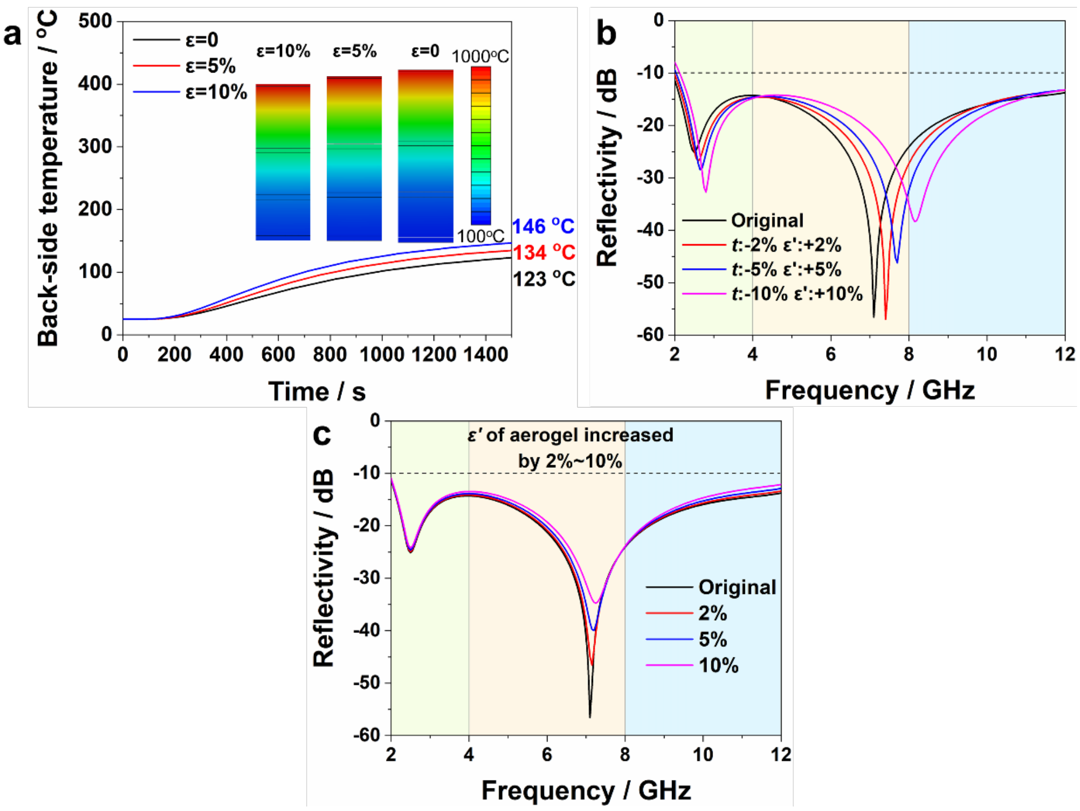

Fig. 11 The simulated (a) back-side temperature and reflectivity of the MTL integrated composites under different (b) aerogel compression strains and (c) aerogel dielectric constant.

For the second question, according to the input conditions of the aerogel in electromagnetic simulations, the EAB is primarily determined by its thickness, ε', and $\tan\delta$. Therefore, regardless of the microstructure of the aerogel, the microwave absorption performance of the MTL integrated composite can remain unchanged as long as its ε' and $\tan\delta$ remain constant. Under compression, the aerogel ε' increases while the $\tan\delta$ remained nearly unchanged. We simulated the reflectivity of the MTL integrated composite (**Fig. 11c**) when ε' of the aerogel increased by 2%, 5%, and 10%. As the increase of ε', the positions of the dual resonance peaks remain almost unchanged. However, the absorption intensity of the high-frequency resonance peak

weakens from -56 dB to -34 dB. Furthermore, variations of 10% in aerogel ε' have no significant effect on the EAB of the MTL integrated composites within the 2~12 GHz.

Revised version: Fig. 11c has been added to Supplementary file as **Supplementary Fig. 3a**. The related revised text can be found in P9 of revised manuscript:

It is worth noting that the EAB of this microwave-absorbing structure exhibits high robustness to these structural parameters (**Supplementary Fig. 3**), implying that fabrication tolerances are allowed.

Q12: The impact of the supercritical drying process on the aerogel's pore structure is not adequately addressed. Please provide detailed pore size distribution data or SEM/TEM images to clarify this relationship.

Answer: We thank the reviewer for this comment. The preparation process of the alumina aerogel composite in this manuscript is similar to the process in the literature[6] (**Fig. 12**). To determine the impact of supercritical drying on the pore structure of aerogel composites, the pore structure or SEM images of the material before and after supercritical drying should be obtained. However, before supercritical drying, the material exists as a wet gel with its pores filled by sol. The wet gels cannot undergo mercury intrusion porosimetry (MIP), Brunauer–Emmett–Teller (BET) testing, or SEM characterization. Therefore, we apologize that it is difficult to provide the pore structure of the aerogel before supercritical drying. The morphologies of the aerogel after supercritical drying are shown in **Fig. 12b** and **12c**.

Ethanol supercritical drying is a very mature preparation process for aerogels.

We provide a theoretical explanation for why supercritical drying would not destroy the pore structure of aerogels. When the temperature and pressure of ethanol exceed its critical point (243 °C, 6.3 MPa), the gas–liquid interface disappears, and an ethanol supercritical fluid is formed. At this stage, the supercritical fluid simultaneously exhibits high diffusivity and strong solvation capacity with zero surface tension. The supercritical fluid rapidly diffuses into the pores of the material to dissolve and remove the liquid ethanol. Moreover, the absence of surface tension does not damage the pore structure of the aerogel. While the supercritical temperature is maintained, the pressure is gradually reduced to convert the supercritical fluid into gas (avoiding the re-emergence of surface tension during phase transition). Finally, the ethanol gas is purged through ventilation, and the aerogel is obtained. Thus, on the basis of the above principles, ethanol supercritical drying does not damage the pore structure of the aerogel.

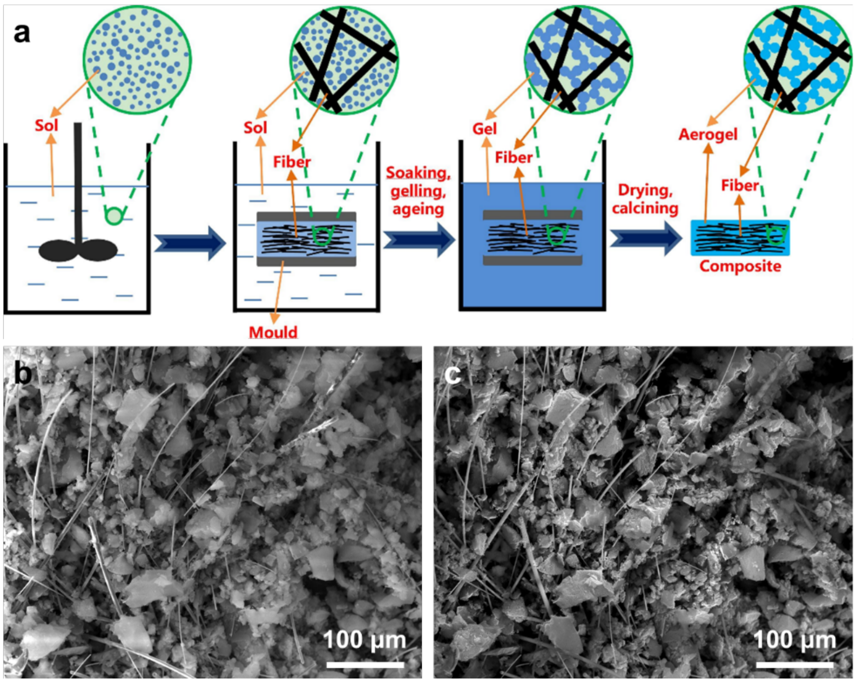

Fig. 12 (a) Schematic diagram of the preparation process of the alumina aerogel

composites[6]. (b) SEM and (c) BSE images of the alumina aerogel composites after supercritical drying.

Revised version: Fig. 12b and 12c has been added to Supplementary file as **Supplementary Fig. 16**. The related revised text can be found in P17 of revised manuscript:

The aerogel composites were hydrophobized (**Supplementary Fig. 15**) to protect the pore structure **(Supplementary Fig. 16)** from destroying during the subsequent infiltration of alumina sol that is harmful to their low dielectric characteristic and heat insulation performance.

Q13: Since the performance of aerogels can be significantly affected by environmental conditions, particularly humidity, please evaluate how the MTL-integrated composites behave under varying humidity levels or prolonged moisture exposure. Such data would improve understanding of their long-term stability and functionality.

Answer: We thank the reviewer for this comment. The MTL integrated composite was placed in a constant temperature and humidity chamber (**Fig. 13b**) and maintained at 95% relative humidity for 12 h before reflectivity measurement. The EAB and resonant peak positions of the MTL integrated composite remained almost unchanged before and after the humidity treatment (**Fig. 13a**), demonstrating its excellent moisture resistance. This stability is due to the hydrophobicity of the alumina aerogel composites, which imparts superior resistance to humid environments.

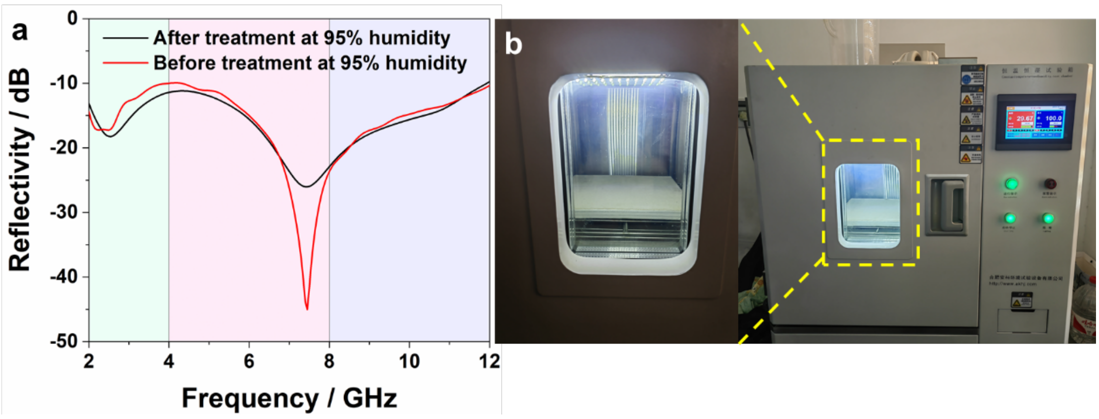

Fig. 13 (a) Reflectivity of the MTL integrated composites before and after treatment at 95% humidity for 12 h. (b) Picture of a sample in a constant-temperature and constant-humidity test chamber.

Revised version: Fig. 13 has been added to Supplementary file as **Supplementary Fig. 20**. The related revised text can be found in P21 of revised manuscript:

Moreover, high-humidity conditions often present additional challenges in extreme environments, placing further demands on the long-term stability of MTL integrated composites. The humidity resistance of MTL integrated composites was evaluated (**Supplementary Fig. 20**) and its EAB remained unchanged after prolonged moisture exposure (95% humidity for 12 h), indicating excellent stability against humidity.

Minor comments:

1. Most equations are presented without definitions of variables or references, which makes them difficult to interpret. Please ensure that all variables are defined and cited appropriately.

Answer: We thank the reviewer for this comment. We have annotated the

physical meanings of the variables in Equations 1~3 in the manuscript. The revised manuscript is as follows:

$$Z_n = R_n + j\left(\omega L_n - \frac{1}{\omega C_n}\right) \tag{1}$$

where Z_n, R_n, L_n, and C_n (n = 1, 2. 1 and 2 represent the top and lower metasurfaces, respectively) represent the equivalent impedance, real part of the impedance, equivalent inductance, and equivalent capacitance of the metasurface, j represents the imaginary unit, and ω represents the angular frequency.

$$Z_{in1} = jZ_0\sqrt{\frac{1}{\varepsilon_{r1}}}\tan\left(\frac{\omega}{c}\sqrt{\varepsilon_{r1}}d_1\right) \tag{2}$$

where Z_{in1}, ε_{r1}, and d_1 represent the input impedance, dielectric constant, and thickness of the lower-layer Al_2O_{3f}/Al_2O_3 ceramic composites, Z_0 is the wave impedance of free space ($Z_0 \approx 377\ \Omega$), and c is the speed of light in vacuum.

$$Z_{in} = Z_{s1}\frac{Z_{in6} + jZ_{s1}\tan(\frac{\omega}{c}\sqrt{\varepsilon_{r1}}d_5)}{Z_{s1} + jZ_{in6}\tan(\frac{\omega}{c}\sqrt{\varepsilon_{r1}}d_5)} \tag{3}$$

where Z_{in} is the input impedance of the MTL integrated composites, Z_{in6} is the input impedance of the MTL integrated composites without top layer Al_2O_{3f}/Al_2O_3 ceramic composites, Z_{s1} is the wave impedance of the Al_2O_{3f}/Al_2O_3 ceramic composites, and d_5 is the thickness of the top-layer Al_2O_{3f}/Al_2O_3 ceramic composites.

Additionally, we identified an error in **Fig. 2a** of the manuscript, where the order of $d_1 \sim d_5$ was mistakenly reversed. This has been corrected in the revised manuscript.

2. Please provide the thermal conductivity values of the MTL-integrated composites and compare them with those of state-of-the-art thermal insulation

materials.

Answer: We thank the reviewer for this comment. We tested the thermal conductivity of the MTL integrated composites (**Table 3**). The thermal conductivity of the MTL integrated composites is 38 mW/(m·K) at room temperature and 143 mW/(m·K) at 1000 °C. Compared with other aerogels reported in the literature, the room-temperature thermal conductivity of the MTL integrated composites in this work is very low, only higher than that of 26 mW/(m·K) for zircon nanofibrous aerogels (ZAGs), 20 mW/(m·K) for BN aerogels, and 30 mW/(m·K) for glass fiber aerogels (**Fig. 14a**). More importantly, the MTL integrated composites exhibit ultra-low high-temperature thermal conductivity. To our knowledge, ZAGs currently have the lowest reported thermal conductivity at 1000 °C (104 mW/(m·K))[7]. Other aerogels (e.g., BN aerogels, ZrO_2 aerogels, SiO_2 aerogels, Al_2SiO_5 aerogels, and asbestos aerogels) typically demonstrate thermal conductivities exceeding 200 mW/(m·K) at 1000 °C (**Fig. 14b**)[7]. The remarkably low thermal conductivity of the MTL integrated composite implies its exceptional thermal insulation performance.

Table 3 Thermal conductivity of the MTL integrated composites at room temperature and 1000 °C

Temperature (°C)	Method	Test criteria	Sample size (mm)	Thermal conductivity (mW/(m·K))
25	Heat Flow Meter Method	GB/T 10295-2008	300 × 300 × 18	38
1000	Water Flow Plate Method	YB/T 4130-2005	Φ180 × 18	143

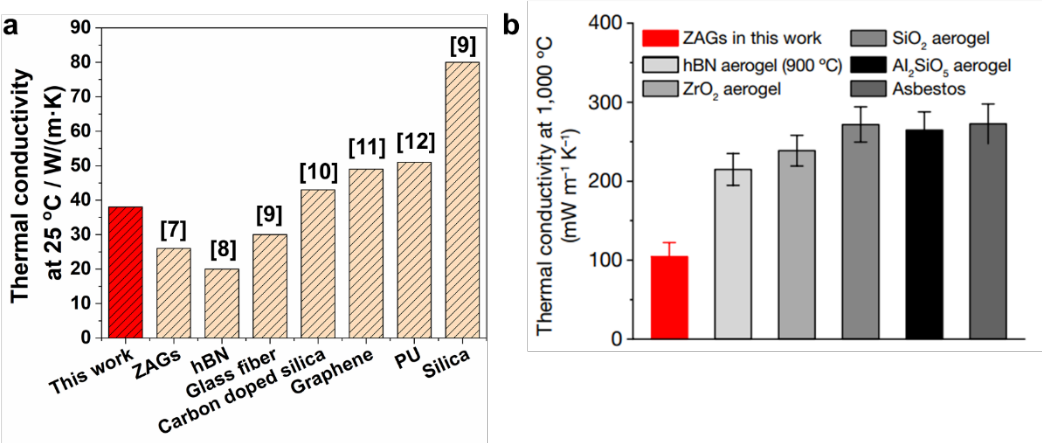

Fig. 14 (a) Comparison of the thermal conductivities of the alumina aerogel composites with those of other aerogels at 25 °C[7, 8, 9, 10, 11, 12]. (b) Thermal conductivity of other aerogels at 1000 °C (the picture is from reference[7]).

Revised version: Table 3 and Fig. 14 has been added to Supplementary file as **Supplementary Table 4** and **Supplementary Fig. 21**. The related revised text can be found in P22 and P30 of revised manuscript:

P22: According to steady-state measurements, the MTL integrated composite exhibits ultra-low thermal conductivity at room temperature (38 mW/(m·K)) and 1000 °C (143 mW/(m·K)) (**Supplementary Table 4**), which is comparable to state-of-the-art thermal insulation materials such as ZAGs aerogel or *h*-BN aerogel (**Supplementary Fig. 21**).

P30: The room-temperature thermal conductivity of alumina aerogel composites was tested using a thermal conductivity tester (DRPL-III, Xiangtan Xiangyi Instrument CO., LTD., China) based on heat flow meter method with sample size of 300 mm × 300 mm × 18 mm. The thermal conductivity of alumina aerogel composites at 1000 °C was tested using a high-temperature thermal conductivity tester (DRS-III, Xiangtan Xiangyi Instrument CO., LTD., China) based on water flow

plate method with sample size of Φ180 mm × 18 mm.

Responses to reviewer #2:

We are deeply honored and profoundly grateful for your exceptionally positive assessment of our manuscript and your recommendation for immediate acceptance. Your thorough, insightful review not only validates our work but also reflects your remarkable expertise and generosity. We are sincerely humbled by your supportive feedback and the invaluable time you dedicated to evaluating our research. Your recognition means a great deal to us and significantly inspires our future endeavors. Thank you for your exceedingly kind and constructive contribution to our work.

Responses to reviewer #3:

We are deeply grateful to the reviewer for the insightful and constructive suggestions on tunneling effects and electromagnetic model. These recommendations have been very valuable in deepening our comprehension of tunneling, resonance, destructive interference, and equivalent circuit analysis, and will greatly improve the quality of the revised manuscript.

Q1: Lack of Direct Evidence for the Tunneling Mechanism: The central claim of the manuscript is that quantum tunneling across nanoscale RuO_2-glass interfaces is responsible for the ultra-low TCR (~ –95 to –365 ppm/°C). However, no direct electrical measurements (e.g., temperature-dependent I–V characteristics, non-linear conduction analysis, or Fowler–Nordheim plots) are provided. The model presented

for the barrier and bulk resistance is theoretical and not supported by quantitative fitting. Given that the tunneling hypothesis is a central novelty claim, its current support is insufficient for a high-impact journal.

Answer: We thank the reviewer for this comment. This is an excellent experimental suggestion that will contribute to advancing our understanding of the tunneling effect. RuO_2/glass resistive patches were fabricated and their current-voltage (I-V) characteristics (**Fig. 1a**) at various temperatures were measured using a four-probe system. Furthermore, Fowler-Nordheim (F-N) plots (**Fig. 1b**) for the RuO_2/glass patches at different temperatures were constructed. At all tested temperatures, $\ln(I/V^2)$ versus 1/V exhibited clear linearity, indicating that F-N tunneling dominates the electron transport. Notably, the intercept of the F-N plot at 800 °C was significantly reduced. This may be attributed to the phase transition of glass inducing a higher work function, which hinders electron emission.

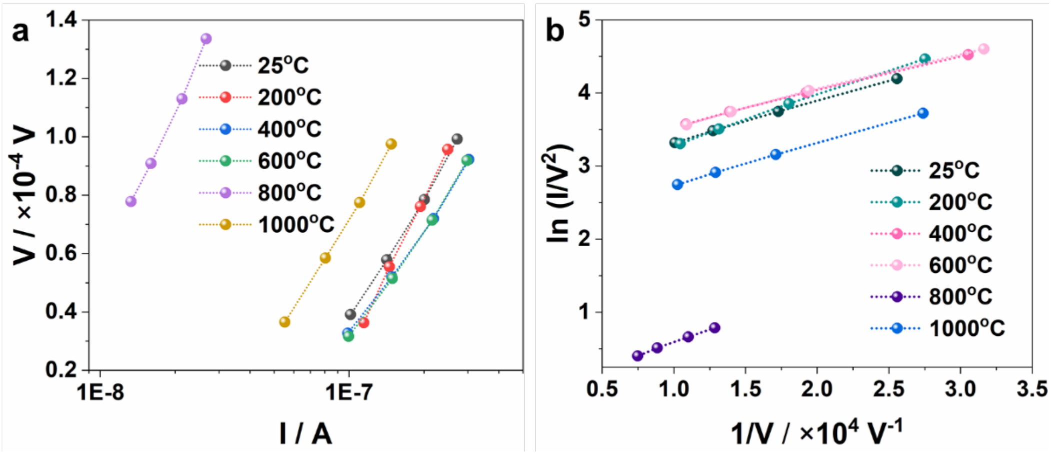

Fig. 1 (a) I-V plots and (b) Fowler–Nordheim plots of RuO_2/glass resistive patch at different temperatures.

Revised version: Fig. 1a has been added to Supplementary file as **Supplementary Fig. 10b**. Fig. 1b has been added to revised manuscript as **Fig. 3e**.

Fig. 3e in original manuscript has been moved to Supplementary file as **Supplementary Fig. 10a**. The related revised text can be found in P14&P15 of revised manuscript and P6 of revised supplementary file:

P14 of revised manuscript: The temperature-dependent current-voltage (I-V) characteristics (**Supplementary Fig. 8b**) and the linear Fowler-Nordheim (F-N) plot ($\ln(I/V^2)$ versus 1/V) (**Fig. 3e**) confirm that the F-N tunneling dominates the electron transport mechanisms of the resistive patch from room temperature to 1000 °C. According to **Eqs. (4)** and **(5)**, $R_b(T)$ has negative resistance–temperature characteristics, whereas $R_m(T)$ has positive resistance–temperature characteristics. Thus, on one hand, the tunneling mechanism is insensitive to temperature; on the other hand, $R_b(T)$ and $R_m(T)$ exhibit opposite TCR. Both factors suggest that the RuO_2/glass may show an extremely low TCR.

P15 of revised manuscript: (e) Fowler-Nordheim plots of RuO_2/glass resistive patch at different temperatures.

P6 of revised supplementary file: The RuO_2/glass resistor paste was printed and sintered on an alumina ceramic substrate with dimensions of 15 mm × 4 mm × 1 mm. The sample was then used to tested the current-voltage characteristic at 25~1000 °C using the high-temperature four probe system.

Q2: Inadequate Electromagnetic Modelling: The use of HFSS and equivalent circuit modelling is a good start, but the EM response lacks deeper analysis. There is no modal decomposition, no mapping of current loss densities across layers, nor is there evidence of destructive or constructive interference effects. The two-resonance

explanation (S- and X-band) is based on electric field snapshots but does not rigorously prove the mechanism of field coupling between layers. A more complete EM loss analysis is necessary to substantiate the absorber's behavior. While the top and bottom metasurfaces share the same sheet resistance but differ in mesh geometry, no analysis is provided on the distinct roles of inductive versus capacitive responses. Is there any evidence for trapped-mode resonances arising from the dual-metasurface configuration?

Answer: We thank the reviewer for this comment. We sincerely apologize that the aerogel thickness in the original Supplementary Fig. 2 was incorrectly provided due to an oversight. The updated thickness parameters, corresponding reflectivity curve, and Smith chart can be found in Supplementary Fig. 2, Fig. 2e, and Fig. 2c, respectively. Now, we respond to these questions one by one.

(1) Modal decomposition: To investigate the modal of electric or magnetic resonance, we simulated the surface current distributions of the dual-metasurface and the metal backplane (**Fig. 2**). At low frequency, the currents in each layer flow in the same direction, leading to electric resonance between the electromagnetic wave and the structure, with electromagnetic energy loss mainly occurring in the lower metasurface. At high frequency, the surface currents in the dual-metasurface remain co-directional, resulting in electric resonance, while the surface currents in the metal backplane and the lower metasurface flow in opposite directions, forming a magnetic resonance loop and thus exciting magnetic resonance. Meanwhile, electromagnetic energy loss at high frequency is primarily produced in upper metasurface.

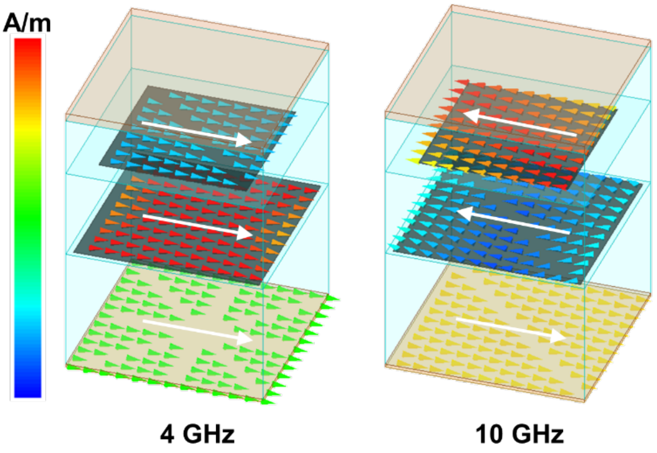

Fig. 2 Surface current distribution of the dual-metasurface and metal backplane at 4 GHz and 10 GHz.

(2) Mapping of current loss densities across layers: The Fig. 2e (now is Fig. 2g) provided in our original manuscript represents the current loss density plot. We sincerely apologize for mistakenly labeling it as the electric field plot in the previous version. The relevant descriptions have been corrected in the revised manuscript (P10 and P12). Notably, induced currents only exist within the metasurface layer, while no induced currents are observed in either the alumina aerogel or Al_2O_{3f}/Al_2O_3 composite layers.

(3) Evidence of destructive or constructive interference effects: The phase and amplitude of the MTL integrated composite within the 2~12 GHz are shown in **Fig. 3**. The phase reversal and zero amplitude at 7.1 GHz provide clear evidence of destructive interference of EM waves. The amplitude minimum at 2.5 GHz also indicates strong interference, corresponding to the resonance peak in S-band.

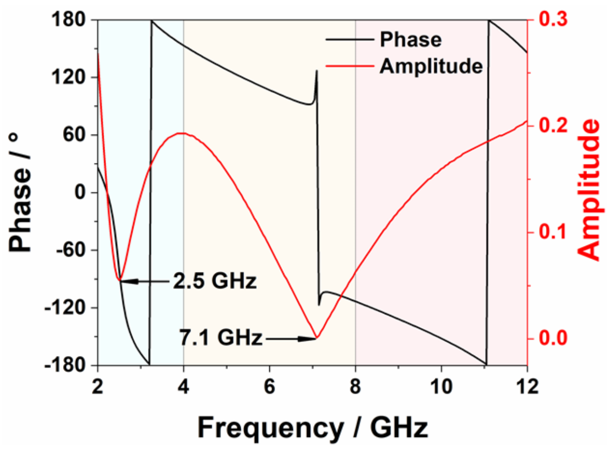

Fig. 3 Phase and amplitude of microwave in the MTL integrated composites at 2~12 GHz.

(4) Rigorously proof of the two-resonance explanation: The formation of the dual-resonance is primarily due to the destructive interference. As shown in **Fig. 3**, the phase reversal and zero amplitude at 7.1 GHz provide clear evidence of destructive interference of EM waves. Then, the EM energy is absorbed by the metasurfaces, which significantly reduces the EM wave reflectivity, thereby forming a resonance peak. The amplitude minimum at 2.5 GHz also indicates strong interference, corresponding to the resonance peak in S-band. On the other hand, the frequency-dependent power loss contributions of the dual-metasurface structure was calculated (**Fig. 4**). As frequency increases, the loss contribution from the lower metasurface gradually decreases, while that from the upper metasurface gradually increases. This trend is consistent with the conclusion presented in Fig. 2e of the manuscript. The lower metasurface is primarily responsible for absorption in S-band, whereas the upper metasurface is primarily responsible for absorption in X-band.

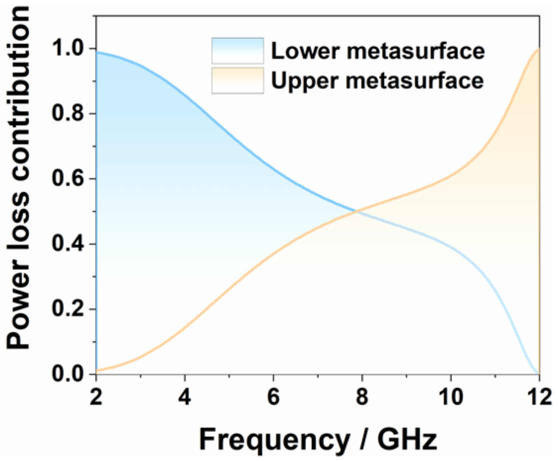

Fig. 4 Frequency–dependent power loss contribution of the dual-metasurface.

(5) Analysis of the distinct roles of inductive versus capacitive responses: The role of equivalent capacitance and inductance is to introduce an imaginary component into the input impedance of the metasurface, thereby canceling out the imaginary part of the impedance of dielectric layer and achieving impedance matching with free space. For the square patch metasurface structure (**Fig. 5a**), electrons on the patches are confined to move within the length of their respective patch. When electromagnetic wave is incident, driven by the electric field direction, most positive and negative charges separate and accumulate on opposite edges of each patch. Consequently, adjacent patches develop opposite polarities on their facing edges. Therefore, the gaps between adjacent units, oriented perpendicular to the electric field direction, can be modeled as capacitive elements. The patches themselves, oriented parallel to the electric field, exhibit inductive behavior. However, due to its square shape, the equivalent inductance of the patch becomes negligibly weak and can be omitted.

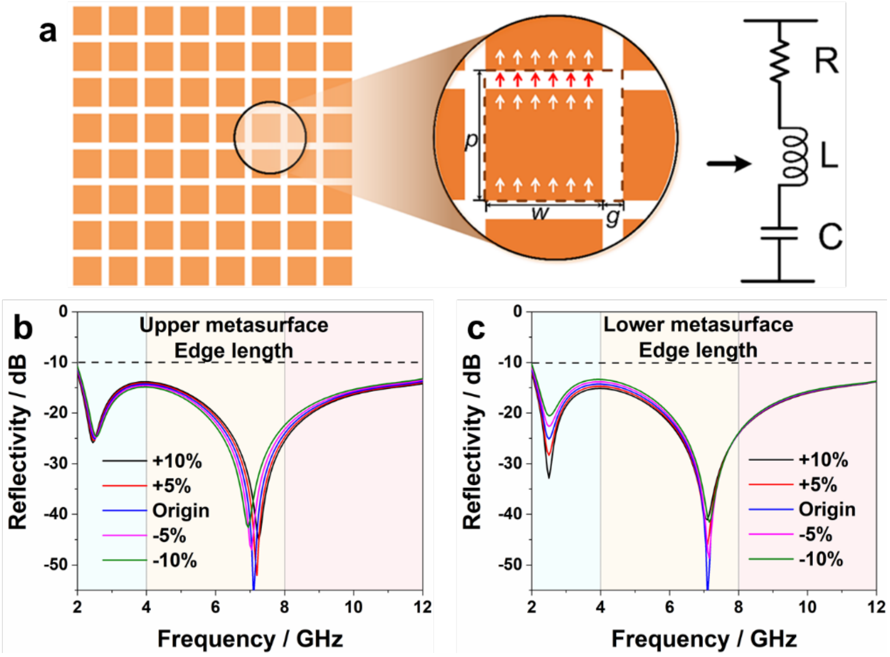

Fig. 5 (a) Schematic diagram of the equivalent mechanism of circuit parameters corresponding to the square patch metasurface. (b, c) Simulated reflectivity of MTL integrated composites when edge length of RuO_2/glass resistive patch change in range of ±10%.

Therefore, we conducted simulation analysis to investigate the influence of equivalent capacitance on absorption performance of the MTL integrated composites. Since the equivalent capacitance can be controlled by the edge length of the patches, we varied the edge length to illustrate its effect on absorption performance. As shown in **Figs. 5b** and **5c**, when the edge lengths of the upper and lower patches changed within ±10%, the EAB of the MTL integrated composite remained at 10 GHz (2~12 GHz). Furthermore, the number and positions of the resonance peaks kept almost unchanged. Therefore, our findings suggest that the absorption performance of the MTL integrated composite is relatively robust against variations in the edge length of patches.

(6) Evidence for trapped-mode resonances arising from the dual-metasurface configuration: According to the impedance matching principle, the normalized input impedance with real part = 1 and imaginary part = 0 represents the best impedance matching. At the optimal impedance matching point, strong microwave absorption occurs, which corresponds to the resonant peak in the reflectivity curve. As shown in **Fig. 6**, the ideal matching point were designed at 7.1 GHz that its Re(Z_{in}) is equal to 0 and Im(Z_{in}) is equal to 0, which corresponds to the resonant peak in C band in Fig. 2d of the manuscript. In S band, its optimal matching point was designed at 2.5 GHz with Re(Z_{in}) = 1 and Im(Zin) = -0.11, corresponding to the resonant peak in S band in Fig. 2d of the manuscript. In summary, the dual-resonant point represents the optimal design; otherwise, the EAB could not cover the 2~12 GHz range.

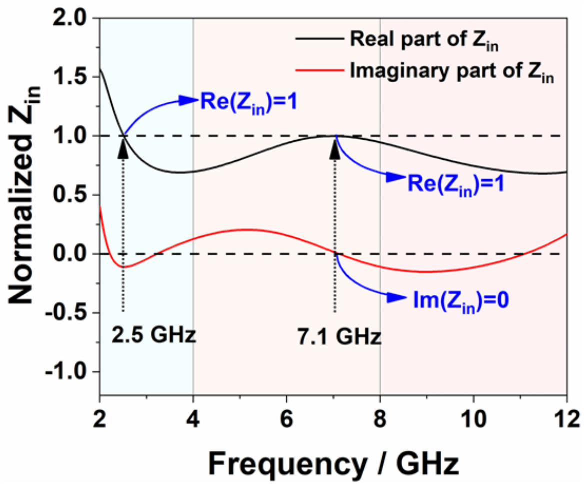

Fig. 6 The real part and imaginary part of normalized Z_{in} of the MTL integrated composites at 2~12 GHz.

Revised version: Fig. 3, Fig. 4, and Fig. 6 has been added to revised manuscript as **Fig. 2f**, **Fig. 2h**, and **Fig. 2d**. Fig. 5b and Fig. 5c has been added to Supplementary file as **Supplementary Fig. 3c** and **Supplementary Fig. 3d.** The related revised text

can be found in P9, P11, P12 of revised manuscript:

P9 (**Supplementary Fig. 3c, Supplementary Fig. 3d**): It is worth noting that the EAB of this microwave-absorbing structure exhibits high robustness to these structural parameters (**Supplementary Fig. 3**), implying that fabrication tolerances are allowed.

P11 (**Fig. 2f**): The formation of the dual-resonance is primarily due to destructive interference. The phase reversal and zero amplitude at 7.1 GHz (**Fig. 2f**) provide clear evidence of destructive interference of EM waves. Then, the EM energy is absorbed by the metasurfaces, which significantly reduces the EM wave reflectivity. The amplitude minimum at 2.5 GHz (**Fig. 2f**) also indicates strong interference, corresponding to the resonance peak in S-band.

P11 (**Fig. 2d**): Specifically, the impedance trace of the dual-layer microwave-absorbing structure passes through the ideal matching point, which is further confirmed by its normalized input impedance with a real part of 1 and an imaginary part of 0 at 7.1 GHz (**Fig. 2d**).

P12 (**Fig. 2h**): The frequency-dependent loss contribution curves (**Fig. 2h**) of the dual-metasurface further confirm this EM response mechanism.

Q3: Equivalent Circuit Model and Input Impedance Matching: The proposed RLC ladder circuit model is mathematically defined from Z_1 to Z_6, but the physical correlation between each subcircuit and specific absorption features is unclear. Has this model been experimentally validated, and what is the quantitative error between simulation and measurement across the 2~12 GHz range? The model assumes ideal

dielectric behavior for intermediate layers. However, aerogel composites exhibit strong frequency-dependent permittivity (ε') and dielectric loss ($\tan\delta$), particularly from 25 to 1000 °C. Has any sensitivity analysis been conducted to quantify how these variations affect impedance matching and absorption?

Answer: We thank the reviewer for this comment. We respond to these questions one by one.

(1) Physical correlation between each sub-circuit and specific absorption features: The absorption features of the composites are determined by its input impedance as shown in **Equation 1**:

$$r = \frac{Z_{in} - Z_0}{Z_{in} + Z_0} \tag{1}$$

where r is the reflection coefficient, Z_{in} is input impedance of the composites, and Z_0 is the impedance of air ($Z_0 \approx 377\ \Omega$). Based on the equivalent circuit model (Fig. 2b in manuscript), a mathematical relationship between Z_{in} of MTL integrated composite and the input impedances of its individual sub-circuits (Z_{in1} ~ Z_{in6}) was previously defined. To clarify the physical connection between Z_{in} and Z_{in1} ~ Z_{in6}, their frequency-dependent curves are provided (**Fig. 7**). According to impedance matching theory, the real and imaginary parts of the normalized impedance should approach 1 and 0, respectively. **Fig. 7** illustrates the step-by-step optimization process of both the real parts (**Fig. 7a**) and imaginary parts (**Fig. 7b**) of the impedance, demonstrating the contribution of each material layer to the overall input impedance. Overall, the input impedance of the MTL integrated composite results from the co-optimization of all seven material layers. Each layer contributes specifically to the total input impedance,

as reflected in the changing trends of the curves shown in **Fig. 7**.

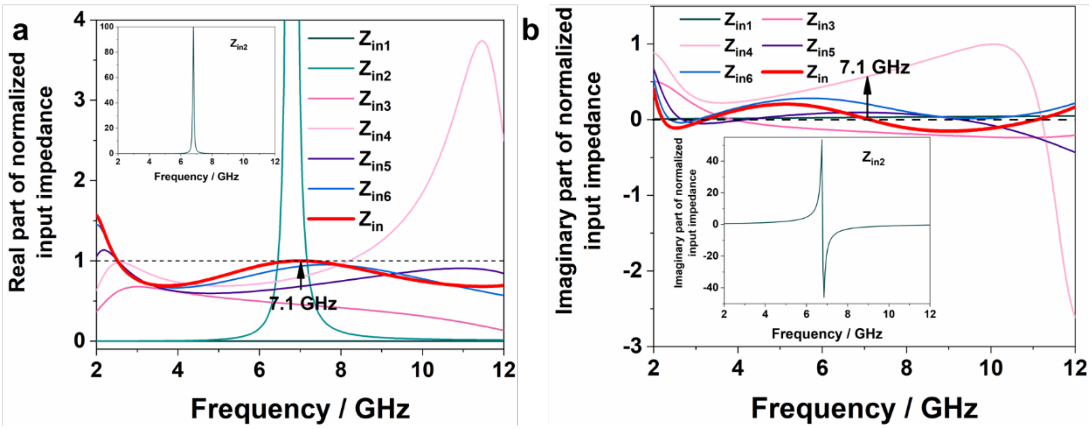

Fig. 7 (a) Real part and (b) imaginary part of Z_{in1}~Z_{in6} and Z_{in}.

(2) The validity of the model: This circuit model is validated experimentally. As shown by the measured and simulated reflectivity curves in **Fig. 8a**, both exhibit an identical EAB of 10 GHz. The number of resonance peaks and the tendency of curve are also consistent. The slight differences of the two curves likely arise from the inevitable deviations in material thickness and electromagnetic parameters from their optimal values.

(3) Sensitivity analysis of dielectric constant of the aerogel composites: In fact, as low dielectric materials, the ε' and tanδ of alumina aerogel composites and Al_2O_{3f}/Al_2O_3 ceramic composites changes very little in 2~12 GHz. As the increasing of temperature, the ε' increase by 2.2% (from 1.37 to 1.4) and tanδ increase by 3.8% (from 0.008 to 0.0083) show very little variation (**Supplementary Fig. 1**). To quantify the impact of ε' on the absorption performance, we simulated the reflectivity of the MTL integrated composites when the ε' of the aerogel increased by 2%, 5%, and 10% (**Fig. 8b**). As ε′ increases, the resonant peak and the EAB remained almost unchanged across 2~12 GHz. These results indicate that the absorption performance

of the MTL integrated composites is insensitive to changes in the ε' of the dielectric spacer layer within a certain range.

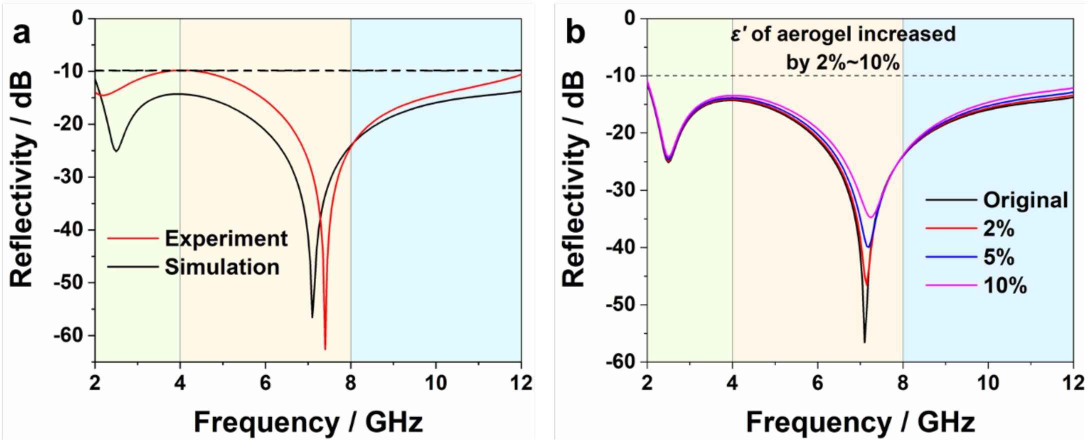

Fig. 8 (a) Comparison of the experimental reflectivity and simulated reflectivity of the MTL integrated composites. (b) The simulated reflectivity of the MTL integrated composites when the ε' of the aerogel increases by 2%, 5%, and 10%.

Revised version: Fig. 7 and Fig. 8b has been added to Supplementary file as **Supplementary Fig. 4** and **Supplementary Fig. 3a**. The related revised text can be found in P9&P12 of revised manuscript:

P12 (**Supplementary Fig. 4**): To qualitatively clarify this EM response mechanism, the frequency-dependent curves of Z_{in1} to Z_{in6} (**Supplementary Fig. 4**) are provided, where Z_{in3} and Z_{in5} correspond to the impedance characteristics of the lower and upper metasurfaces, respectively. In S-band, the upper metasurface shows wave-transparent characteristics without microwave loss (**Fig. 2g**), whereas the lower metasurface achieves better impedance matching, resulting in perfect EM wave absorption. In X-band, the real part of Z_{in3} approaches zero, while the real part of Z_{in5} approaches 1 (**Supplementary Fig. 4**). This results in the lower metasurface exhibiting EM reflective behavior, while the upper metasurface achieves excellent

impedance matching, thus enabling effective microwave absorption. For the C-band, the transmission, reflection and absorption characteristics for EM wave of the two metasurfaces lie between those in the above two cases.

P9 (**Supplementary Fig. 3a**): It is worth noting that the EAB of this microwave-absorbing structure exhibits high robustness to these structural parameters (**Supplementary Fig. 3**), implying that fabrication tolerances are allowed.

Q4: The authors attribute the dominant loss mechanism to conductor (resistive) loss. However, aerogel dielectric loss (tan*δ*) also increases significantly at high temperatures. Are there experiments or modeling that quantitatively separate the contributions of dielectric vs. resistive losses?

Answer: We thank the reviewer for this comment. In fact, the tan*δ* of the aerogel only increase by 3.75% (from 0.008 to 0.0083, based on the data of 10 GHz) from room temperature to 1000 °C (**Supplementary Fig. 1**). To evaluate the influence of the aerogel's loss capability on the microwave absorption performance of the MTL integrated composite, we simulated the reflectivity by setting the tan*δ* of aerogel to zero and compared it with the original reflectivity curve (**Fig. 9**). The results show almost no changes in the EAB, the number of resonance peaks, or their positions, with highly overlapping curves. This indicates that the loss contribution of the aerogel can be negligible. Therefore, we conclude that the dominant loss mechanism in the MTL integrated composite is conductive loss.

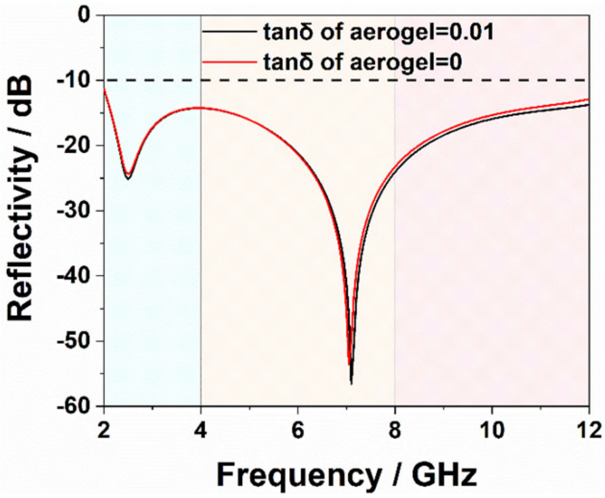

Fig. 9 Reflectivity of the MTL integrated composites when the tanδ of the aerogel are 0.01 and 0.

Q5: The temperature-dependent dielectric properties (ε', tanδ) are measured using cavity perturbation. However, this technique is known to suffer from increasing error at high temperatures. Have independent methods such as waveguide transmission or THz-TDS been used to validate these measurements?

Answer: We thank the reviewer for this comment. Your suggestions are very pertinent. We measured the complex permittivity of the Al_2O_{3f}/Al_2O_3 ceramic composites and the alumina aerogel composites in the X-band from 25 °C to 1000 °C via the waveguide method. No observable frequency dispersion was found in their complex permittivity, meaning that it remained nearly unchanged across the frequency band. This is demonstrated, for example, by the data for the Al_2O_{3f}/Al_2O_3 ceramic composites at 25 °C and the alumina aerogel composites at 1000 °C (**Fig. 10c** and **10d**).

We used data from the center frequency of the X-band (10 GHz) to plot the temperature-dependent variations in ε' and tanδ. According to **Fig. 10a**, temperature

has almost no effect on the ε' of the Al_2O_{3f}/Al_2O_3 ceramic composites or alumina aerogel composites. The tanδ values of both materials initially slightly decrease with increasing temperature but recover to the room-temperature level by 1000 °C (**Fig. 10b**). Additionally, we have replaced **Supplementary Fig. 2** with the waveguide measurement data.

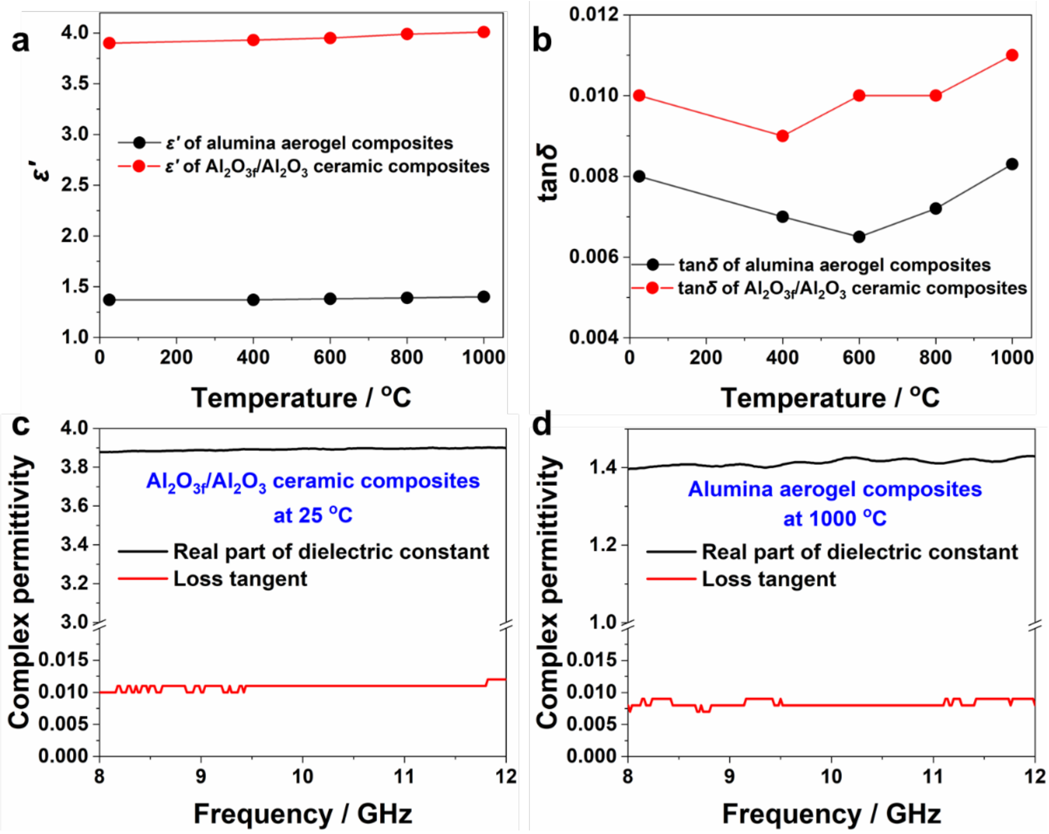

Fig. 10 High-temperature (a) dielectric constant (ε') and (b) loss tangent (tanδ) of alumina aerogel composites and Al_2O_{3f}/Al_2O_3 ceramic composites at 10 GHz. The frequency dependence of the complex permittivity of the (c) Al_2O_{3f}/Al_2O_3 ceramic composites at 25 °C and (d) alumina aerogel composites at 1000 °C.

Revised version: Fig. 10a and Fig. 10b has been added to Supplementary file as **Supplementary Fig. 1.** The related revised text can be found in P8&P29 of revised manuscript:

P8: As input for simulation, the dielectric constants and loss tangents of the alumina aerogel composites and the Al_2O_{3f}/Al_2O_3 ceramic composites were measured using the waveguide method (**Supplementary Fig. 1**).

P29: To meet the EM simulation requirements, the complex permittivity of the alumina aerogel composites and Al_2O_{3f}/Al_2O_3 ceramic composites at 25~1000 °C were measured with a vector network analyzer (VNA, N5225B, Keysight Technologies, USA) using the waveguide method.

Q6: The EM absorption after thermal shock is highly recommended.

Answer: We thank the reviewer for this comment. We tested the microwave absorption performance of the MLT integrated composites after 1~50 cycles of thermal shock, as shown in Figure 4e in the manuscript.

Q7: Fig. 3f and g show the peak around 850 °C. An explanation is encouraged.

Answer: We thank the reviewer for this comment. We think that the increase in resistance observed in the RuO_2/glass resistive patch at 800~900 °C originates from the phase transition of glass matrix. The endothermic peak in the DSC curve of the glass (**Fig. 11**) within this temperature range indicates a phase transition. The phase transition of the glass matrix may induce a change in its CTE or TCR, thereby leading to the increase of barrier resistance. Additionally, RuO_2 possesses a positive intrinsic TCR, meaning that its intrinsic resistance also increases with increasing temperature. Consequently, at 800~900 °C, both the intrinsic resistance and the barrier resistance increase, ultimately leading to an overall increase in the resistance of the RuO_2/glass resistive patch.

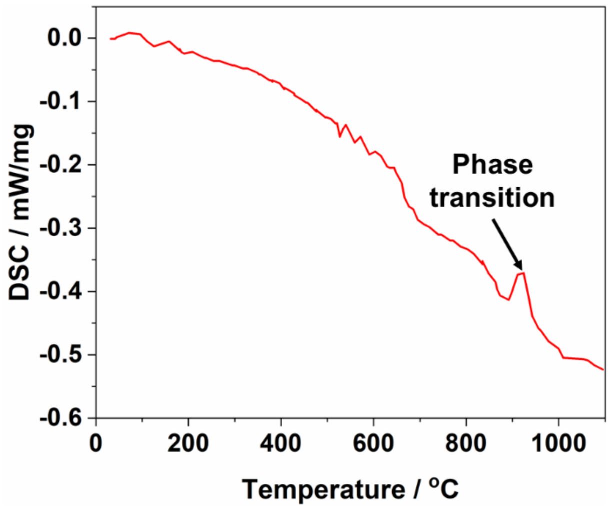

Fig. 11 DSC curve of the glass from room temperature to 1100 °C.

Revised version: Fig. 11 has been added to Supplementary file as **Supplementary Fig. 10**. The related revised text can be found in P16 of revised manuscript and P5 of revised supplementary file:

P16 of revised manuscript: Although the normalized resistance of the RuO_2/glass resistive patch increases at 800~900 °C (may be due to the phase transition of the glass matrix (**Supplementary Fig. 10**)), it still has negative resistance–temperature characteristics from 25~1000 °C, with a TCR of -95~-365 ppm/°C (**Figs. 3f** and **3g**).

P5 of revised supplementary file: Simultaneous thermal analysis (STA 449C, Netzsch Group, Germany) was employed to characterize the differential scanning calorimetry (DSC) of the glass powder from room temperature to 1100 °C at a heating rate of 5 °C/min.

References

1. Chen C-L*, et al.* Fabrication of tantalum silicate films with low TCR and low resistivity by magnetron sputtering. *Vacuum* **226**, 113330 (2024).

2. Zhang B, Sui J, Kou Q, Kang X, Zhou D. Tuning the structural and electrical properties of TiNx thin films with low temperature coefficient of resistance. *Applied Surface Science* **706**, 163553 (2025).

3. Lukose CC, Zoppi G, Birkett M. Mn3Ag(1-x)Cu(x)N antiperovskite thin films with ultra-low temperature coefficient of resistance. *Journal of Materials Science & Technology* **99**, 138-147 (2022).

4. Chen Y, Zhang J, Gou H, Wang X, Chen Y, Guo H. Tantalum nitride thin film with a nearly zero temperature coefficient of resistance synthesized by magnetron co-sputtering. *Vacuum* **222**, 113092 (2024).

5. Yuan X*, et al.* A Delicate Balance Between Spin-Wave Mediated Weak Localization and Electron-Phonon Scattering in the Design of Zero Temperature Coefficient of Resistivity. *Small* **20**, 2311599 (2024).

6. Peng F, Jiang Y, Feng J, Cai H, Feng J, Li L. Thermally insulating, fiber-reinforced alumina–silica aerogel composites with ultra-low shrinkage up to 1500 °C. *Chemical Engineering Journal* **411**, 128402 (2021).

7. Guo J*, et al.* Hypocrystalline ceramic aerogels for thermal insulation at extreme conditions. *Nature* **606**, 909-916 (2022).

8. Xu X*, et al.* Double-negative-index ceramic aerogels for thermal superinsulation. *Science* **363**, 723-727 (2019).

9. Koebel M, Rigacci A, Achard P. Aerogel-based thermal superinsulation: an overview. *Journal of Sol-Gel Science and Technology* **63**, 315-339 (2012).

10. Lee D, Stevens PC, Zeng SQ, Hunt AJ. Thermal characterization of carbon-opacified silica aerogels. *Journal of Non-Crystalline Solids* **186**, 285-290 (1995).

11. Xie Y, Xu S, Xu Z, Wu H, Deng C, Wang X. Interface-mediated extremely low thermal conductivity of graphene aerogel. *Carbon* **98**, 381-390 (2016).

12. Wu J-W, Sung W-F, Chu H-S. Thermal conductivity of polyurethane foams. *International Journal of Heat and Mass Transfer* **42**, 2211-2217 (1999).

Response to reviewer 3#:

Q1. How did the authors measure permittivity using the VNA method at such high temperatures?

Answer: We think the reviewer for the comment. The high-temperature waveguide testing equipment used in this study is illustrated in **Fig. 1**. Initially, the sample is placed inside the waveguide cavity (**Fig. 1a** and **1b**). The waveguide cavity is then positioned within a high-temperature furnace. Once the target temperature is reached and stabilized, the dielectric constant of the sample at high temperature is promptly measured.

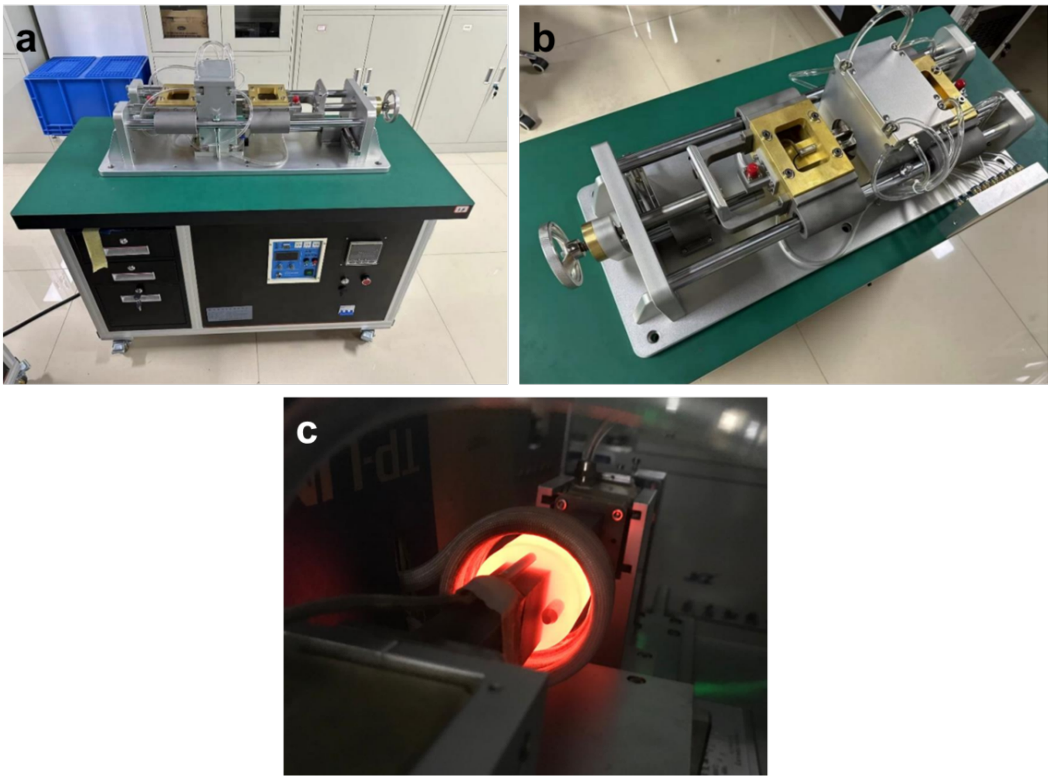

Fig. 1 High-temperature waveguide transmission reflection method testing equipment.

Q2. How thick is of RuO_2/glass fiber layer coating?

Answer: We think the reviewer for the comment. According to the statistical analysis of optical microscopy images (**Fig. 2**), the printed RuO_2/glass resistive coating

exhibits a thickness of ~ 22 μm.

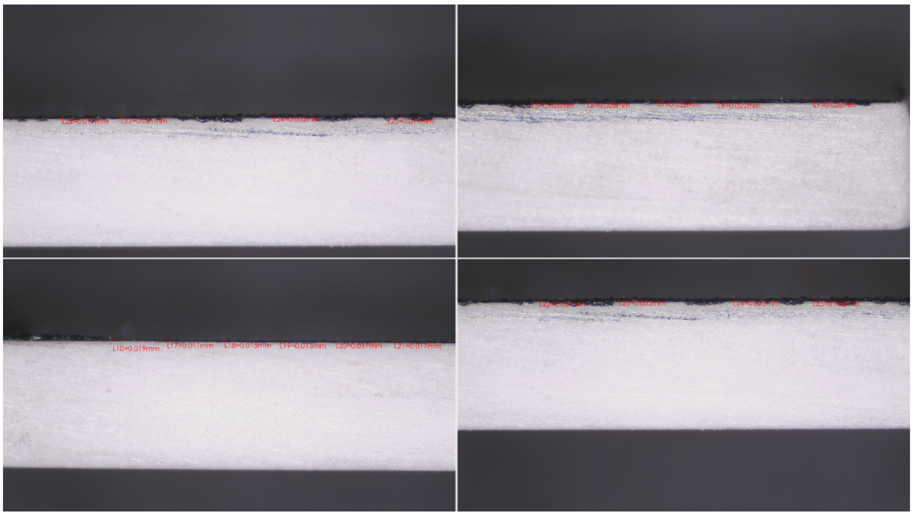

Fig. 2 Optical microscopy images of RuO_2/glass resistive coating on alumina substrate.

Q3. It is great if the reflectivity properties of the single resistive layer on Al_2O_{3f}/Al_2O_3 are provided.

Answer: We think the reviewer for the comment. According to the absorption principle of metasurfaces, they must rely on dielectric space for electromagnetic resonance. For a single resistive layer on Al_2O_{3f}/Al_2O_3, its reflectivity is higher than -0.2 dB (**Fig. 3a**), indicating a lack of absorption capability. Simultaneously, we provide the reflection coefficient (S11) and transmission coefficient (S21) of the two-layer metasurface itself (**Fig. 3b**).

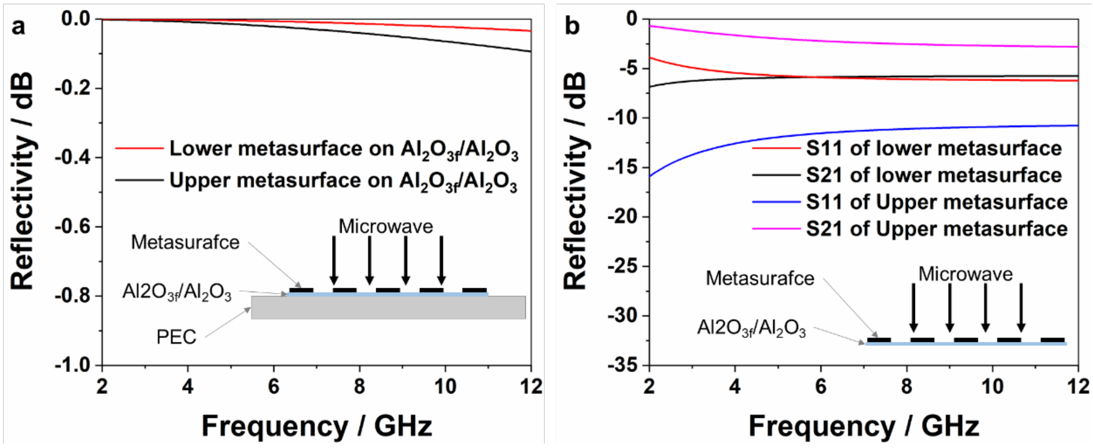

Fig. 3 (a) Reflectivity of single resistive layer on $Al_2{\cdot}O_{3f}/Al_2O_3$. (b) Reflection coefficient and transmission coefficient of the two-layer metasurface itself.

Q4. The unit of thermal conductivity in Fig. S21a may be wrong.

Answer: We think the reviewer for the comment. We have revised the unit of thermal conductivity in Fig. S21a to mW/(m·K) (**Fig. 4**).

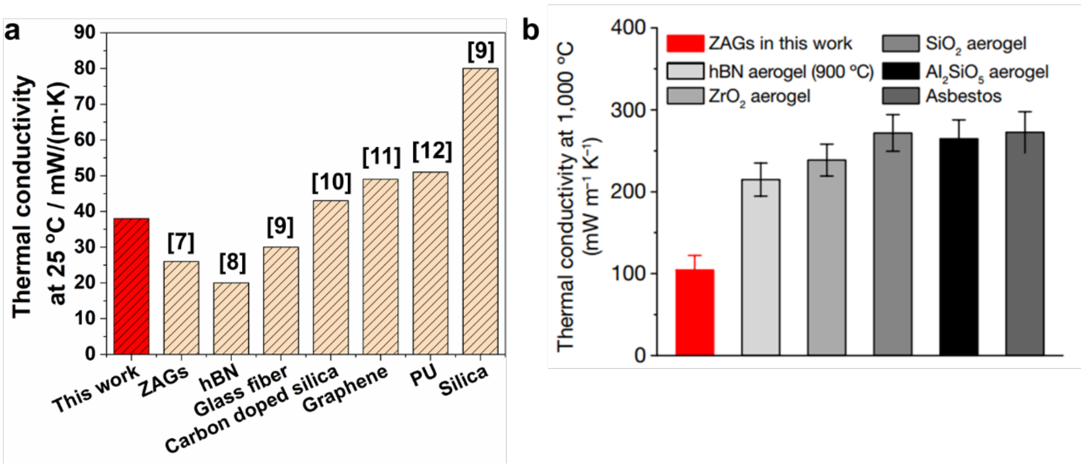

Fig. 4 (a) Comparison of the thermal conductivities of the alumina aerogel composites with those of other aerogels at 25 °C. (b) Thermal conductivity of other aerogels at 1000 °C (the picture is from reference).

Q5. References are needed in lines 46-48, 56-59, 71-73.

Answer: We think the reviewer for the comment. We have added references at these position.

Lines 46-48: However, existing MAMSs maintain stable microwave-absorbing performance exclusively in low-temperature environments (typically ≤500 °C)[14, 15].

Lines 56-59: For resistive materials that absorb microwave through electrical losses, the temperature greatly influences both their carrier transport rate and dipole relaxation time, thereby inducing markedly variations of their electrical properties along with temperatures[16].

Lines 71-73: To reduce the variations of electrical properties of MAMSs at elevated temperatures, a natural and general strategy is to lower the temperature of metasurfaces using heat-insulating materials as their dielectric spacer layer[18, 19].

Q6. In lines 76-81, the authors have an interesting comment on how absorption and cooling performance rely on thickness. Is it solved in this work?

Answer: We think the reviewer for the comment. The issue has been solved in this work. Conventional metasurfaces often require a trade-off between cooling efficiency and microwave-absorbing performance due to insufficient high-temperature resistance and oxidation resistance. In contrast, the metasurface developed in this work exhibits exceptional thermal stability and oxidation resistance, enabling reliable operation under high-temperature conditions. Therefore, in designing the microwave-absorbing structure, we can focus solely on optimizing absorption performance without the need to consider cooling effects.
